# First-Principles Thermodynamic Background of the Comprehensive Reaction Network of NO Oxidation over CuSSZ-13 Catalysts—Influence of Copper Speciation and Interpretation of TPD and TPSR Profiles

**DOI:** 10.1021/acscatal.4c06619

**Published:** 2025-01-30

**Authors:** Bartosz Mozgawa, Filip Zasada, Monika Fedyna, Kinga Góra-Marek, Chengyang Yin, Zhen Zhao, Zbigniew Sojka, Piotr Pietrzyk

**Affiliations:** † Faculty of Chemistry, Jagiellonian University, ul. Gronostajowa 2, Krakow 30-387, Poland; ‡ Doctoral School of Exact and Natural Sciences, Jagiellonian University, ul. prof. S. Łojasiewicza 11, Krakow 30-348, Poland; § Institute of Catalysis for Energy and Environment, College of Chemistry and Chemical Engineering, Shenyang Normal University, Shenyang, Liaoning 110034, China

**Keywords:** CuSSZ-13 zeolite, NO oxidation mechanism, HONO, ab initio thermodynamics, stability diagrams, EPR, IR, DFT modeling

## Abstract

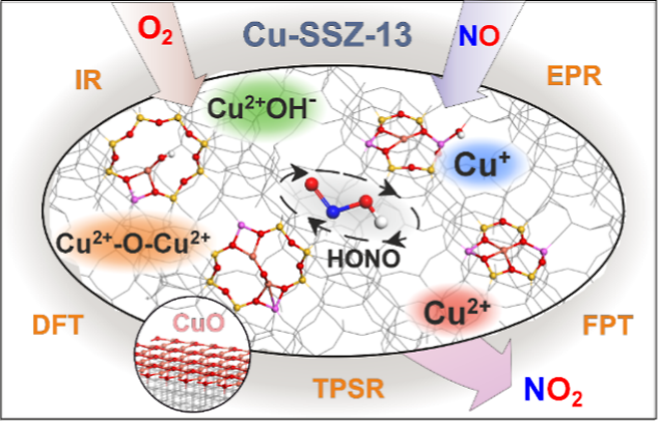

A thorough molecular DFT modeling coupled with first-principles thermodynamic (FPT), spectroscopic (EPR/IR), and catalytic investigations into a complex network of reactions involved in the interaction of NO and O_2_ with a comprehensive variety of active centers present in the CuSSZ-13 zeolites (Cu^2+^, Cu^+^, Cu^2+^–OH^–^, Cu^2+^–O^2–^–Cu^2+^, Cu^2+^–O_2_^2–^–Cu^2+^, and segregated CuO) were carried out. The molecular structure, energetics, and electronic and magnetic properties of the identified profuse adspecies and intermediates were ascertained. Their thermal stability and reactivity at a wide range of experimental conditions were interpreted by using the constructed thermodynamic Δ*G*(*p*,*T*) diagrams. The course of selective catalytic oxidation of NO (NO–SCO) with ^16^O_2_ or ^18^O_2_ was examined by a temperature-programmed surface reaction (TPSR) using two types of CuSSZ-13 catalysts of intentionally diverse copper speciation. The results obtained, supported by the corroborative IR and EPR measurements, revealed multiple molecular pathways of the NO and O_2_ interactions with the single (Cu^2+^, Cu^+^, Cu^2+^–OH^–^) and dual (Cu^2+^–O^2–^–Cu^2+^, Cu^2+^–O_2_^2–^–Cu^2+^) copper centers of the 6MR and 8MR topologies and with segregated CuO. The complex reaction network and temperature behavior of the critical intermediates (HONO, nitrate, and nitrite), and their evolvement routes into NO_2_, were rationalized using the calculated FPT thermodynamic profiles. The unraveled reactions were classified into metal (cationic) redox, ligand (anionic) redox, and HONO redox cycles. The Cu^2+^–OH^–^ species were identified as prime active centers for the formation of NO_2_ via the HONO pathway. The elusive HONO intermediates allow for chemical communication between the individual redox cycles. Depending on the actual reaction conditions, HONO can act as a reduction agent for Cu^2+^ with the electroprotic formation of NO_2_, a source of nitrites upon deprotonation, or as an oxidant of Cu^+^ with the formation of H_2_O and NO. For the metal redox pathway, a significant difference in the reactivity between the Cu^2+^ cations accommodated in the 6MRs and 8MRs was observed, with the Cu^2+^/6MR being spectators and the Cu^2+^/8MR active species. Dimeric copper centers with bridging oxo and peroxy moieties can produce a variety of nitrates and nitrites via ligand redox mechanisms. Segregated CuO nanocrystals contribute to NO oxidation only at high temperatures (*T* > 400 °C), leading to the isotopic scrambling of ^18^O-labeled oxygen and nitric oxide. The established complex reaction network was successfully used to clarify the temperature dependence of the experimental NO–SCO profiles, also providing a suitable mechanistic background for interpreting the nature of the oxidative half-cycle of the selective catalytic reduction of NO over Cu-SSZ-13 catalysts.

## 1 Introduction

Among many catalytic systems applied for the abatement of NO_*x*_ from diesel sources based on NH_3_-assisted selective catalytic reduction (NH_3_–SCR),^1−4^ the narrow pore CuSSZ-13 zeolites are of particular importance.^5−8^ We have recently outlined the temperature regions of the NH_3_–SCR reaction, the prime mechanistic pathways, and the associated intermediates of each region, revealing an inherent trade-off between the concurrent NO reduction and NH_3_ oxidation processes.^9^ Since early studies, the redox character of these reactions has been recognized, and the catalytic performance of CuSSZ-13 relies on reversible changes in the oxidation state of copper active centers upon interaction with NO, NH_3,_ and O_2_.^10−16^ Although the general basis for the low- and high-temperature NH_3_–SCR mechanism has been established, certain molecular events remain elusive and are the subject of numerous ongoing investigations.^17−19^ They refer particularly to the mechanistic pathways of NO oxidation and formation of nitrates/nitrites,^9−12,20^ which are involved in the oxidation half-cycle (OHC),^20−22^ and are facilitated over copper ion pairs.^22−24^

EPR and IR investigations supported by density functional theory (DFT) modeling have revealed that Cu^+^ cations are readily reoxidized to Cu^2+^ in the mixture of NO and O_2_ to produce Cu^2+^NO_*x*_^–^ species.^17,25^ They are also formed when the CuSSZ-13 catalyst is exposed to NO alone or a mixture of NO, O_2_, and NH_3_. In the latter case, NH_3_ has been argued to not participate in OHC directly, yet the NH_3_/NH_4_^+^ species can help to reduce NO_3_^–^/NO_2_^–^ into N_2_ and H_2_O.^3,9,11^ The activation barriers calculated by DFT reported for Cu^+^ oxidation, jointly by NO and O_2_, are in the range of 55–70 kJ/mol for the NO_2_/NO_2_^–^ adspecies, increasing to 96 kJ/mol in the case of NO_3_^–^, and are not significantly affected by the inclusion of the NH_3_ ligation in the calculations.^17^ These barriers are comparable to experimental values of 70–80 kJ/mol for single copper sites and for dimer adducts,^7^ implying that the oxidation of Cu^+^ within the chabazite framework is relatively straightforward (at least at *T* > 350 °C). Interestingly, nitrates and nitrites are formed over CuSSZ-13 catalysts even when fully oxidized before contacting the NO/O_2_ mixture.^17^ The Cu^2+^(OH)^−^/Z and (O_2_^–^)Cu^2+^/Z species have been reported to be involved in this reaction, while the Cu^2+^/Z_2_ sites are completely inert.^25^

Selective catalytic oxidation of NO to NO_2_ (NO–SCO) is often considered one of the reference reactions used in mechanistic investigations into the OHC of the SCR course.^26,27^ It has been hypothesized that only the dimer copper centers can dissociate O_2_, which is required for NO oxidation in a kinetically relevant step.^24^ These findings imply that NO–SCO can indirectly probe the clustering of Cu ions in Cu-SSZ-13. However, although for highly loaded CuSSZ-13 samples that exhibit enhanced SCR activity, the appearance of two closely spaced copper Cu^2+^–OH^–^ cations or bridging [Cu–O–Cu]^2+^ entities can be expected, no consensus has been reached on the mechanistic relevance of such bridging species for the NO and NH_3_ oxidation reactions that are associated with NH_3_–SCR. The presence of [Cu–O–Cu]^2+^ oxo entities, mainly in dehydrated samples with Cu loading >1 wt %, has been documented by EXAFS^10^ and Raman investigations.^28^ Unlike the isolated copper centers, such dimeric species are capable of NO_*x*_^–^ formation even in their oxidized state and, therefore, should be treated carefully in the mechanistic considerations of the SCO (and OHC) processes.^1,2,4^

So far, NO_*x*_ interaction with single monovalent Cu^+^ cations located at exchangeable sites of the SSZ-13 framework has been modeled by DFT combined with the first-principles thermodynamics (FPT).^29−31^ NO, NO_2_, and NO_3_ binding to Cu^2+^ and Cu^+^ in various accommodation topologies has been tested. In the case of NO coordination, the initial oxidation state of Cu^2+^ remains intact. In contrast, the ligation of NO_2_ and NO_3_ to Cu^+^ cations leads to the formation of the corresponding ligand anions with the concomitant oxidation of the copper center. The Cu^2+^NO_3_^–^ adducts produced upon NO and O_2_ coadsorption are the most stable in the wide range of the O_2_ and NO partial pressures. Direct oxidation of NO by O_2_ upon coordination to Cu^+^ has been reported to involve significant activation energy of 95–110 kJ/mol, while the formation of nitrites by the oxidation of dinitrosyl adducts Cu^+^(NO)_2_ appears less energetically demanding (55–75 kJ/mol).^21^

Although the NO_3_^–^/NO_2_^–^ adspecies are frequently observed spectroscopically during NO–SCO and SCR by operando IR,^32−34^ the variety of the molecular routes along which they can be formed upon the interaction of NO and O_2_ with reduced and oxidized Cu centers of various nuclearity, and their thermodynamic stability under conditions similar to the actual SCO/SCR processes, have not been fully explored so far. The latter is especially valid for the Cu^2+^–OH^–^ species at the chabazite 8-membered rings (8MRs) and the dimeric [Cu–O–Cu]^2+^ entities. The mechanistic role of dimeric/polymeric species in SCO gains importance for catalysts with moderate Si/Al ratios and moderate copper loadings.^35^ In particular, the [Cu–O–Cu]^2+^ species are expected to be the active centers for the oxidation of NO to NO_2_, which is particularly beneficial for the fast SCR reaction.^26,36^ They also activate the N–H bond during the reductive half-cycle stage.^9,37^

In this paper, we performed a comprehensive molecular and atomistic thermodynamic modeling based on the DFT calculations of the NO and O_2_ interactions with all conceivable copper centers of various nuclearity and topology, stabilized in the exchangeable sites of the SSZ-13 framework, and for segregated intrazeolitic CuO nanocrystals, which often appear in the catalyst prepared by solid-state exchange or upon aging. However, we neglected NO oxidation on Brønsted acid sites (BAS) since this issue has been elaborated elsewhere in detail.^38,39^ We aimed to provide a thermodynamic feasibility study for interpreting the complex network of the NO selective catalytic oxidation (NO–SCO) reactions by unraveling meaningful pathways of NO_*x*_ generation, redox reactivity patterns, and evolution of intermediates under NO oxidation conditions. Molecular DFT modeling and FPT results were supported by spectroscopic IR and EPR studies (identification of the postulated intermediates and copper redox state monitoring) and by temperature-programmed selective catalytic oxidation of NO using ^18^O isotope labeling (determination of the temperature windows of NO oxidation and adsorption/desorption of NO_*x*_ species). Such an approach allowed us to establish a comprehensive network of reactions involved in NO oxidation and the temperature ranges of their occurrence. Due to the vast number of chemical reactions examined, we did not address complementary kinetic analysis in this work.

## 2 Materials and Methods

### 2.1 CuSSZ-13 Zeolites

Two types of CuSSZ-13 zeolites that differ distinctly in the Si/Al ratio, the Al sites’ distribution, and the copper introduction method were examined. The sample prepared by impregnation of NH_4_SSZ-13 with a solution of CuCl_2_ is labeled *i*-CuSSZ-13 (Si/Al = 7.9 with 70% of the Al atoms as single sites, total Cu content 780 μmol/g) and that prepared by one-pot synthesis as *o*-CuSSZ-13 (Si/Al = 3.4, total Cu content 590 μmol/g). Detailed descriptions of the synthesis protocols and characterization results of these samples have been provided in our previous articles,^9,40^ including quantification of all types of the copper active sites: Cu^2+^ (330 and 140 μmol/g), Cu^2+^–OH^–^ (30 and 255 μmol/g), and Cu^2+^–O^2–^–Cu^2+^ (220 μmol/g and an undetermined amount) for *o*-CuSSZ-13 and *i*-CuSSZ-13, respectively. The latter sample contained 325 μmol/g of nano-CuO/Cu-oxo species in total, while the concentration of Brønsted sites was equal to 600 μmol/g (*o*-CuSSZ-13) and 160 μmol/g (*i*-CuSSZ-13). Furthermore, from analysis of the EPR parameters of the divalent copper in *o*-CuSSZ-13, 80% of Cu^2+^ occupies 6MR, whereas 20% occupies 8MR sites. In the case of *o*-CuSSZ-13, these occupancies are equal to 54 and 46%, respectively.

### 2.2 Spectroscopic Methods

IR measurements of NO/O_2_ adsorption/desorption were carried out on a Bruker Vertex 70 spectrometer equipped with an MCT detector using self-supporting pellets placed in a heated quartz cell with KBr windows. Before IR measurements, the samples were dehydrated at 250 °C for 30 min under a vacuum (10^–4^ mbar residual pressure) and then contacted with oxygen (60 Torr in the gas phase). The temperature was then increased to 550 °C and then was kept constant for 1 h. Finally, the samples were outgassed and cooled to room temperature. The spectra, recorded with a resolution of 2 cm^–1^, were normalized to the standard pellet mass (10 mg and a density of 1.2 mg/cm^2^).

EPR spectra were recorded by using a Bruker ELEXSYS-E500 X-band spectrometer equipped with a rectangular TE_102_ cavity that operates at 100 kHz field modulation. All adsorption and thermal treatment experiments were performed by using a 10^–4^ mbar residual pressure vacuum line. The samples were sealed in quartz ampules and thermally treated under a vacuum, followed by activation in O_2_ at 500 °C, cooling, and final evacuation. All of the in situ reactions in the EPR experiments were carried out at realistic *T* and *p* conditions, and only recording of the signals was performed upon quenching the samples at 77 K for better resolution of the spectral features.

### 2.3 TPD and TPSR Measurements

Before temperature-programmed experiments, the samples (40 mg) were thermally activated under a flow of 4.5% O_2_ in He at 500 °C for 1 h (with a heating rate of 10 °C/min). All experiments were carried out in a quartz fixed bed reactor coupled with a QMS detector (Hiden Analytical HPR20) for the quantification of N^16^O (*m*/*z* = 30), N^16^O_2_ (*m*/*z* = 46), N_2_^16^O (*m*/*z* = 44), N_2_ (*m*/*z* = 28), ^16^O_2_, N^18^O (*m*/*z* = 32), ^18^O_2_ (*m*/*z* = 36), and ^16^O^18^O (*m*/*z* = 34) species. The concentrations of the reagents and reaction products were also measured by using an IR spectrometer (Thermo Fisher, Nicolet iS20) equipped with a gas cuvette (2 m optical path length).

The NO–SCO reaction was carried out at 100–550 °C with a heating rate of 10 °C/min. The gas phase contained 500 ppm of NO and 4.5% of O_2_, with He as the gas balance and 60 mL/min flow.

### 2.4 DFT and FPT Calculations

SSZ-13 zeolite sites that host copper in the exchange positions were calculated using the VASP (Vienna Ab initio Simulations Package) software^41^ and the projector augmented wave (PAW) method.^42^ The structure of the SSZ-13 zeolite was modeled with a hexagonal unit cell of the O_72_Si_34_Al_2_Cu_*x*_ stoichiometry (*x* = 1 for mono- and 2 for dual copper sites). The relaxed cell size (*a* = *b* = 13.778 Å, *c* = 14.879 Å) was obtained using the Birch–Murnaghan method.^43^ The applied Al distribution in Cu-SSZ-13 lattice was motivated by the recent papers of Paolucci et al.^44^ and Schneider et al.^45^ and also by our supplementary quantum chemical analysis (see Chapter S1 in Supporting Information for details).

Geometry optimization was done within spin-polarized, plane-wave periodic DFT calculations. The plane-wave cutoff energy was set to 500 and 450 eV for the core and valence states, respectively. Gamma sampling of the Brillouin zone and the Methfessel-Paxton smearing parameter of σ = 0.05 eV were used.^46^ The electronic energies converged to 10^–6^ eV, while the geometric relaxation was set at 0.01 eV/Å. Dispersion forces were included within the Tkatchenko and Scheffler method (DFT-TS).^47^ Optimized structures of the SSZ-13 zeolite were obtained with the employment of the PW91 functional^48^, corrected by the Hubbard method (GGA + *U*)^49^ with the *U* parameter set to 4.0 eV for the Cu ions. The arguments for selecting this level of theory, the values of the key scaling parameters, and the results of benchmark and comparative computational experiments are included in Supporting Information; see Chapter S2.

The interaction of O_2_ and NO with copper(II) oxide nanocrystals was modeled using a periodic slab exposing the (111) surface, as previously revealed by TEM imaging.^9^ The constructed Cu_80_O_80_ supercell with ∼9.5 Å thickness (corresponding to five CuO layers) and vacuum layer of 10 Å is shown in Figure S3 (Supporting Information). The DFT calculations were performed at the same level of accuracy as for the zeolites but with *U* = 7 eV, which ensures the proper reproduction of geometric and electronic properties of the examined CuO nanocrystals.^50^

Interaction of the copper active sites accommodated in CuSSZ-13 with NO, O_2_, and H_2_O molecules under various *p*, *T* conditions was studied using FPT.^51^ In this approach, the Gibbs free energy of adsorption (*G*^a^) is expressed as a function of temperature (*T*), partial pressure (*p*_m_), and the number of adsorbed molecules (*n*_i_^m^), according to the general formula
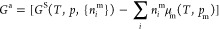
1

Following the literature, the Gibbs free energy of the substrate (*G*^S^ in general or G^Z^ and *G*^O^ for SSZ-13 zeolite and CuO oxide, respectively) interacting with the reactants can be approximated as *G*^S^ ≈ *E*_DFT_^S^, given that the substrate oscillations remain nearly unchanged upon adsorption/desorption of the reactants.^52,53^ The chemical potentials of the gaseous reactants, μ_m_(*T*, *p*_m_), related to *T* and *p*_*m*_ through the ideal gas equation, were calculated as the sum of the total DFT energy component, *E*_m_^tot^, of the gaseous molecules, and the standard term Δμ_m_^0^(*T*, *p*_m_), which comprises the contribution related to *T* and *p*
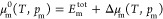
2
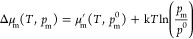
3

To include the effects of space confinement (imposed by the channels of the SSZ-13 structure), a scaling factor of 0.85 was applied to the calculated Gibbs free energies of the gaseous molecules. This value was empirically selected from our previous calculations of the interaction of NH_3_ with CuSSZ-13 (ref (40), and references therein).

Thermodynamic profiles of the investigated reactions (ξ) were constructed based on the calculated equilibrium constants (*K*_*j*_) for each partial reaction using the following formula^38^
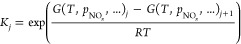
4

The obtained values were then normalized from 0 to 1
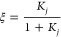
5

## 3 Results and Discussion

### 3.1 Molecular Structure and Energetics of the NO_*x*_^δ−^ and O_2_ Adducts with Cu Sites

The conceivable copper active centers present in the CuSSZ-13 zeolite include Cu^2+^, Cu^+^, and Cu^2+^–OH^–^ single copper centers, bridging dual copper Cu^2+^–O^2–^–Cu^2+^, Cu^2+^–O_2_^2–^–Cu^2+^, and Cu^2+^–(OH^–^)_2_–Cu^2+^ species and dual copper cations in the vicinal positions Cu^+^∪Cu^+^. Their optimized structures are shown in Figure S4. They are hosted in the 6-membered rings (6MRs) and 8MRs rings, also referred to as σ and τ-sites, respectively.^54^ Their energies, electronic and magnetic parameters, and oxidation states are summarized in Section S4.1 and Table S2. These parameters are complemented by analogous structural results for the dual Cu centers (Section S4.2), which also include the thermodynamic conditions of their formation.

Next, the structure and energetics of adsorbed NO_*x*_^δ−^ species, resulting from various stoichiometries of the NO and O_2_ reactants interacting with the single (Section S4.1) and dual Cu centers (Section S4.3), were modeled. Noting that the corresponding NO_*x*_^δ−^ adducts have previously been the subject of numerous DFT studies,^13,17,19,29,33,37,40^ their detailed structural description and assignment of the oxidation and spin states are presented as additional information and moved to Section S4 (in Supporting Information). In contrast, the thermodynamic analysis of the interaction of all types of Cu centers with the NO/O_2_ reactants is discussed below.

### 3.2 Thermodynamics of NO/O_2_ Interaction with Single Copper Sites

#### 3.2.1 Formation and Stability of NO_*x*_^δ−^ Adducts with Single Cu Ions

**Figure 1:**
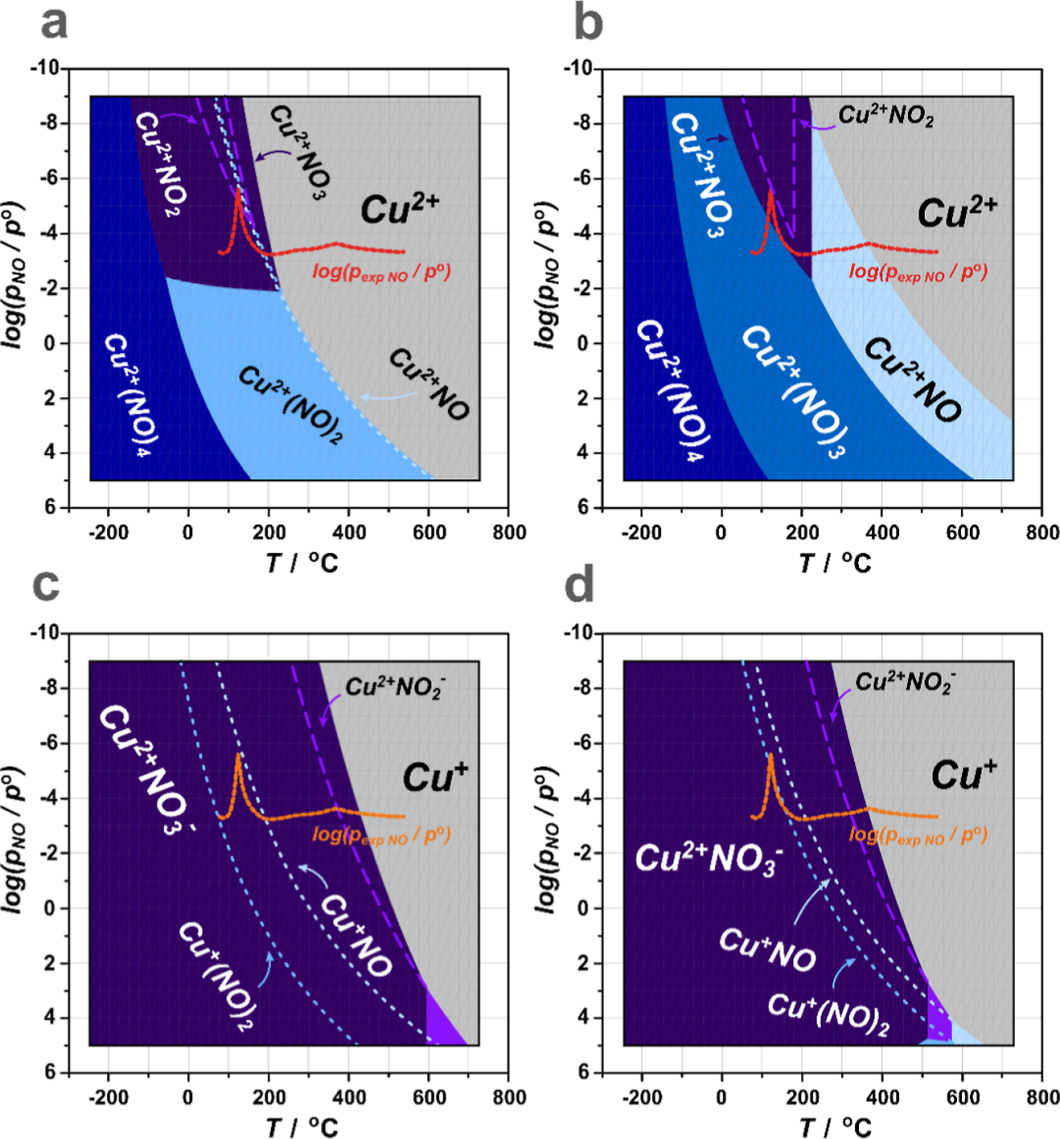
Thermodynamic stability diagrams for Cu^2+^ (a,b) and Cu^+^ (c,d) cations interacting with NO and O_2_, accommodated at the 6MR (a,c) and 8MR (b,d) sites, as a function of temperature (*T*) and partial pressure of NO (*p*_NO_) with the constant level of O_2_ (4.5%). The dashed and dotted lines represent intersections of the energy planes of less stable structures for Δ*G*_r_ = 0 defined for the bare Cu^2+^ and Cu^+^ centers. The dotted red and orange lines correspond to the experimentally determined changes in the NO pressure during the NO–SCO reaction (*p*_exp,NO_).

The relative stability of the copper-NO_*x*_^δ−^ anionic species and copper-radical species formed after the reaction between NO and O_2_ on the reduced and oxidized CuSSZ-13, respectively, was examined using FPT modeling. The results are summarized in the form of 2D phase diagrams for the oxidized and reduced copper species located in the 6MR and 8MR sites as a function of temperature (*T*) and partial pressure of NO (*p*_NO_), with the pressure of the O_2_ set at the experimental value of 4.5% (Figure 1).

In the absence of water (in analogy to SCO experimental conditions), radical (δ ≪ 1) and anionic (δ ∼ 1) adducts NO_*x*_^δ−^ can be produced, and the regions of their stability are shown in Figure 1 for Cu^2+^ and Cu^+^ at both 6MR and 8MR sites.

Even at a relatively high experimental partial pressure of O_2_ (4.5 × 10^–2^), compared to the value of *p*_NO_ ∼ 10^–3^, the isolated oxidized Cu^2+^ centers are unable to capture dioxygen, and the thermodynamic diagrams are dominated by various NO_*x*_^δ−^ adducts (*x* = 1–3) of the radical nature (Figure 1a,b). However, for Cu^2+^/6MR sites at higher NO concentrations (Figure 1a), nitrosyls exhibit stability similar to that of the NO_*x*_^δ−^ adspecies. Under conditions similar to the NO oxidation experiment, represented by red dotted lines (variation of *p*_exp,NO_ during the catalytic NO–SCO tests, see Section 3.4.3), for the 6MR sites, the stability limit of Cu^2+^NO_*x*_^δ−^ is around 200 °C with direct restoration of the bare Cu^2+^ cations, while for Cu^2+^/8MR, the NO_*x*_^δ−^ complexes transform into the Cu^2+^NO species (Figure 1b). The latter is thermally stable up to ∼400 °C, at which a slight desorption peak is visible in the *p*_exp,NO_ line.

In the case of reduced Cu^+^, the beneficial metal-to-ligand charge transfer (see Section S3.1, Supporting Information) makes the resultant NO_3_^–^ anions a dominant species, stable in a wide temperature range (Figure 1c,d). Under the experimental conditions, nitrates should decay at ∼450 °C and ∼400 °C for the 6MR and 8MR sites, respectively. Nitrite anions decompose before nitrates (dashed purple line), and the corresponding stability limits are below 400 °C (6MR) and 350 °C (8MR). The mononitrosyl Cu^+^–NO species at the 6MR sites and the mono- and dinitrosyls at the 8MR sites decompose at even lower temperatures.

The theoretical stability limits of the most resistant Cu^2+^NO_3_^–^ adducts must be reconciled with the possible successive reactions that involve these intermediates in the NO–SCO reaction. Any mechanistic step occurring with the consumption of surface nitrates below their thermodynamic stability limit will influence this boundary. This behavior is examined for the six single Cu centers (Cu^2+^, Cu^+^, and Cu^2+^OH^–^ at the 6MR and 8MR sites) in various possible reactions with NO_*x*_, O_2_, and HONO species. The latter is instantly produced by the association of NO with the OH ligand (with an activation energy of only 0.25 eV ). The reactions considered are grouped according to their mechanistic features in Table 1 and are labeled with “**M**”, which refers to the monomeric (single) copper active centers.

**Table 1:** Collation of Conceivable Reactions of NO, O_2_, HONO, and H_2_O with Single Copper Centers Located in the 6MR and 8MR Sites of the CuSSZ-13 Zeolite and the Corresponding Reaction Energetics (Δ*E*_r_)

			Δ*E*_r_/eV	
reaction type	labeling	reaction equation	6MR	8MR
interaction of NO with copper hydroxyls	M.1.1	Cu^2+^OH^–^+ NO → (Cu^2+^OH^–^)NO	–0.96	–1.55
	M.1.2	(Cu^2+^OH^–^)NO → Cu^+^ + HONO	1.00	0.88
	M.1.3	Cu^2+^OH^–^ + NO → Cu^+^ + HONO	0.03	–0.66
interaction of HONO with copper centers	M.2.1	Cu^2+^ + HONO → Cu^+^ + H^+^ + NO_2_	0.21	–0.53
	M.2.2	Cu^2+^ + HONO → Cu^2+^NO_2_^–^ + H^+^	–1.01	–1.51
	M.2.3	Cu^+^ + H^+^ + HONO → Cu^2+^ + H_2_O + NO	0.68	1.42
interaction of NO/O_2_ with Cu^+^	M.3.1	Cu^+^ + NO → Cu^+^NO	–1.14	–1.19
	M.3.2	Cu^+^NO + O_2_ → Cu^2+^NO_3_^–^	–2.30	–1.90
	M.3.3	Cu^2+^NO_3_^–^ + NO → Cu^2+^NO_2_^–^ + NO_2_	–0.18	–0.29
	M.3.4	Cu^2+^NO_2_^–^ → Cu^+^ + NO_2_	1.22	0.97
interaction of NO/O_2_ with Cu^2+^	M.4.1	Cu^2+^ + NO → Cu^2+^NO	–1.12	–1.66
	M.4.2	Cu^2+^NO + O_2_ → Cu^2+^NO_3_^δ–^	–1.11	–1.08
	M.4.3	Cu^2+^NO_3_^δ–^ + NO → Cu^2+^NO_2_^δ–^ + NO_2_	–0.57	–0.61
	M.4.4	Cu^2+^NO_2_^δ–^ → Cu^2+^ + NO_2_	0.40	0.95
restoration of copper active centers	M.5.1	Cu^2+^ + H_2_O → Cu^2+^OH^–^ + H^+^	–0.71	–0.75
	M.5.2	Cu^2+^NO_2_^–^ + H_2_O → Cu^2+^OH^–^ + HONO	0.30	0.75
	M.5.3	2Cu^+^ + 2H^+^ + 1/2O_2_ → 2Cu^2+^ + H_2_O	–0.73	0.75

The reduced Cu^+^ transients, resulting from the reaction between the hydroxyl ligands and NO with the formation of HONO (**M.1.2–3,** see Table 1), are immediately converted into the corresponding nitrates (**M.3.1** and **M.3.2**). The latter, after subsequent interaction with NO (ligand comproportionation), can be transformed into nitrites (**M.3.3**), which by releasing the NO_2_ molecule restore the Cu^+^ active center (**M.3.4**) or partially hydrolyze, regenerating the Cu^2+^OH^–^ sites (**M.5.2**). Some of these reactions are partially reversible (small positive Δ*E*_r_ value) and are controlled by the entropic term of the gaseous products and the reaction temperature. Several anionic redox processes can also be distinguished, where the redox reaction occurs between the nitrogen-bearing ligands. At the same time, the divalent copper cation acts merely as a coordination site (**M.4.1–4**).

**Figure 2:**
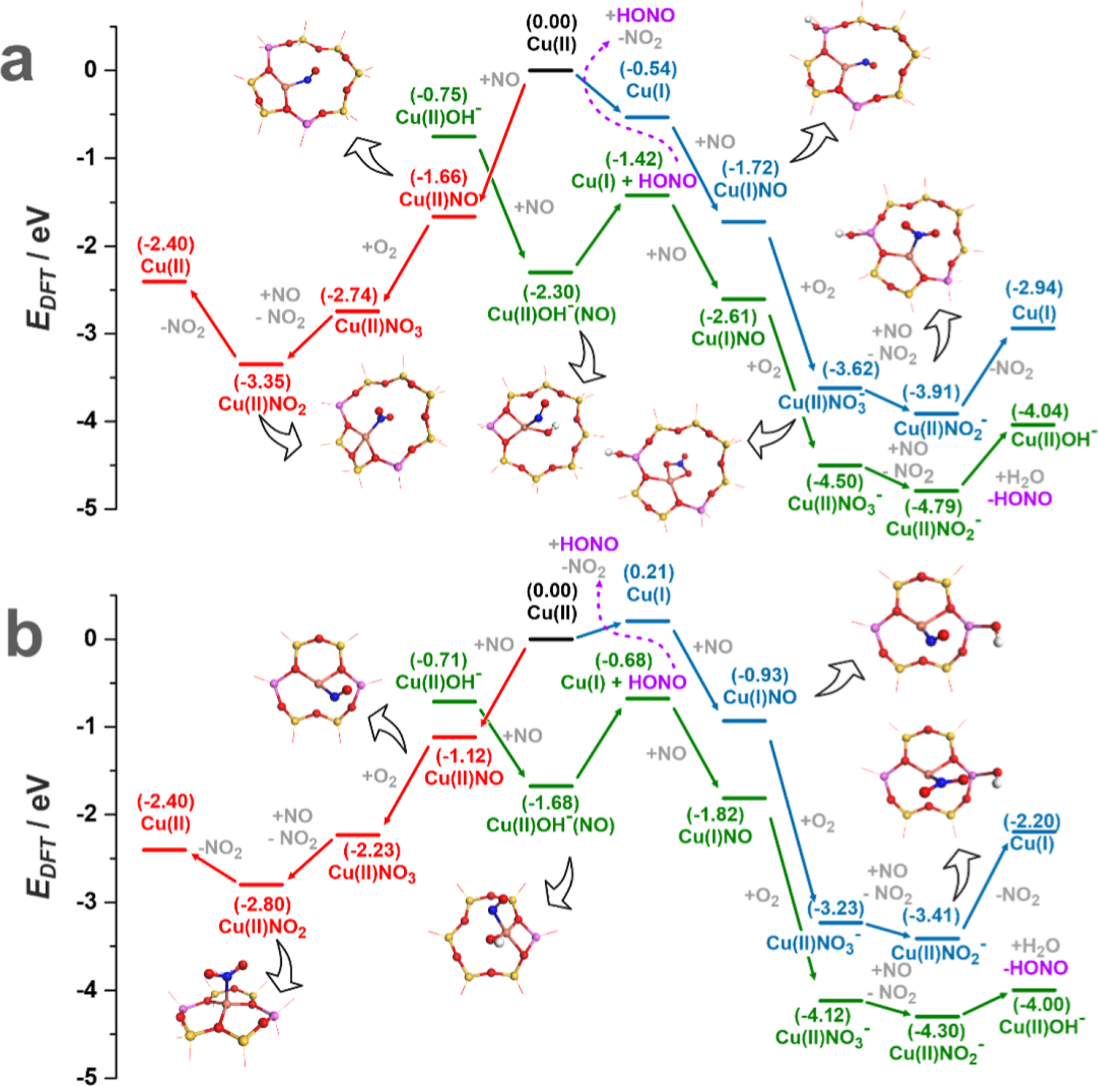
Energy profiles of the NO/O_2_ interaction with the Cu^2+^OH^–^ (green), Cu^2+^ (red), and Cu^+^ (blue) centers located in the 8MRs (a) and 6MRs (b). All values in brackets represent DFT energies (eV) relative to the bare Cu^2+^ centers.

Whereas the phase diagrams discussed above (Figure 1) unravel the (*p*, *T*) regions of the most stable adducts concerning the experimental conditions, Figure 2 shows a complex network of reactions involving the active Cu^2+^, Cu^2+^–OH^–^, and Cu^+^ centers that lead to the formation of the critical reaction intermediates summarized in Table 1. The prime mechanistic routes are highlighted with red, green, and blue colors. Two pathways of the cationic (metal) redox character (blue and green lines) lead to the same stable species (nitrates/nitrites). However, they differ in the nature of the initial active site: bare Cu^2+^ and hydroxylated Cu^2+^, respectively. Interestingly, anionic nitrate/nitrite ligands can be formed on the divalent copper centers due to their interaction with HONO directly (**M.2.2**) and indirectly (**M.2.1** and **M.3.1–3**). The HONO intermediate is produced preferentially along the green pathway (**M.1.3**) on the Cu^2+^OH^–^ sites in the 8MRs (−0.66 eV). Therefore, the progress of these reactions is sensitive to the temperature and the actual reactant composition, and the conditions of their occurrence are elucidated in Section 3.2.2. For the 6MR sites, this reaction is energetically slightly unfavorable (0.03 eV). Consumption of HONO along the blue pathway provides a requisite driving force for the green pathway, making HONO the versatile intermediate that links both reaction routes. Although in all cases the most stable is the bidentate Cu^2+^NO_3_^–^ (η^2^–O,O) adduct, it is not necessarily a terminal species of the reaction network investigated. The cage nitrates undergo further reactions upon interaction with the engaged NO, revealed by the corresponding thermodynamic reaction profile (see below), illustrating their dynamic character under the reaction conditions.

The reaction pathways of the ligands (red lines) are characterized by weaker energetic effects compared to the cationic redox and lead to the formation of NO, NO_2_^δ−^, and NO_3_^δ−^ adspecies. The NO_2_ molecule is then more easily removed from the 6MR copper sites (Δ*E*_r_ = 0.40 eV) than from Cu^2+^/8MR, for which the desorption energy is higher and equals 0.95 eV (**M.4.4**).

The results show that due to the versatile redox behavior of the volatile HONO, the Cu^2+^OH^–^ species may cooperate through space with the Cu^2+^/8MR centers, making the latter redox-active toward NO/O_2_ before their reduction. Along with the cationic (metal) redox pathways, this contributes to the mechanistic network of the NO–SCO reaction, featuring the formation of anionic NO_*x*_^–^ species.

#### 3.2.2 Thermodynamic Reaction Profiles for the Isolated Cu Centers

**Figure 3:**
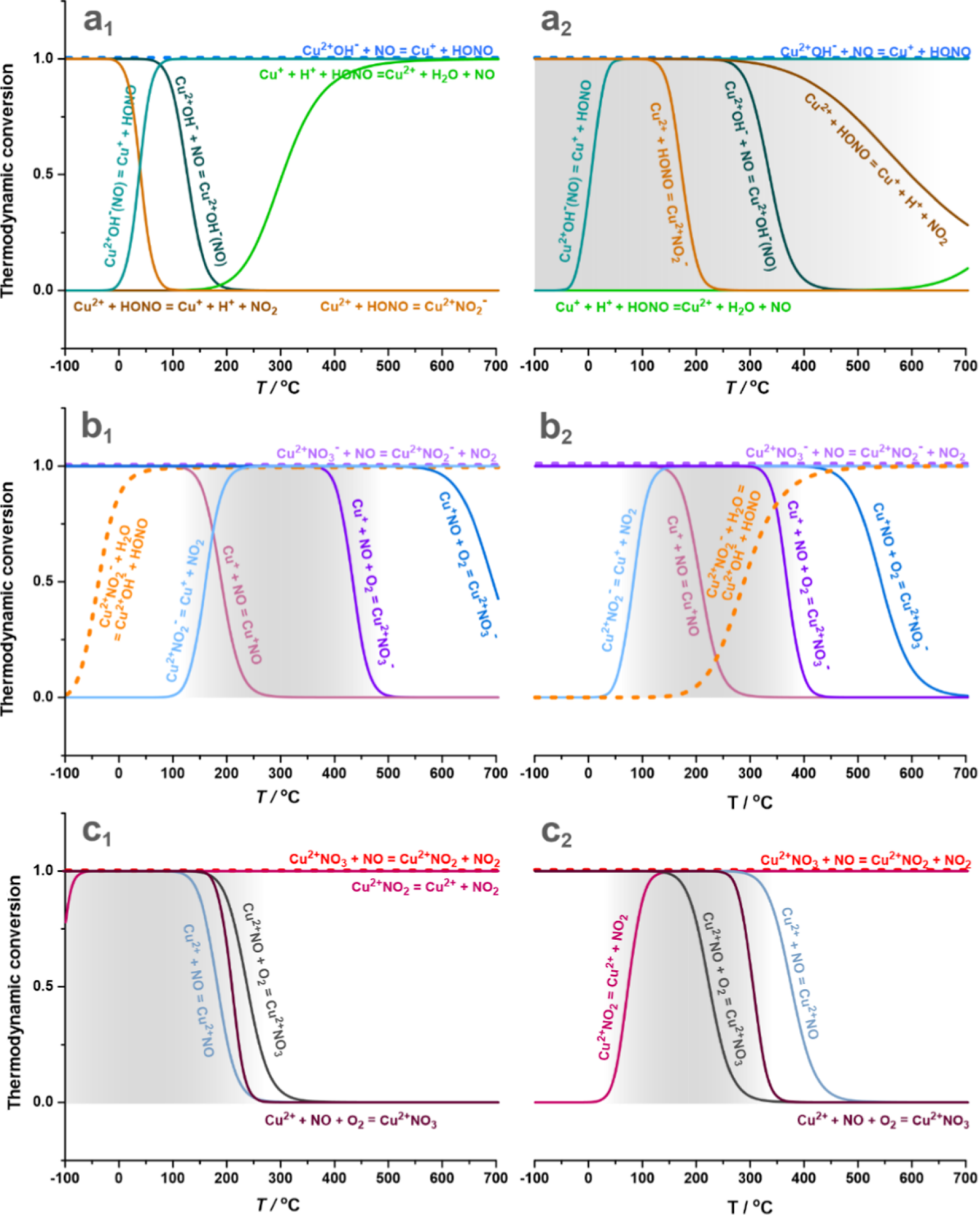
Thermodynamic ξ(*T*) profiles for the NO oxidation reaction (NO_2_ formation) on the isolated copper species involving the HONO pathway (a_1_,a_2_), the cationic metal-redox pathway (b_1_,b_2_), and the anionic ligand-redox pathway (c_1_,c_2_). Subscript 1 (left panels) denotes the results for the 6MR and subscript 2 (right panels) for the 8MR sites. The areas highlighted in gray indicate the thermodynamic windows that are favorable for NO_2_ production. The parameters set used for the calculations: *p*_NO_*/p*^0^ = *p*_NO_2__/*p*^0^ = 5 × 10^–4^, *p*_O_2__/*p*^0^ = 4.5%, *p*_H_2_O_/*p*^0^ = 3%, *p*_HONO_/*p*^0^ = 10^–6^.

Thermodynamic reaction profiles (Figure 3) were calculated to visualize the dynamic equilibria of the reactions collated in Table 1, concerning their temperature responses. In such an approach, the 0–1 scale of the profiles is used to indicate the direction and extent of a particular reaction. Please note that the value 1 corresponds to the forward reaction, while 0 corresponds to the backward reaction, concerning the equations in Table 1. For instance, ξ = 0 indicates that the equilibrium is entirely shifted toward (NO)Cu^2+^OH^–^ for the reaction (NO)Cu^2+^OH^–^ = Cu^+^ + HONO. In contrast, for ξ = 1, it is wholly shifted toward the dissociation products Cu^+^ + HONO (see the cyan line in Figure 3a_1_). For the calculations of the ξ(*T*) profiles, we used *p*_NO_/*p*^0^ = *p*_NO_2__/*p*^0^ = 5 × 10^–4^ (500 ppm), corresponding to the experimental conditions of ∼100% progress in the NO–SCO reaction (Section S3.4). The necessary Δ*G*_r_ values were obtained using the reaction energies, Δ*E*_r_, collected in Table 1, complemented by the entropic terms calculated within the ideal gas approximation. As a result of its elusive nature, it is impossible to experimentally assess the partial pressure of HONO during the reaction. Still, its level is distinctly lower than that of the NO in the feed. Therefore, in auxiliary calculations, the influence of the partial pressure of HONO on the thermodynamic ζ(*T*) profiles was examined (Section S5, Figure S11, Supporting Information), and a reasonable estimate of *p*_HONO_/*p*^0^ = 10^–6^ was applied for further modeling. Next, using the set of reactions listed in Table 1, the course of the reactions contributing to the NO–SCO processes (evolution of NO_2_) was explored. The experimental constraints provided by the catalytic TPSR studies of oxidation of NO into NO_2_ were used to justify the results obtained by the applied modeling.

Analysis of the NO oxidation cycles allowed us to distinguish three main routes of NO_2(g)_ formation, which include (1) a Cu^2+^–OH^–^/HONO/NO_*x*_ pathway (Figure 3a_1,2_), (2) a cationic metal-redox Cu^+^/NO_*x*_/O_2_ pathway (Figure 3b_1,2_), and (3) an anionic ligand-redox Cu^2+^/NO_*x*_/O_2_ pathway (Figure 3c_1,2_).

The calculated ξ(*T*) profiles allow for the straightforward establishment of the temperature windows in which NO_2_ is catalytically produced along the specific reaction routes, accounting for the NO–SCO and fast-SCR reaction. These temperature windows are highlighted in gray in Figure 3, and the associated reactions are discussed in more detail below.

##### 3.2.2.1 HONO Pathway

Hydroxyl copper(II) centers react readily with NO, forming HONO intermediates.^13,55^ Although mononitrosyl (NO)Cu^2+^OH^–^ adducts (**M.1.1**) are stable below 100 and 300 °C for the 6MR and 8MR sites, respectively (Figure 3a_1,2_, dark green line), subsequent formation of HONO (**M.1.2**) drives the reaction toward copper reduction. Thus, the overall process (**M.1.3**) is thermodynamically favorable in the wide range of the experimental temperatures of the NO–SCO reactions for both copper sites. As a result, depending on the actual reaction conditions, the versatile redox HONO intermediate can act as a reduction agent for Cu^2+^ with the electroprotic formation of NO_2_ (**M.2.1**), a source of nitrites upon deprotonation **(M.2.2**) or as an oxidant of Cu^+^ with the formation of H_2_O and NO (**M.2.3**). The reactivity of HONO with copper cations is highly sensitive to their location in the SSZ-13 framework. In particular, for the 6MR centers, copper reduction is not expected to occur (Figure 3a_**1**_, brown line), in contrast to the Cu^2+^/8MR. An opposite behavior was found for the unproductive Cu^+^ oxidation (bright green line), which is favorable above 200 °C for the 6MR sites but is prohibited in the experimental temperature range for 8MR. As a result, 6MR centers are not expected to participate in the metal redox pathway of the NO oxidation reaction, in contrast to Cu^2+^/8MR cations, which can only be reduced by the HONO intermediate (**M.2.1**).

##### 3.2.2.2 Metal-Redox Pathway

Prior reduction of copper conditions the progress of the cationic metal redox pathway. It is realized in the reduction half-cycle for both 6MR and 8MR sites or by interaction with HONO (8MR) produced previously on the Cu^2+^–OH^–^ centers (NO–SCO). At first, the isolated Cu^+^ cations in the presence of NO form paramagnetic Cu^+^-NO adducts (**M.3.1**), which react quickly with O_2_ (**M.3.2**) with the formation of highly stable copper nitrates (Cu^2+^NO_3_^–^). For the 6MR and 8MR sites, NO adsorption is thermodynamically preferred below 200 °C (Figure 3b_1,2_, dark pink line), but NO_3_^–^ production is favorable up to 600 and 500 °C for 6MR and 8MR, respectively (blue line). However, despite their thermodynamic feasibility for both 6MR and 8MR sites, the nitrates can be produced on the Cu^2+^/8MR centers only since their previous reduction by HONO is required (for Cu^2+^/6MR, this process is thermodynamically not favorable). In the presence of NO, nitrates can undergo comproportionation into nitrites with NO_2_ release (**M.3.3**), as shown by the purple dashed line in Figure 3b_1,2_, and this process is favored throughout the whole temperature window. Copper nitrites (Cu^2+^NO_2_^–^) can then discharge NO_2_ with concomitant reduction of Cu^2+^ to Cu^+^ (**M.3.4**), formally closing the metal-redox pathway (Figure 2, blue lines). The highlighted areas in Figure 3b_1,2_ result from the thermodynamically allowed stoichiometric comproportionation reaction of nitrates with the encaged NO. This reaction stops when the nitrates are entirely transformed into nitrites. Therefore, the decomposition border of the latter (pale blue line) controls the low-temperature limit of a sustainable evolution of NO_2_. Water affects the metal-redox pathway by hydrolyzing the nitrites. The restored Cu^2+^(OH)^−^ and HONO species are driving, thereby, the HONO pathway (Figure 3a_1,2_). At the same time, the metal-redox pathway is terminated (all reduced Cu^+^ are transformed to Cu^2+^ or Cu^2+^(OH)^−^) in the entire temperature range for 6MR above 0 °C, while for 8MR, this effect starts at 200 °C.

##### 3.2.2.3 Ligand-Redox Pathway

For a comprehensive account, the interaction of Cu^2+^ with NO + O_2_ was also examined in an analogous way to the Cu^+^ pathway of the formation of nitrates (**M.3.2**). The development of the Cu^2+^NO adduct (**M.4.1**) and its subsequent oxidation to Cu^2+^NO_3_^δ–^ in the presence of oxygen (**M.4.2**) are exothermic processes for both 6MR and 8MR sites (Table 1). The resulting Cu^2+^NO_3_^δ–^ species are less stable than the corresponding nitrates and can exist below 250 °C for the 6MR and 8MR sites (Figure 3c_1,2_, dark gray line). The ligand comproportionation between NO_3_^δ–^ and NO leads to the formation of NO_2(g)_ (**M.4.3**). Such reaction is allowed in the entire temperature range examined (red and reddish-purple lines) for 6MR and above 50 °C for 8MR. The Cu^2+^OH^–^ centers can be regenerated by hydrolysis of the captured water molecule (**M.5.1**) and less efficiently by the reaction of copper nitrite with water (**M.5.2**).

**Figure 4:**
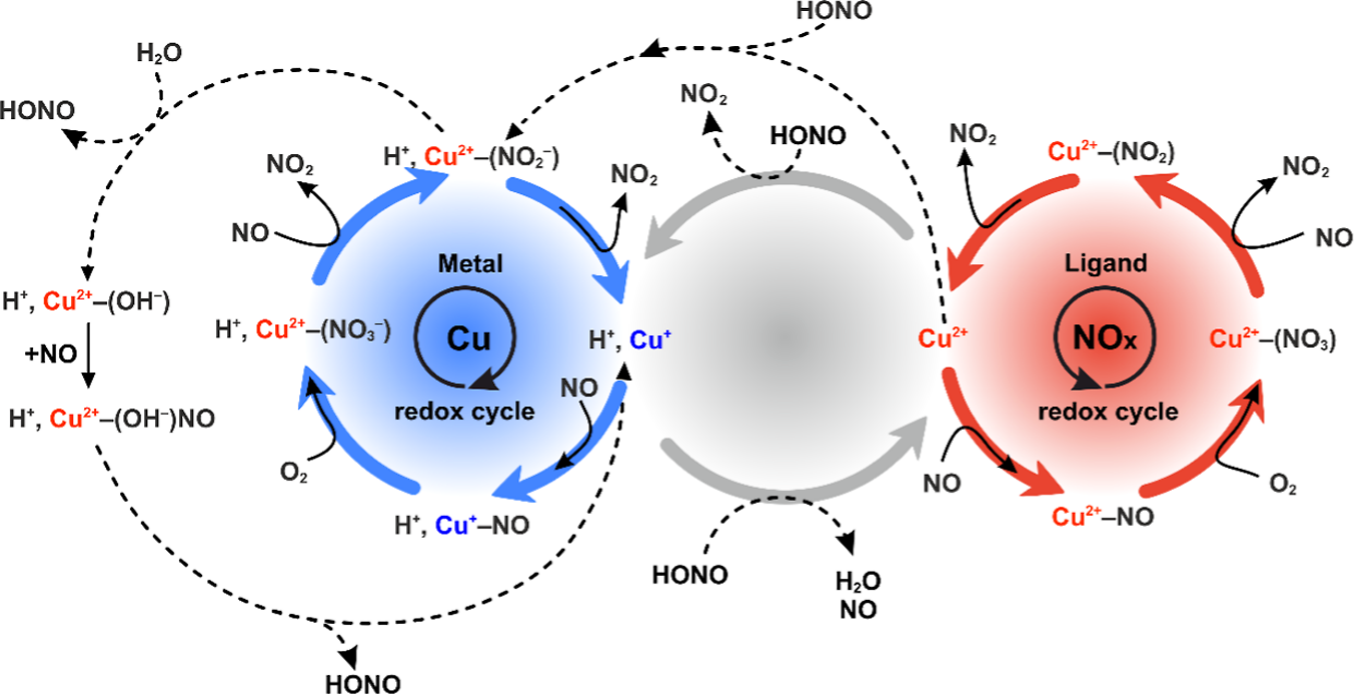
Mechanistic pathways of NO/O_2_ interaction with the isolated Cu^2+^ and Cu^2+^–OH^–^ centers in the CuSSZ-13 zeolite. Copper oxidation states are indicated in blue for the Cu^+^ and red for Cu^2+^cations, whereas gray features represent the redox of HONO.

The entire network of reactions discussed above is summarized in Figure 4. It provides the mechanistic and thermodynamic interpretation of the role of single copper centers in the reaction network of NO with O_2_, which constitutes the NO–SCO process. A particular distinction was made to separate the cationic copper redox cycle (blue ring), the anionic ligand redox cycle (red ring), and the reactions of the HONO intermediate (central ring), which connects both redox cycles.

#### 3.2.3 Dimeric Cu Species

Based on the preliminary screening, a set of thermodynamically feasible reactions was proposed for the NO/O_2_ interaction with dimeric copper sites (Table 2). As in the case of single sites, they were grouped according to the mechanistic relevance and labeled with “**D**” to spot the dimeric (dual) nature of the involved copper active centers.

**Table 2:** Collation of Conceivable Reactions Taken into Account for the Analysis of the NO/O_2_ Interaction with the Dimeric Copper Centers of the CuSSZ-13 Zeolite and the Corresponding Reaction Energetics (Δ*E*_r_)

reaction type	labeling	reaction equation	Δ*E*_r_/eV
interaction of NO/O_2_ with reduced Cu^+^∪Cu^+^ centers	D.1.1	Cu^+^∪Cu^+^ + O_2_ → Cu^2+^–O_2_^2–^–Cu^2+^	–2.08
	D.1.2	Cu^+^∪Cu^+^ + NO → Cu^+^–(NO)–Cu^+^	–2.49
	D.1.3	Cu^+^–(NO)–Cu^+^ + O_2_ → Cu^2+^–(NO_3_^–^)–Cu^+^	–0.44
interaction of NO/O_2_ with bridging-oxo Cu^2+^–O^2–^–Cu^2+^ centers	D.2.1	Cu^2+^–O^2–^–Cu^2+^ + NO → Cu^2+^–(NO_2_^–^)–Cu^+^	–1.42
	D.2.2	Cu^2+^–(NO_2_^–^)–Cu^+^ + O_2_ → Cu^2+^–(NO_3_^–^,O^–^)–Cu^2+^	–0.81
	D.2.3	Cu^2+^–(NO_3_^–^,O^–^)–Cu^2+^ + NO → Cu^2+^–(NO_2_^–^,NO_3_^–^)–Cu^2+^	–1.11
interaction of NO/O_2_ with bridging-peroxo Cu^2+^–O_2_^2–^–Cu^2+^ centers	D.3.1	Cu^2+^–(O_2_^2–^)–Cu^2+^ + NO → Cu^2+^–(NO_3_^–^)–Cu^+^	–0.84
	D.3.2	Cu^2+^–(NO_3_^–^)–Cu^+^ + NO → Cu^2+^–(NO_2_^–^)_2_–Cu^2+^	–1.65
	D.3.3	Cu^2+^–(NO_2_^–^)_2_–Cu^2+^ + O_2_ → Cu^2+^–(NO_3_^–^)_2_–Cu^2+^	–1.77
NO_2_ release	D.4.1	Cu^2+^–(NO_2_^–^,NO_3_^–^)–Cu^2+^ → NO_2_ + Cu^2+^–(NO_2_^–^,O^–^)–Cu^2+^	0.49
	D.4.2	Cu^2+^–(NO_2_^–^,O^–^)–Cu^2+^ → NO_2_ + Cu^2+^–O^2–^–Cu^2+^	0.55
	D.4.3	Cu^2+^–(NO_3_^–^,O^–^)–Cu^2+^ → NO_2_ + Cu^2+^–O_2_^2–^–Cu^2+^	0.75
	D.4.4	Cu^2+^–(NO_3_^–^)_2_–Cu^2+^ → NO_2_ + Cu^2+^–(NO_3_^–^,O^–^)–Cu^2+^	1.09
	D.4.5	Cu^2+^–(NO_2_^–^)–Cu^+^ → NO_2_ + Cu^+^∪Cu^+^	2.02
NO to NO_2_ oxidation	D.5.1	Cu^2+^–(NO_3_^–^)_2_–Cu^2+^ + NO → NO_2_ + Cu^2+^–(NO_2_^–^,NO_3_^–^)–Cu^2+^	0.31
	D.5.2	Cu^2+^–(NO_2_^–^,NO_3_^–^)–Cu^2+^+ NO → NO_2_ + Cu^2+^–(NO_2_^–^)_2_–Cu^2+^	–0.94
	D.5.3	Cu^2+^–(NO_2_^–^)–Cu^+^ + NO → NO_2_ + Cu^+^–(NO)–Cu^+^	–0.46
	D.5.4	Cu^2+^–O_2_^2–^–Cu^2+^ + NO → Cu^2+^–O^2–^–Cu^2+^ + NO_2_	–0.92

**Figure 5:**
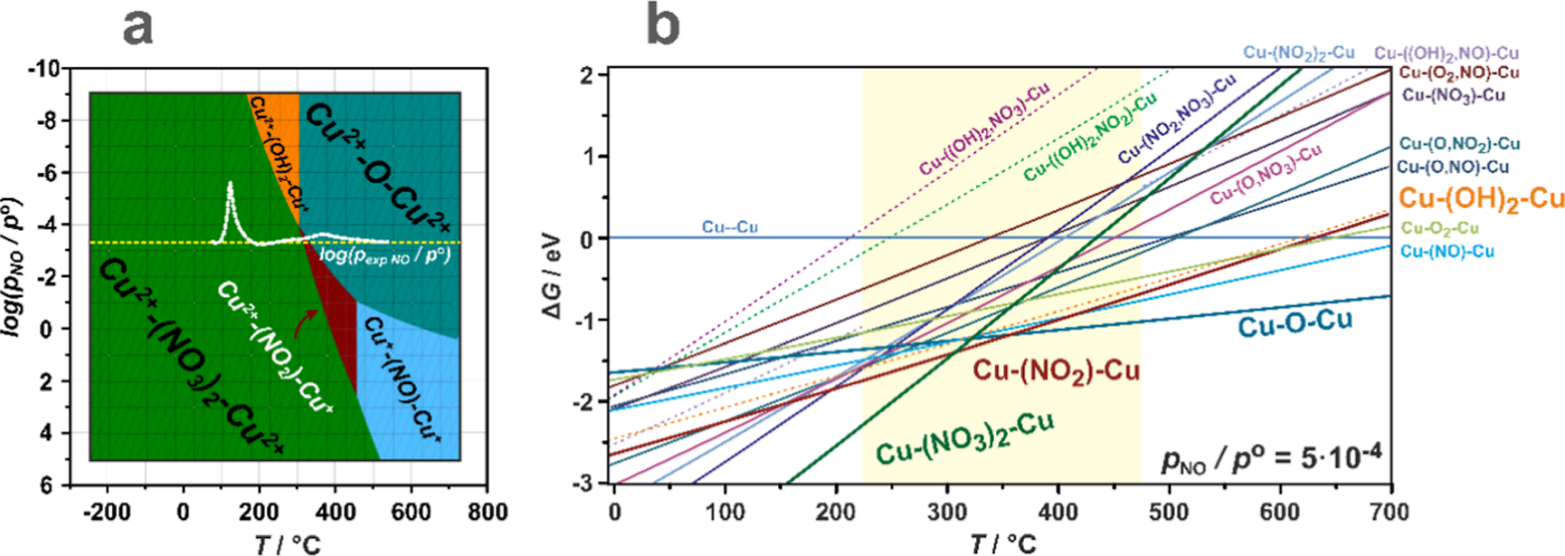
Thermodynamic Δ*G*(*p,T*) diagram for dual copper centers interacting with O_2_, NO, and H_2_O as a function of temperature and NO pressure, for *p*_O_2__/*p*^0^ = 4.5% and *p*_H_2_O_/*p*^0^ = 3% kept constant (a). Pressure cut (yellow dotted line) of the Δ*G*(*p,T*) diagram at *p*_NO_ = 5 × 10^–4^ (b). The dotted white line corresponds to experimental changes in the NO pressure during the NO–SCO reaction.

Five generic types of reactions at the dimeric active centers were distinguished and classified into the interaction of NO and O_2_ with the reduced dual copper sites (**D.1.1–3**), bridging copper-oxo (**D.2.1–3**), copper-peroxy (**D.3.1–3**) centers, reactions associated with direct NO_2_ release (**D.4.1–5**), and oxidation of NO to NO_2_ (**D.5.1–4**). The energies collected in Table 2, complemented by the results shown in Table S8 and Table S10, allowed the construction of the corresponding Δ*G*(*p*_NO_, *T*) diagram, setting *p*_O_2__*/p*^0^ = 4.5% and *p*_H_2_O_*/p*^0^ = 3%, which illustrate the complex behavior of these centers upon contact with the gas mixture of O_2_/NO/H_2_O (Figure 5).

A bottom envelope of the thermodynamic diagram (Figure 5a) shows the most stable species in the considered ranges of *T* and *p*_NO_. The adsorbed dinitrate Cu^2+^–(NO_3_^–^)_2_–Cu^2+^ species dominate the low-temperature region over the entire pressure range. An increase in temperature leads to the formation of bridging Cu^2+^–(OH^–^)_2_–Cu^2+^ centers or adsorbed species Cu^2+^–(NO_2_^–^)–Cu^2+^, depending on the NO pressure. The first process takes place at *p*_NO_ < 10^–3^ atm and can be treated as an exchange of the NO_3_^–^ ligand with OH^–^ derived from the dissociative H_2_O attachment (Cu^2+^–(NO_3_^–^)_2_–Cu^2+^ + H_2_O_(g)_ → Cu^2+^–(OH^–^)_2_–Cu^2+^ + 2NO_(g)_ + 3/2O_2(g)_). Due to its multispecies nature, this reaction is likely to be kinetically restricted and can proceed more quickly when the liberation of the NO_2_ molecules is considered (see below). Moving to higher temperatures, dihydroxyl-copper centers tend to release water molecules (starting at *T* ∼ 300 °C in a wide range of *p*_NO_), converting into Cu^2+^–O^2–^–Cu^2+^ bridging entities (Cu^2+^–(OH^–^)_2_–Cu^2+^ → Cu^2+^–O^2–^–Cu^2+^ + H_2_O, Δ*E*_r_ = 1.16 eV), which is driven by an increase in the entropy due to water liberation. The transformation observed at higher NO pressures (*p*_NO_ > 10^–3^ atm) corresponds to the decomposition of nitrates with a release of O_2_ and NO molecules (Cu^2+^–(NO_3_^–^)_2_–Cu^2+^ → Cu^2+^–(NO_2_^–^)–Cu^+^ + NO_(g)_ + 3/2O_2(g)_). An increasing temperature makes the resulting nitrite species prone to detach NO and restore the Cu^2+^–O^2–^–Cu^2+^ centers (reverse reaction **D.2.1**).

Since the bottom envelope of the 2D diagram does not reveal the less stable species, its cut for the selected NO pressure of *p*_NO_ = 5 × 10^–4^ atm (dashed yellow line corresponding to the experimental value in Figure 5a) is shown in Figure 5b. The stability lines show that the coadsorption of water and NO (purple, green, and violet dashed lines) is thermodynamically unfavored due to the lateral repulsion between the NO and H_2_O ligands. As expected, the complexes resulting from separate binding of NO and O_2_ (Cu^+^–(O_2_,NO)–Cu^+^, Cu^+^–(O,NO)–Cu^+^, Cu^+^–(O,NO_2_)–Cu^+^) are unstable regarding the nitrates and nitrites, which are thermodynamically dominant until 350 °C, then converting into the Cu^2+^–O^2–^–Cu^2+^ centers. The latter may decompose into Cu^+^∪Cu^+^ upon oxygen discharge under strongly lean-oxygen and high-temperature conditions only (present, e.g., during thermal activation of the samples under a vacuum; see Figure S8). In particular, in the NO–SCO/SCR temperature window (area highlighted in yellow in Figure 5b), several species characterized by similar stability can coexist and be mutually interconverted. This promotes the versatile reactivity of the bridging copper centers, which can be involved in the stoichiometric regularization of nitrates^9^ during the OHC stage of NH_3_–SCR and in NO–SCO reactions. In particular, the identified NO_2_ production pathways initiate the fast SCR process above 200 °C.^12,33^ The experimentally observed NO_2_ may be produced following (1) the direct release pathways (**D.4.1–5**) or by (2) the comproportionation reaction of nitrates with gaseous NO (**D.5.1–3**), described below in more detail.

##### 3.2.3.1 Pathways of Direct NO_2_ Release

**Figure 6:**
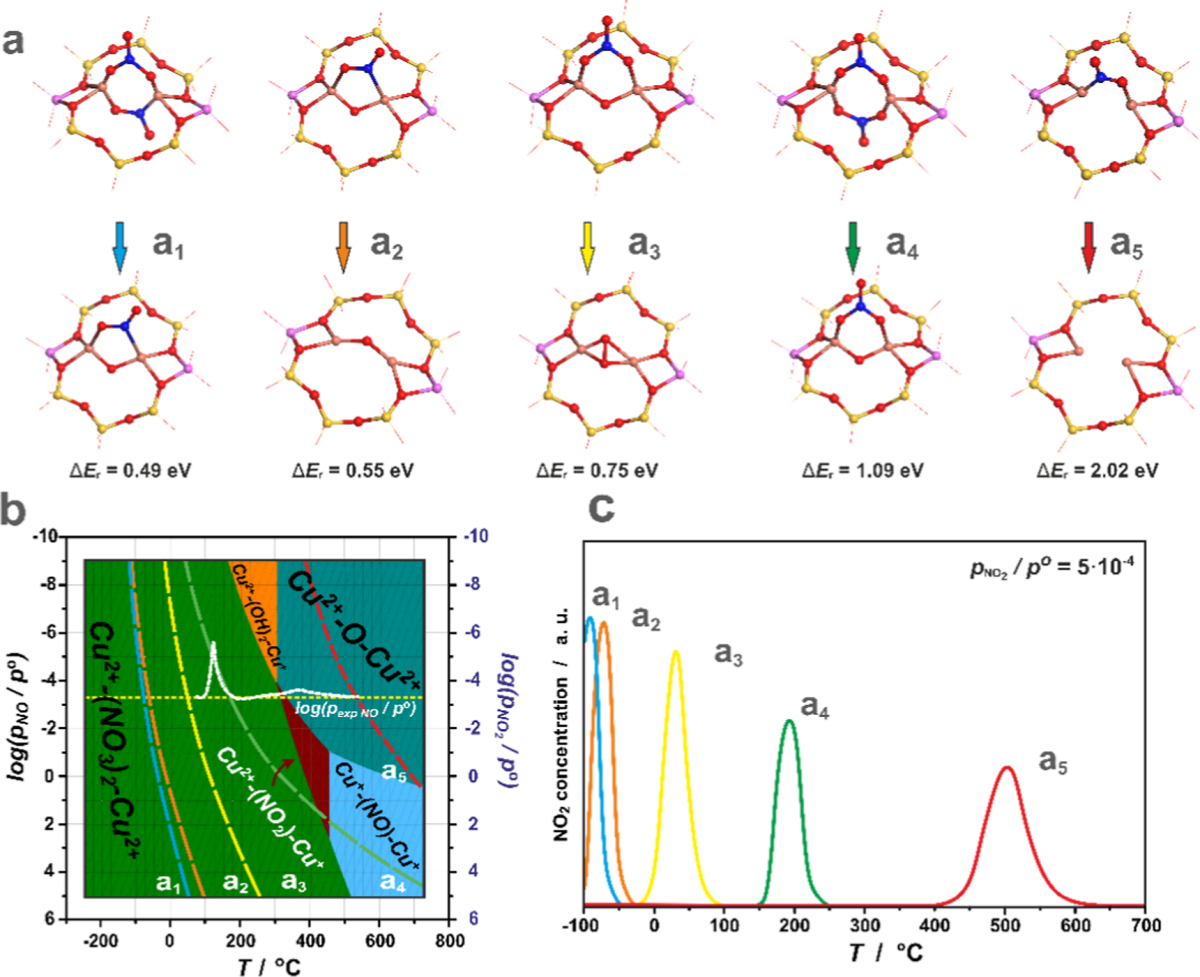
Possible processes of direct evolution of NO_2_ from Cu–(NO_*x*_^–^)–Cu adducts (a_1_–a_5_). The NO_2_ release limits (dotted lines with colors corresponding to the reactions a_1_–a_5_), superimposed on the bottom envelope of the thermodynamic stability diagram (b), together with the corresponding simulated NO_2_ desorption profiles (c) for *p*_NO_2__ set to 5 × 10^–4^ atm.

Possible mechanistic routes of direct NO_2_ release were selected after a detailed analysis of the decomposition reactions of the nitrate/nitrite adspecies collated in Table 2, reactions **D.4.1–5**. The corresponding structures with their energetics are shown in Figure 6a_1_–a_5_. Thermodynamic regions of the evolution of nitrogen dioxide were next assessed by FPT modeling. The results are shown in Figure 6b, where the NO_2_ release profiles as a function of *p*_NO_2__ (dashed lines) are superimposed on the corresponding phase diagrams. For reactions **D.4.1–4**, NO_2_ desorption occurs at temperatures lower than the thermal stability border of the dominant Cu^2+^–(NO_3_^–^)_2_–Cu^2+^ species (Figure 6a_1_**–**a_4_). Reaction **D.4.5** is expected to occur at temperatures that exceed the thermal stability of the nitrates and NO_2_ (Figure 6a_**5**_) and is actually replaced by NO release with the restoration of the oxo-bridge (250 °C for *p*_NO_ = 5 × 10^–4^ atm). The most significant seems to be reaction **D.4.4,** which involves the most stable Cu^2+^–(NO_3_^–^)_2_–Cu^2+^ adduct since NO_2_ release (Figure 6a_4_) occurs in the temperature window observed in the SCR^12^ and NO–SCO reactions (see below).

The corresponding NO_2_ desorption profiles were then modeled by calculating dΘ/d*T* vs *T* dependence^40^ for *p*_NO_2__ set to 5 × 10^–4^ atm (Figure 6c). The results indicate that the evolution of NO_2_ according to reactions **D.5.1** and **D.5.2** can already occur at ∼0 °C. In contrast, for reactions **D.5.3–5**, the desorption peaks are expected at ∼100, ∼210, and 500 °C, respectively.

##### 3.2.3.2 Formation of NO_2_ via Comproportionation Reaction

**Figure 7:**
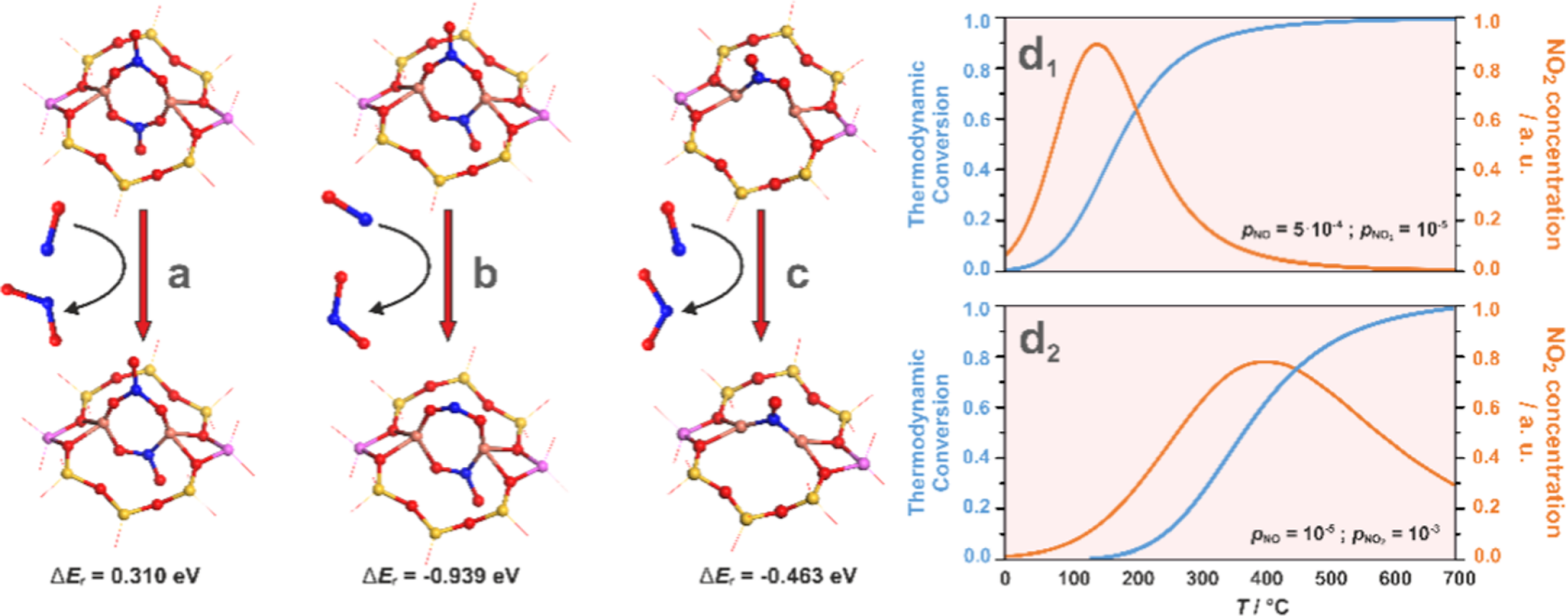
Interaction pathways of NO_(g)_ with NO_*x*_^–^ adducts on the dual copper centers leading to the formation of NO_2_ (a–c). Thermodynamic reaction profiles (blue lines) and their derivatives (orange lines) were calculated for reaction “a” with the *p*_NO_/*p*_NO_2__ values corresponding to the initial (d_1_) and final (d_2_) conditions of the NO–SCO reaction. Reactions “b” and “c” running already at negative temperatures were not further analyzed.

Nitrogen dioxide may also be produced along the comproportionation reaction of nitrates with the encaged NO, as implied by reactions **D.5.1–3** (Table 2). The structure of the involved adspecies and the corresponding reaction energies are presented in Figure 7a–c.

Among those reactions, only the first process is characterized by the positive reaction energy; therefore, it can be effectively controlled by the temperature and pressure of the reactants. Its temperature dependence is shown in Figure 7d_1_,d_2_, for the composition of the NO/NO_2_ mixtures corresponding to the initial and final stages of the NO oxidation reaction, respectively (see below). In the first case (Figure 7d_**1**_), the inflection point, where Δ*G*_r_ turns negative, appears at *T* = 110 °C and is associated with the release of NO_2_ due to reaction **D.5.1**. At conditions closer to the end of NO oxidation (Figure 7d_2_), this peak broadens and is shifted toward higher temperatures (∼400 °C). Reaction **D.5.1** involves the most stable Cu^2+^–(NO_3_^–^)_2_–Cu^2+^ species (see Figure S10e) and may constitute an essential channel of the overall NO oxidation process. The other two reactions (**D.5.2–3**) are exoenergetic, so they can spontaneously occur at various temperatures and pressures. However, the parent reactants of these reactions are thermodynamically less stable than the Cu^2+^–(NO_3_^–^)_2_–Cu^2+^ species, which makes them less effective in the NO_2_ production.

#### 3.2.4 Interaction of HONO with Dual Copper Sites

**Figure 8:**
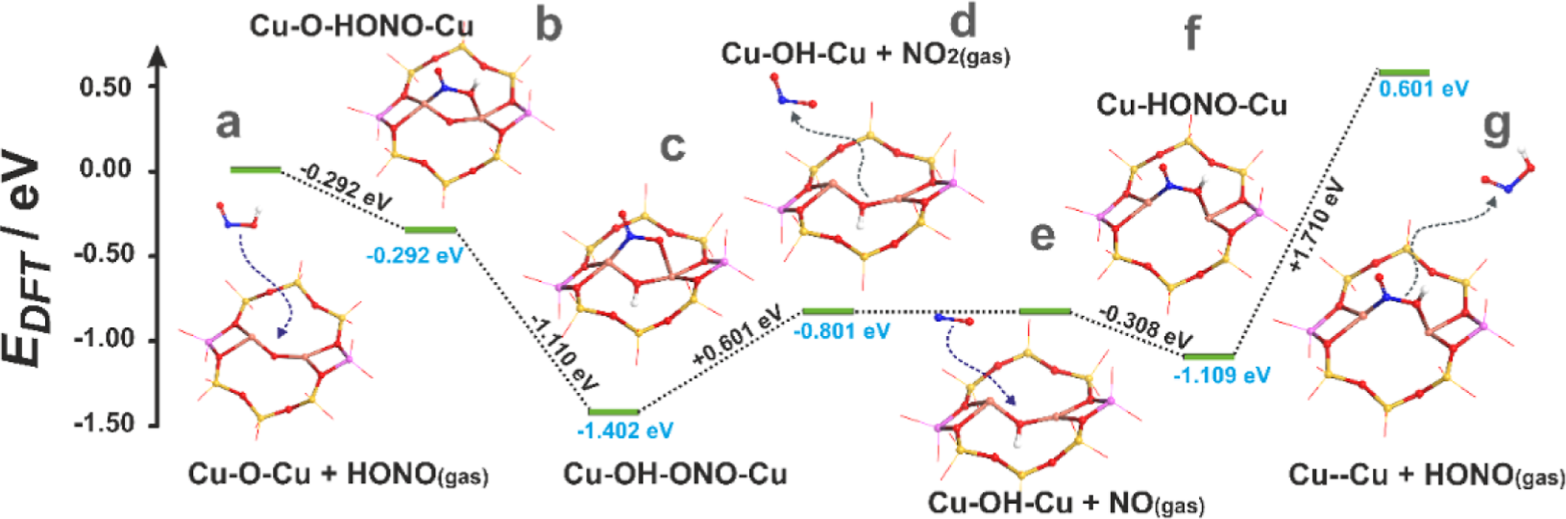
Energy profile for the HONO interactions with the dual copper centers in CuSSZ-13 zeolite.

A conceivable set of reactions underlying the complex interaction of HONO intermediates with the dual Cu centers (labeled “**DH**”) is shown in Table 3, and the associated structures with their energies are shown in Figure 8.

**Table 3:** Conceivable Reaction Set Involving the Interaction of HONO Intermediates with the Dual Copper Centers and the Corresponding Reaction Energetics Δ*E*_r_

reaction type	labeling	reaction equation	Δ*E*_r_/eV
interaction of HONO with dual Cu centers	DH.1	Cu^2+^–O^2–^–Cu^2+^ + HONO → Cu^2+^–(HONO)O^2–^–Cu^2+^	–0.29
	DH.2	Cu^2+^–(HONO)O^2–^–Cu^2+^ → Cu^2+^–(NO_2_^–^)OH^–^–Cu^2+^	–1.11
	DH.3	Cu^2+^–(NO_2_^–^)OH^–^–Cu^2+^ → Cu^2+^–OH^–^–Cu^+^ + NO_2_	0.60
	DH.4	Cu^2+^–OH^–^–Cu^+^ + NO → Cu^+^–(HONO)–Cu^+^	–0.31
	DH.5	Cu^+^–(HONO)–Cu^+^ → Cu^+^∪Cu^+^ + HONO	1.71
	DH.6	Cu^+^∪Cu^+^ + 1/2O_2_ → Cu^2+^–O^2–^–Cu^2+^	–1.81

For further discussion, it was assumed that the HONO species produced by the NO reaction with Cu^2+^OH^–^ (see Table 1) can be transported within the zeolite channels (Figure 8a) and then associatively captured by the dimeric (Cu^2+^–O^2–^–Cu^2+^) centers in a bidentate fashion (Figure 8b). The overall energy of the resultant reaction, **DH.1,** equals −0.29 eV. In the next stage, HONO dissociation (O–H bond breaking) is assumed, leading to the formation of a hydroxyl bridge together with a NO_2_^–^ adspecies (**DH.2**) and the stabilization energy of −1.11 eV (Figure 8c). The detachment of the NO_2_ molecule (**DH.3**) is an uphill process with an energy cost of 0.60 eV (Figure 8d). The resulting copper-hydroxyl center interacts with a NO_(gas)_ molecule to form a Cu^+^–HONO–Cu^+^ adduct (Figure 8e,f and reaction **DH.4**). After discharging of HONO (Figure 8g, **DH.5**), the resulting Cu^+^∪Cu^+^ center can then be reoxidized, restoring the initial Cu^2+^–O^2–^–Cu^2+^ centers (**DH.6**).

**Figure 9:**
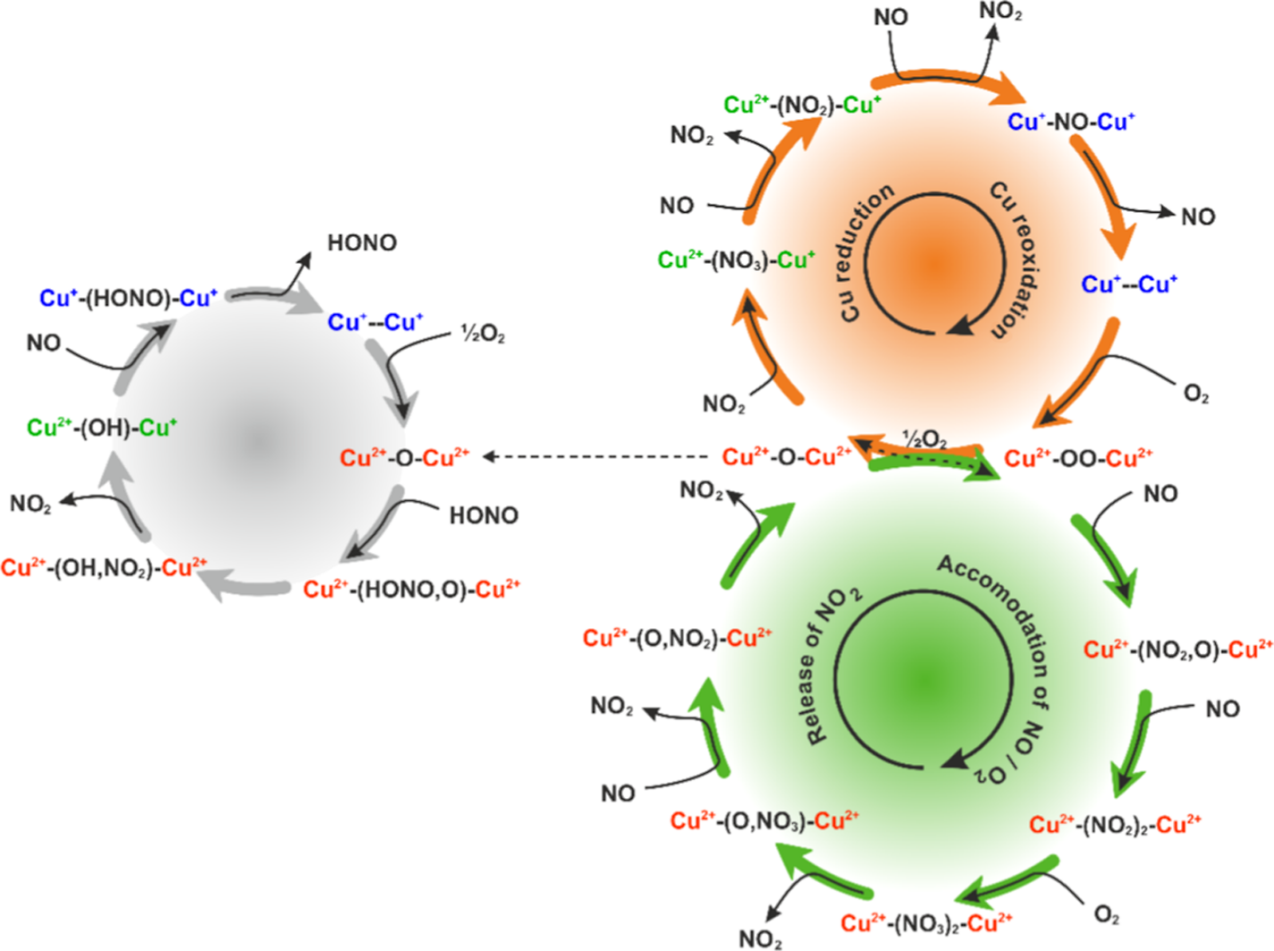
Reaction cycles involving the nitrate, nitrite, and HONO species adsorbed on the dual copper sites of the CuSSZ-13-zeolite. Copper oxidation states are indicated in blue for Cu^+^, red for Cu^2+^ cations, and green for mixed Cu^2+^/Cu^+^.

The overall activity of the dual copper centers can be recapitulated in the form of overlapping reaction cycles (Figure 9), which illustrate the complex dynamics of the nitrate, nitrite, HONO, and hydroxyl species in contact with the gas mixture of O_2_, NO, and NO_2_. The actual thermodynamic conditions (*T*, *p*_NO_) control the occurrence of the particular reactions.

The most essential characteristic distinguishing dual from single copper centers is their ability to accommodate oxygen in the form of bridging anionic peroxo (O_2_^2–^) and oxo (O^2–^) species. Since both Cu cations are oxidized, the redox activity of such centers acquires an anionic character (ligand redox), and electrons needed for the formation of nitrates and nitrites upon interaction with NO/O_2_ are provided directly by insertion of the bridging oxygen anion. Release of the NO_2_ molecules occurs typically with preservation of the bridging oxygen moiety without changing the oxidation state of the copper cores. Notably, this process is involved in the temperature range where an increased SCR activity is associated with the passage to the f-SCR stage,^9^ which explains the role of the dual copper oxo centers in this process coherently. Reduction of Cu can only be expected under more aggressive reducing conditions (high *T*, low O_2_ pressure), which are characteristic of thermal activation of the samples in a vacuum.

Finally, it should be mentioned that NO oxidation can also occur within the zeolite cages with the assistance of the BAS centers only. The related mechanistic schemes and their energetics have been elaborated elsewhere.^38,39^ Notably, the BAS of H–CHA play an essential role in stabilizing the transition states. Two possible reaction pathways have been distinguished, operating in a low- and high-temperature range of the NO oxidation. The change from the low-to high-temperature regime can be tuned by controlling the BAS strength, which can be achieved, e.g., by substituting various trivalent metal atoms (such as B, Ga, or In) for the framework Al.

### 3.3 Reactivity of NO/O_2_ with Segregated CuO Nanocrystals

Since in the case of the *i*-CuSSZ-13 sample, our previous HR-TEM observations revealed the presence of segregated CuO nanocrystals of the (111) facet and 3–5 nm size,^40^ we examined their interaction with the NO/O_2_ reactants as well. A relevant set of conceivable reactions is collated in Table 4. The reactions were grouped according to their mechanistic features (interaction with gaseous O_2_, formation of NO_*x*_^δ−^ adspecies, and NO_*x*_ release) and labeled “**O**” to indicate the oxide origin of these active centers.

**Table 4:** Possible Reactions Associated with the Interaction of O_2_ and NO with the (111) Facet of the CuO Nanocrystals and Formation of NO_2_, Together with the Corresponding Reaction Energetics (Δ*E*_r_)

reaction type	labeling	reaction equation	Δ*E*_r_/eV
interaction of O_2_ with CuO(111)	O.1.1	2Cu^2+^ + O_2(g)_ ↔ Cu^2+^–O_2(ads)_–Cu^2+^	–0.29
	O.1.2	Cu^2+^–O_2(ads)_–Cu^2+^ + 2O^2−_(surf)_^ ↔ 2Cu^2+^ + 2[O_(surf)_O_(__ads)_]^2–^	0.95
	O.1.3	2Cu^2+^ + [O_(surf)_O_(ads)_]^2–^ ↔ 2Cu^+^ + V_O_^••^+ O_2(g)_	1.65
	O.1.4	4Cu^2+^ + 2O^2–^_(surf)_ ↔ 4Cu^+^ + 2 V_O_^••^+ O_2(g)_	4.50
formation of NO_*x*_^δ−^ adspecies	O.2.1	Cu^2+^ + NO_(g)_ → Cu^2+^NO_(surf)_	–1.58
	O.2.2	Cu^2+^ + NO_(g)_ + 1/2O_2(g)_ → Cu^2+^NO_2(surf)_^δ−^	–0.63
	O.2.3	Cu^2+^ + NO_(g)_ + O_2(g)_ → Cu^2+^NO_3_^δ−^_(surf)_	–3.05
	O.2.4	Cu^2+^ + [O_(surf)_O_(ads)_]^2–^ + NO_(g)_ → Cu^2+^NO_2(surf)_^δ−^ + O_(surf)_^2–^	–3.47
	O.2.5	Cu^2+^ + 2[O_(surf)_O_(ads)_]^2–^ + NO_(g)_ → Cu^2+^NO_3(surf)_^δ−^ + 2O_(surf)_^2–^	–3.52
	O.2.6	2Cu^2+^ + NO_(g)_ + O_(surf)_^2–^ → Cu^+^ + Cu^2+^NO_2(surf)_^δ−^ + V_O_^••^	–4.78
release of NO_2_	O.3.1	Cu^2+^NO_2(surf)_^δ−^ → Cu^2+^ + NO_2(g)_	1.88
	O.3.2	Cu^2+^NO_3(surf)_^δ−^ + O_surf_^2–^ → Cu^2+^ + [O_(surf)_O_(ads)_]^2–^ + NO_2(g)_	2.39
	O.3.3	NO_(g)_ + [O_(surf)_O_(ads)_]^2–^ → O_(surf)_^2–^ + NO_2(g)_	–1.64
	O.3.4	NO_(g)_ + Cu^2+^NO_3(surf)_^δ−^ → Cu^2+^NO_2(surf)_^δ−^ + NO_2(g)_	–0.48
	O.3.5	Cu^2+^NO_2(surf)_^–^ + Cu^+^ + V_O_^••^→ 2Cu^+^ + NO_2(g)_ + V_O_^••^	1.70
	O.3.6	Cu^2+^NO_3(surf)_^–^ + Cu^+^ + V_O_^••^→ 2Cu^2+^ + O_(surf)_^2–^ + NO_2(g)_	0.15

#### 3.3.1 Interaction of O_2_ with the (111) Surface of CuO

The oxygen dynamics on the CuO nanocrystals consists of an associative and dissociative accommodation of O_2_ at lower temperatures and release of the lattice oxygen to the gas phase (with the concomitant formation of a vacancy) at elevated temperatures. Possible reactions of accommodation/release of O_2_ on the bare, oxidized, and reduced (111) surfaces, along with their energetics, are presented in the top panel of Table 4. A detailed description of the (111) CuO surface redox states and oxygen adspecies can be found in our previous paper.^9^ Among the investigated ways of O_2_ release, only the **O.1.3** route gives rise to the desorption peak centered around *T* = 400 °C, which agrees with the experimentally observed evolution of ^16^O^18^O (see below).

#### 3.3.2 Interaction of NO/O_2_ with the (111) CuO Surface

For a comprehensive description of the adspecies produced on the (111) surface of CuO after interaction with the adsorbed NO and O_2_, a series of NO_*x*_^δ−^ surface adducts (*x* = 1, 2, 3) was initially analyzed, taking into account various adsorption centers (Cu_3c_, Cu_4c_, and O_surf_), the nature of the bonding atom (N and O), and the attachment mode (monodentate, bidentate, and bridging) of the admolecules. The results showed that tricoordinated Cu_3c_ ions were the preferred adsorption sites for all types of NO_*x*_^δ–^ surface adducts.

NO molecule is adsorbed on the Cu_3c_ centers in the η^1^-N geometry preferentially, whereas NO_2_^δ−^ and NO_3_^δ−^ are anchored through the O atoms in a bridging fashion (see Figure S13 in Supporting Information). Such NO_*x*_^δ−^ adducts may appear on the stoichiometric surface of the segregated copper oxide nanocrystals due to the coadsorption of NO and O_2_ or upon attack of gaseous NO on the surface peroxy groups. The energies of the corresponding **O.2.1**–**6** processes are summarized in Table 4, and the molecular diagrams of the individual reactions are presented in Figure S14a in Supporting Information and discussed in terms of their thermodynamics therein (Figure S14b-c). The NO_*x*_^δ−^ adducts gain a partial negative charge (δ) via the collective transfer of the electron density from the CuO framework. In turn, anionic entities can be formed with the participation of the lattice O^2–^ ions that act as a source of the electrons (**O.2.6**).

#### 3.3.3 Release of Gaseous NO_2_ from the (111) CuO Surface

**Figure 10:**
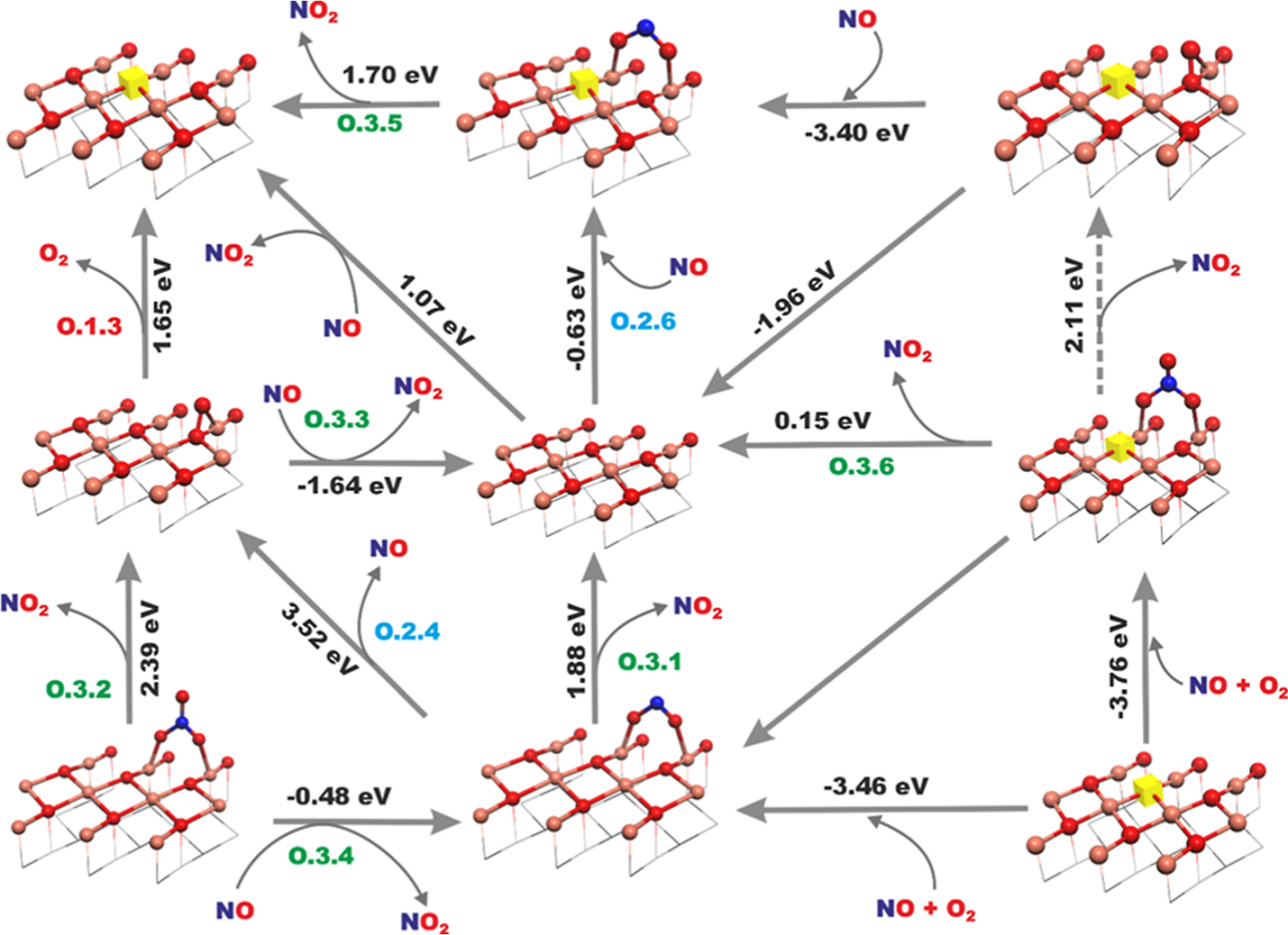
Network of elementary steps associated with the interaction of NO/O_2_ with the (111) surface of CuO leading to the formation of NO_2_. Labeling of the reaction pathways according to Table 4. Atoms color coding: Cu–pink, O—red, N—blue, V_O_—yellow cube.

**Figure 11:**
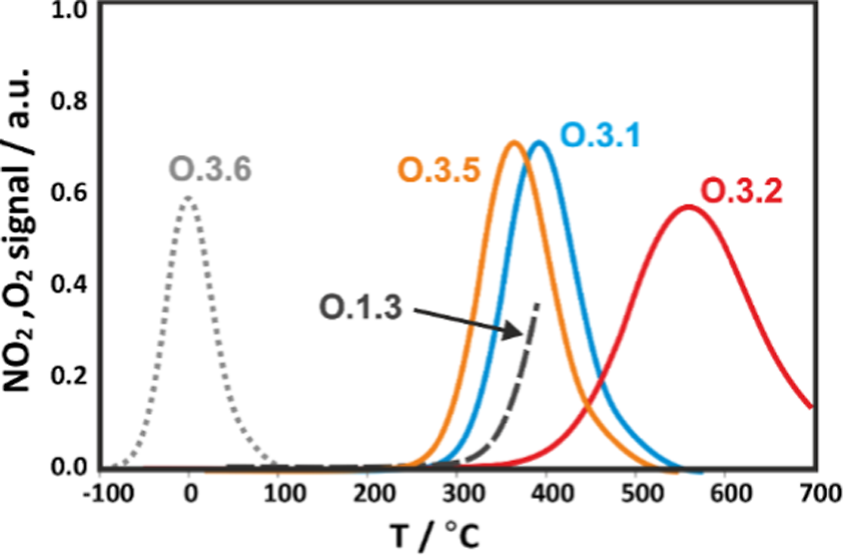
Simulated temperature profiles of the NO_2_ release along the **O.3.1**, **O.3.2**, **O.3.5**, and **O.3.6** routes, together with the profile of oxygen evolution along the **O.1.3** route (black dashed line) on the (111) surface of CuO nanocrystals.

The chemical reactions that represent the possible mechanistic routes of gaseous NO_2_ release/production (**O.3.1–6**) are collated at the bottom of Table 4, and the complex network of possible molecular events (with their energetics) is shown in Figure 10. The thermodynamic regions of the NO_2_ formation were then determined by FPT modeling, and the resulting release profiles as a function of temperature (for *p*_NO_2__/*p*_0_ ∼ 10^–4^ equivalent to the experimental NO_2_ level) are shown in Figure 11. A direct release of NO_2(g)_ from the (111) surface (reaction **O.3.1**) has an energy cost of 1.88 eV. In contrast, decomposing the NO_3_^δ–^ surface adduct (**O.3.2**) and leaving behind a peroxo anion are significantly more demanding (2.39 eV). These energies correspond to the NO_2_ evolution peaks at *T* = 400 and 575 °C, respectively (Figure 11). The NO_2_ molecules can also be formed by the interaction of NO_(g)_ with the surface peroxo groups (**O.3.3**) with Δ*E*_r_ = −1.64 eV, or by comproportionation of NO_3_^δ–^ adspecies with gaseous NO (**O.3.4)**, which is less exoenergetic (Δ*E*_r_ = −0.48 eV). However, it should be noted that the NO_2_ molecules produced at lower temperatures remain on the surface since their desorption occurs only around ∼400 °C (Figure 11, reaction **O.3.1**), i.e., in the region where NO_2(g)_ is thermodynamically unstable.

For a comprehensive account, we also considered a scenario in which a NO molecule can extract lattice oxygen, forming a NO_2_^–^_(surf)_ adspecies, stabilized near the generated oxygen vacancy (**O.2.6**). The binding energy of the resultant NO_2_^–^ adduct (Δ*E*_ads_ = 1.70 eV) implies that the corresponding peak of NO_2_ desorption (**O.3.5)** almost coincides with the peak associated with the **O.3.1** process (Figure 11).

Noting that oxygen vacancies can be formed above 350–400 °C, following the **O.1.3** route (see the black dashed line in Figure 11), the surface NO_3_^δ−^ adspecies, stable until this temperature (reaction **O.3.2**), can decompose into NO_2(g)_ with simultaneous refilling of the vacancy by the oxygen atom left behind (**O.3.6**). Then, the energy of the NO_2_ evolution decreases significantly (Δ*E*_r_ = 0.15 eV), and the temperature limit of oxygen vacancy formation can be associated with the starting point of the decomposition of NO_3_^δ−^. As a result, NO_2_ formation in the experimental temperature range can be related to the **O.3.1**, **O.3.2**, and **O.3.5** processes only. However, the **O.3.2** route occurs above 500 °C and appears to be unproductive because NO_2(g)_ is then thermodynamically unstable.

### 3.4 Spectroscopic and Catalytic Investigations into NO and O_2_ Reactivity with CuSSZ-13

#### 3.4.1 IR Studies of NO Oxidation

**Figure 12:**
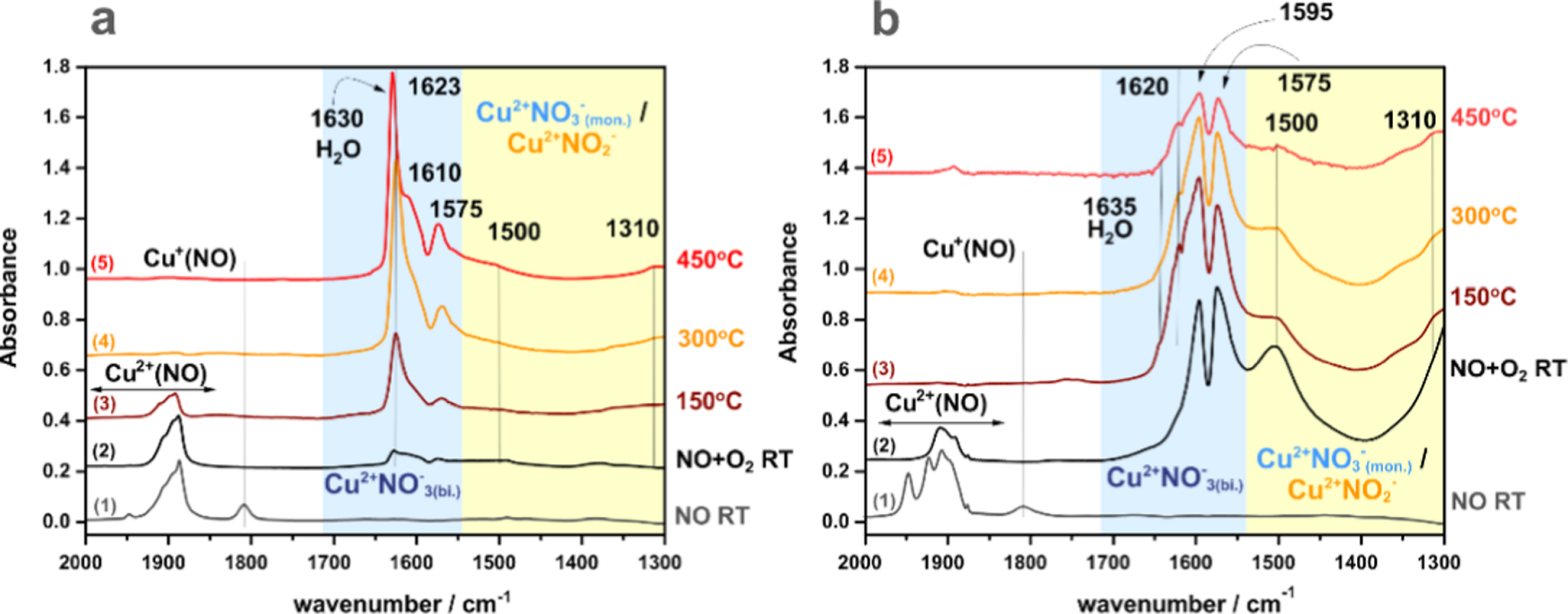
Temperature evolution of IR spectra after NO and NO + O_2_ adsorption on *i*-CuSSZ-13 (a) and *o*-CuSSZ-13 (b) samples. All spectra were recorded after quenching at room temperature.

The IR spectra of the *i*-CuSSZ-13 and *o*-CuSSZ-13 samples recorded during the interaction of NO and O_2_ at varying temperatures (Figure 12) show a strong influence of copper speciation on NO oxidation by O_2_, following the complex network of the reaction pathways predicted by DFT modeling (Figures 4 and 9). The initial sorption of NO (spectrum 1) leads to the formation of Cu^2+^NO species, with fingerprint bands in the range of 1950–1880 cm^–1^ and a minor fraction of Cu^+^NO (1810–1800 cm^–1^). Copper reduced at the beginning can be associated with the reaction between Cu^2+^OH^–^ and NO with HONO formation (Table 1, **M.1.1–3)**. The IR spectra prove its feasibility even at low temperatures, which occurs readily due to the low activation energy (0.25 eV).^9^ Adding O_2_ quenches immediately the Cu^+^–NO band, and divalent copper nitrate species are directly formed, which is especially pronounced for the *o*-CuSSZ-13 sample (spectrum 2 in Figure 12b) of the higher content of dimeric Cu-oxo species. The bands at 1620, 1595, and 1575 cm^–1^ were ascribed to the symmetric stretching modes of bidentate NO_3_^–^, whereas the bands at 1500 and 1310 cm^–1^ come from the antisymmetric and symmetric modes of monodentate NO_3_^–^ species.^21,25,56−60^ However, these bands may also originate from antisymmetric and symmetric stretchings of the N-bonded nitrites (NO_2_^–^).^61^ At *T* ≥ 300 °C, the remaining mononitrosyl adducts are oxidized into the nitrates or can desorb.

In the case of the *i*-CuSSZ-13 catalyst, a dominant route of the nitrates/nitrites formation involves the abundant intrazeolite Cu^2+^–OH^–^ active sites (**M.1.2–3** and **M.3.1–3**), while for *o*-CuSSZ-13, these species are produced primarily on the bridging Cu^2+^–O^2–^Cu^2+^ centers (**D.2.1–3** and **D.3.1–3**), which dominate over Cu^2+^–OH^–^ in this sample. The nitrate/nitrite species begin to decompose at 450 °C, in excellent accordance with the predictions based on the Δ*G*(*p*,*T*) diagrams (Figure 1). The formation of water (IR band at 1630–1635 cm^–1^) is seen (Figure 12), especially in the case of the *i*-CuSSZ-13 sample of a much higher Cu^2+^–OH^–^ content than in *o*-CuSSZ-13, and may result from reoxidation processes involving an O_2_ reaction with the Cu^+^ and H^+^ cations (reactions **M.2.3** and **M.5.1**) or from the interaction of HONO with Brønsted centers.

#### 3.4.2 EPR of NO_*x*_ Formation and Evolution

**Figure 13:**
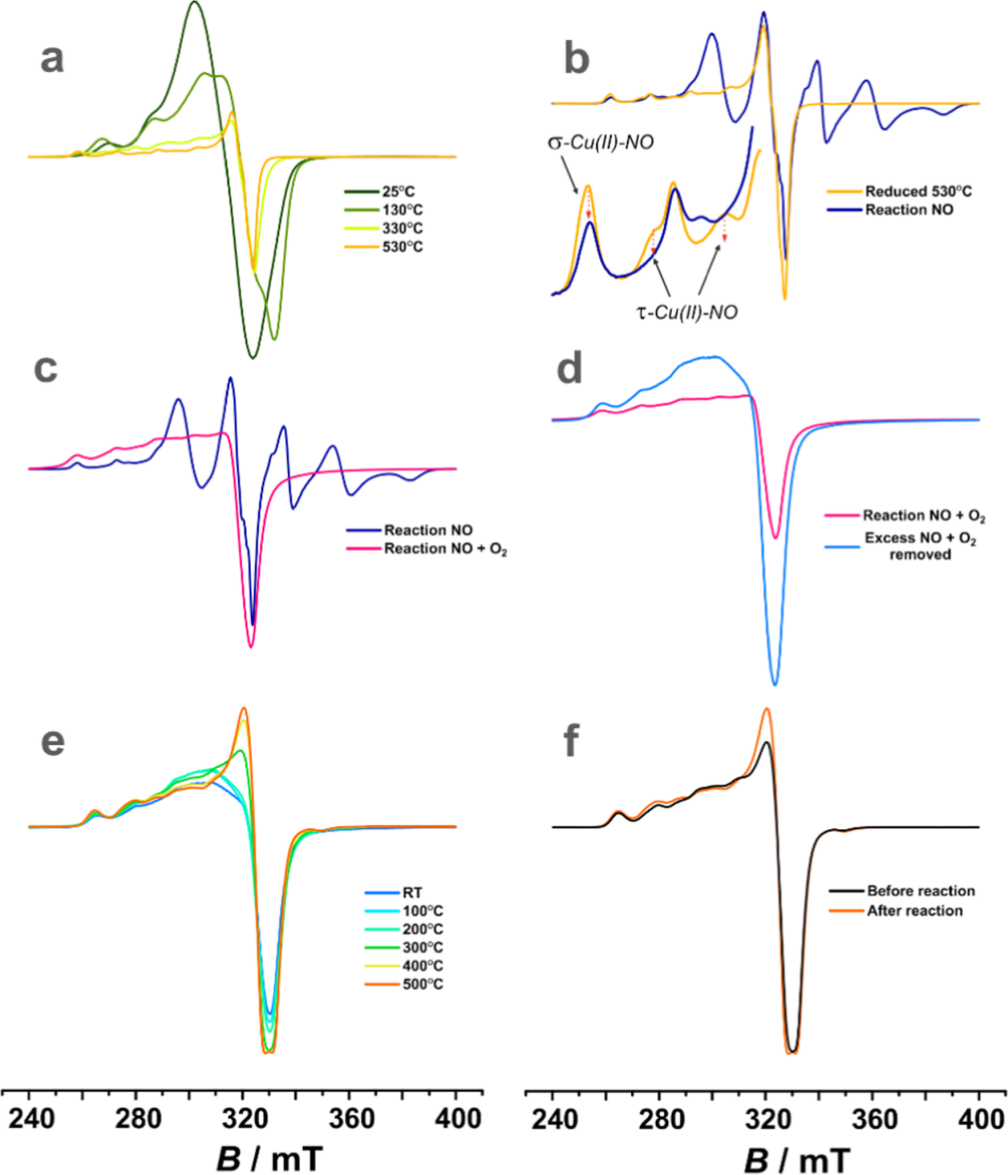
Evolution of X-band EPR spectra of the *o*-CuSSZ-13 sample after reduction in NH_3_ (a), subsequent sorption of NO (b) and O_2_ (c), followed by desorption of the excess gases (d), temperature evolution of the resulting signal (e), and comparison with the EPR signal of the initial oxidized sample (f). All spectra were registered in liquid nitrogen. In (b), the arrows indicate the loss of the Cu^2+^ signal due to forming a diamagnetic Cu^2+^↑↓NO adduct.

The interaction of the copper centers with NO/O_2_ was next investigated with the EPR technique, which allowed for corroborative insight into the evolution of the paramagnetic Cu^2+^, Cu^2+^–NO_3_^–^, and Cu^+^–NO species. To produce the nitrate adducts, the copper centers were reduced earlier with ammonia (3Cu^2+^ + NH_3_ → 3Cu^+^ + 3H^+^ + 1/2N_2_; see our previous article for details^40^), and the progress of this reaction was monitored by the loss of EPR intensity of Cu^2+^ (Figure 13a).

Upon contact of the reduced sample with NO (Figure 13b), a monoclinic signal (*g*_*xx*_ = 2.008, *g*_*yy*_ = 2.015, *g*_*zz*_ = 1.889, *A*_*xx*_ = 19.54 mT, *A*_*yy*_ = 21.35 mT and *A*_*zz*_ = 23.97 mT) of the Cu^+^–NO adduct was developed.^62,63^ Subsequent oxygen introduction, even without any further thermal treatment, led to the instant formation of nitrates/nitrites and reoxidation of the Cu centers (reaction **M.3.2**, Table 1), in agreement with previous literature.^17,64^ The characteristic EPR signal of nitrates with *g*_*xx*_ = 2.055, *g*_*yy*_ = 2.161, *g*_*zz*_ = 2.345, *A*_*xx*_ and *A*_*yy*_ unresolved, and *A*_*zz*_ = 15.82 mT^64^ could quickly be observed after the removal of excess O_2_ that broadens the spectrum (Figure 13c,d). The resultant Cu^2+^ nitrates were relatively stable while heated to 300 °C (Figure 13e). They then slowly decomposed, leaving oxidized copper(II) virtually in the same amount as initially detected (Figure 13f), confirming the self-consistence of the observed changes.

The EPR results can be interpreted based on the thermodynamic diagram shown in Figure 1 (stability of the CuNO_*x*_ and Cu–nitrosyl adducts). In the absence of O_2_, the mono- and dinitrosyls are the dominant forms for oxidized and reduced Cu centers (valid under SCO conditions and those under which the EPR and IR measurements were performed). The formation of nitrosyls and their stability depends on the adsorption locus, and for the 6MR sites, Cu^2+^-NO, Cu^2+^(NO)_2_, and Cu^+^-NO species are expected to form with the thermal stability limits of 100 °C for the Cu^2+^ nitrosyls, 70 °C for the Cu^+^(NO)_2_ dinitrosyls, and 250 °C for the Cu^+^–NO adducts (*p*_NO/_*p*^0^ ∼ 10^–3^). In the case of the 8MR sites, the Cu^2+^–NO, Cu^+^–NO, and Cu^+^(NO)_2_ adducts are expected in the experimental range of *T* and *p*_NO_. The formation of diamagnetic nitrosyls accounts for the observed changes in the copper EPR signal after NO adsorption (Figure 13b), where only half of its initial intensity (measured before the reduction step) is transformed into the Cu^+^–NO signal [note the coexistence of the paramagnetic Cu^+^–NO and diamagnetic Cu^+^–(NO)_2_ adducts at 8MR as well as the sole presence of the dinitrosyls at 6MR]. The changes in the EPR intensity of the remaining unreduced Cu^2+^ (see Figure 13b) can also be explained by the thermodynamic diagrams shown in Figure 1. Upon NO sorption, the hyperfine component assigned to the Cu^2+^/8MR species is completely quenched (see the corresponding features indicated by the arrows in Figure 13b) due to the formation of an EPR silent Cu^2+^–NO adduct (or other diamagnetic nitrosyls) at the temperatures of the spectroscopic measurements.

For the reduced copper in the presence of O_2_, the thermodynamic diagrams (Figure 1c,d) are dominated by the Cu^2+^NO_3_^–^ species, which remain stable up to ∼350 °C (in good agreement with the EPR results, see Figure 13e). Due to the high stabilization energy, it is most likely that nitrate decomposition occurs through the redox pathway, where the nitrates are first converted into the nitrites (see the development of the IR 1310 cm^–1^ feature in Figure 12) along the reactions **M.3.3–4** (Table 1). It is followed next by an electroprotic reoxidation of bare Cu^+^ with O_2_ (**M.5.1**) to restore the EPR signal of Cu^2+^ in its virtual initial intensity (Figure 13f). Such an involved pathway allows for circumvention of the high energy cost of the direct decomposition of nitrates into NO and O_2_.

#### 3.4.3 TPSR Studies of NO Oxidation

**Figure 14:**
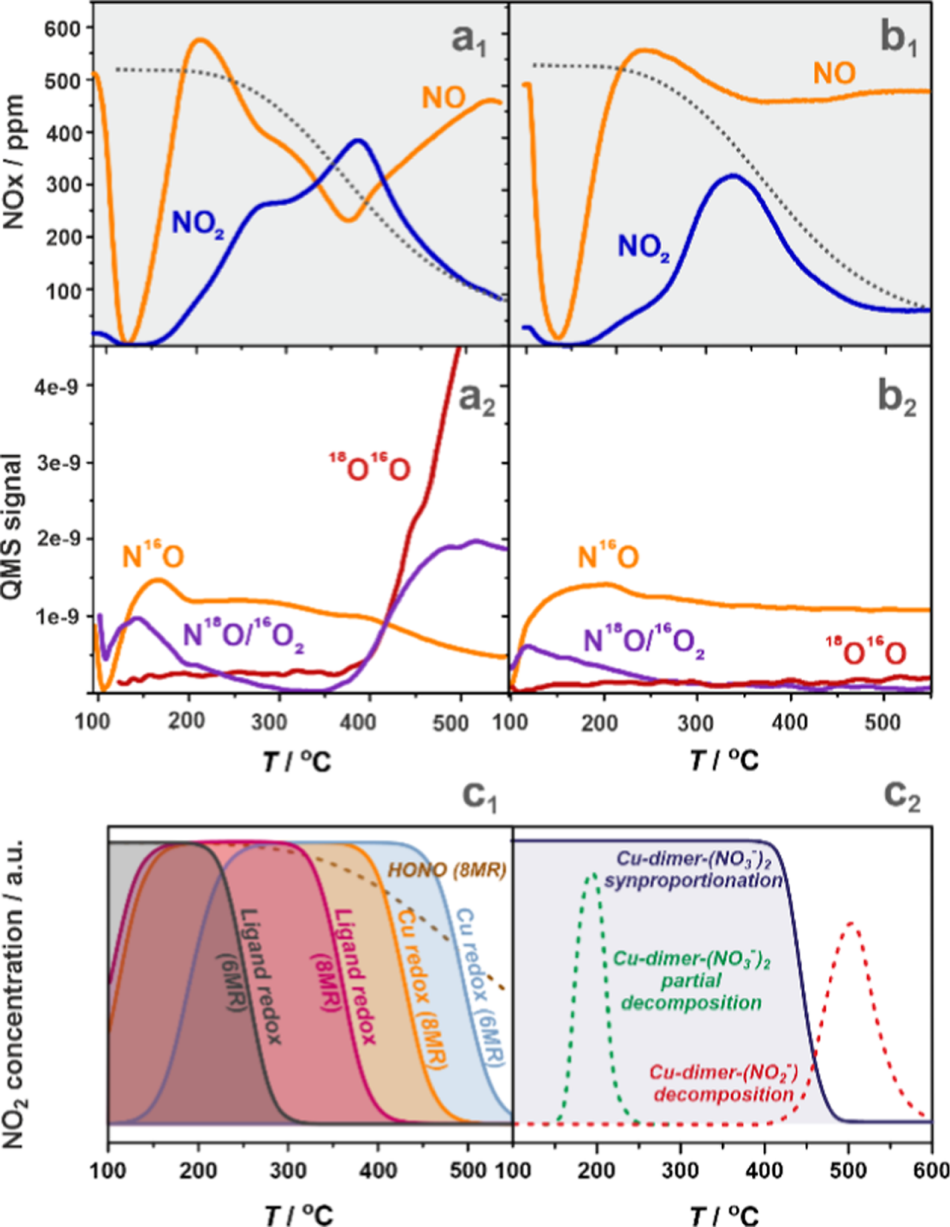
TPSR profiles of NO oxidation with O_2_ over *i*-CuSSZ-13 (a_1_,a_2_) and *o*-CuSSZ-13 (b_1_,b_2_) zeolites (500 ppm of NO, 4.5% O_2_, He as carrier gas) along with DFT/FPT predicted temperature windows of NO_2_ evolution for the monomeric (c_1_) and dimeric (c_2_) Cu species. Lower index (**1**) stands for the reaction with ^16^O_2_, while (**2**) stands for the reaction with ^18^O_2_. The dotted gray lines (a_1_,b_1_) indicate the equilibrium NO_2_ concentration in the gas phase. In panel (c_1_), the metal 8MR and 6MR redox refers to the aggregated reactions **M.3.2–4**, the ligand 8MR and 6MR redox refers to the aggregated reactions **M.4.2–4**, while HONO (8MR) refers to the **M.2.1** reaction. In panel (c_2_), the indicated reactions correspond to **D.5.1** (solid blue line), **D.4.4** (dashed green line), and **D.4.5** (dashed red line).

The results of selective catalytic oxidation of NO to NO_2_ (NO–SCO) on the *o*-CuSSZ-13 and *i*-CuSSZ-13 samples in the 100–550 °C temperature range are shown in Figure 14a,b. Interestingly, the observed NO_2_ evolution profiles and temperature windows are pretty similar to those observed in NO_2_-TPD experiments using CuSSZ-13 samples of various copper loading,^65^ with the intrazeolite Cu^2+^OH^–^ proposed as the prime active sites for NO_2_/NO interconversion through nitrate intermediates.

The evolving NO_2_ profile can be divided into low- (LT), *T* < 250 °C, mid- (MT), 250°C < *T* < 400 °C, and high-temperature (HT), 400°C < *T* < 500 °C, regions. The LT region is initially featured by reactive NO capture/release, which can be associated with the intrazeolite adsorption/desorption dynamics of the copper(II) nitrosyl adducts and the formation of nitrates following the IR and EPR results (Figures 12 and 13d). The appearance of N^18^O along with N^16^O, when ^18^O_2_ is used as an oxidant (Figure 14a_1_,b_1_), indicates that the isotopic scrambling of NO with the labeled nitrate species occurs already at low temperatures, with the latter being readily produced in the presence of NO and ^18^O_2_ under these conditions. The evolution of NO_2_ begins above ∼150 °C, and there is a significant difference in the progress of this reaction between both samples, with *i*-CuSSZ-13 being much more active (40% of NO conversion) than *o*-CuSSZ-13 (15%). The marked disparity in the Cu^2+^OH^–^ content in the *i*-CuSSZ-13 (255 μmol·g^–1^) and *o*-CuSSZ-13 (30 μmol·g^–1^) samples suggests that the copper-hydroxo centers are primarily responsible for the pronounced NO conversion, following the HONO pathways (**M.1.1–3**, Table 1). This triggers a set of successive reactions that lead to the formation of nitrites (**M.2.2**) and nitrates (**M.3.1–2**). Subsequent NO_2_ evolution can be associated with the direct detachment (**M.3.4)** and the comproportionation processes (**M.4.3)** involving the 8MR and 6MR centers. The predicted temperatures of the NO_2_ release peaks (Figure 14c_**1**_) indicate that these desorption pathways extend into the MT region.

The NO_2_ maximum around 400 °C for *i*-CuSSZ-13 is more pronounced and is moved to 350 °C for the *o*-CuSSZ-13 catalyst of higher content of the dual copper-oxo centers (Figure 14c_2_). Therefore, it can be traced back to the comproportionation reactions between various bridging nitrate species and gaseous NO (**D.5.1–2**) and the subsequent decomposition of nitrites. Notably, dimeric copper centers have been proposed previously to be active in the NO oxidation into NO_2_.^26^

In the HT range, the decrease in the evolution of NO_2_ results from the gradual depletion of the nitrate adspecies, revealed by the IR (Figure 12) and EPR (Figure 13e) investigations, as well as the predicted thermodynamic stability limit of gaseous nitrogen dioxide (Figure 14a_1_,a_2_, gray dotted lines). An intensive isotopic oxygen exchange and the appearance of ^16^O^18^O along with N^18^O > N^16^O when ^18^O_2_ was used as an oxidant were observed for *i*-CuSSZ-13 and absent for *o*-CuSSZ-13 (Figure 14b_1_,b_2_), which indicate a transient formation of the nitrate/nitrite on the surface of the segregated CuO nanocrystals. Being unstable at such temperatures, they decompose and release the observed N^18^O, N^16^O, and ^16^O^18^O isotopomers. The formation of oxygen vacancies and surface peroxy species on CuO nanocrystals (see Section 3.3) facilitates the exchange of isotopes, leading to the intense burst of the ^18^O^16^O isotopomer above 400 °C (predicted nicely by the DFT/FPT modeling; see Figure 11). As a result, using the calculated desorption profiles, the experimentally observed NO_2_ peaks can be associated with the multiple NO/O_2_ reaction pathways described in Sections 3.2.2, 3.2.3, and 3.3.2.

In summary, the LT onset of NO_2_ production results from two types of reactions: ligand redox reactions (at 6MR and 8MR sites, **M.4.2–4**) and the reaction with the HONO intermediate at 8MR (**M.2.1**), both involving the mononuclear Cu^2+^ centers. The latter also contributes to the formation of NO_2_ at higher temperatures. In addition, in the LT regime, the nitrates accumulated at the dual-Cu centers contribute to NO_2_ production (**D.4.4**). The NO_2_ evolution peak around 300 °C (more intense for *i*-CuSSZ-13 than for *o*-CuSSZ-13) arises from the intricate Cu-redox processes, which are initiated by the reaction of Cu^2+^OH^–^ with NO, leading to HONO and reduced Cu^+^ (this opens the Cu redox pathway in 6MR sites even though HONO cannot reduce the initial Cu^2+^/6MR centers). The maximum of NO_2_ evolution at 350–400 °C, present for both samples, arises from the high-temperature end of the Cu redox cycle at the 8MR sites (**M.3.2–4**, Figure 3b_2_), which includes the decomposition of all nitrites and the comproportionation of the nitrates accommodated in the copper dual centers with NO. Therefore, the overall contribution of the Cu cations accommodated in the 8MR sites decides that the intensity of the NO_2_ peak is more pronounced for the *o*-CuSSZ-13 sample (Figure 14b_1_). The Cu redox for the 6MR sites and the dual copper-oxo centers give rise to a broad window of the NO_2_ generation, extending to the HT region, which is seen for the *i*-CuSSZ-13 sample (Figure 14a_1_). The HT production of NO_2_ is driven by the decomposition of the Cu^2+^–NO_2_^–^ species (**M.3.4**), comproportionation of Cu^2+^(NO_3_^–^)_2_Cu^2+^ with NO (**D.5.1**), and decomposition of the resulting nitrites bound at the dual Cu centers (**D.4.5**). At HT, in the case of the *i*-CuSSZ-13 sample, for which an intensive isotopic scrambling was observed, the NO_2_ production on the (111) surface of the segregated CuO nanocrystals results from the reactions **O.3.1** and **O.3.5**. However, the produced NO_2_ decomposes into NO and O_2_ in the gas phase at HT. It should be emphasized that all of the IR, EPR, and NO oxidation TPSR experiments can be accounted for with reasonable accuracy at the PW91/DFT-U + D level of theory. Surprisingly, this agreement appeared inferior when using the hybrid HSE06 functional in the benchmarking calculations (see Supporting Information, Section S2).

### 3.5 Molecular Events Underlying the NO–SCO Reaction Mechanism

**Figure 15:**
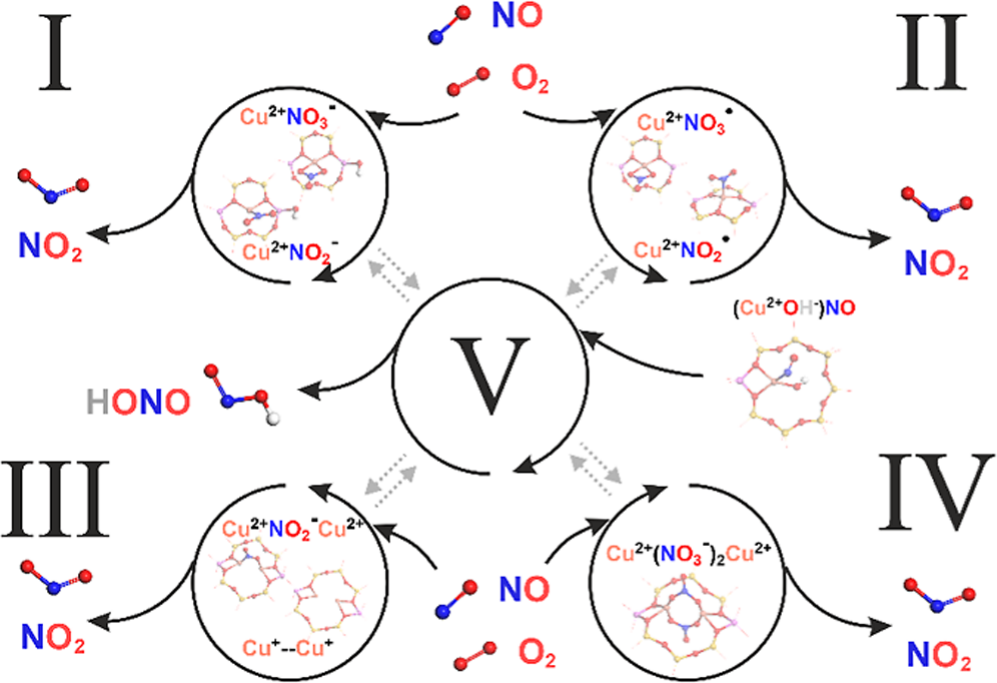
Schematic overview of possible pathways of the NO/O_2_ interaction and generation of NO_2_ on the copper active sites of the CuSSZ-13 zeolite. **I**—metal redox Cu^2+^/Cu^+^ pathway on single copper centers, **II**—ligand redox pathway on oxidized Cu^2+^, **III**—redox Cu^2+^/Cu^+^ pathway of dimeric copper-oxo centers, **IV**—ligand redox pathway on dimeric copper-oxo centers, **V**–HONO cycle involving hydroxylated copper centers.

The general mechanism of NO–SCO in the CuSSZ-13 zeolite is an intricate blend of the interconnected reaction cycles characterized by the involvement of various reactive NO_*x*_^δ−^ and HONO species, and its graphic representation is shown in Figure 15. The established mechanistic cycles (labeled I, II, III, IV, and V in Figure 15) depend on copper nuclearity and a sustainable alternation of the oxidation states. Redox cycles involving single (I, II) and dual centers (III, IV) rely on the reduction of Cu^2+^ to Cu^+^, allowing for the formation of anionic nitrate/nitrite ligands. For divalent copper centers, the NO–SCO reaction can occur either through the direct release of NO_2_ from the NO_*x*_^δ−^ adspecies (ligand-redox cycle II) or through the formation of nitrates/nitrites on the dual copper-oxo active centers, followed by the reaction with gaseous NO (comproportionation cycle IV). The mobile, encaged HONO intermediates (which evolve through cycle V) provide chemical communication between the reaction cycles. The HONO species are produced during the reaction of NO with copper hydroxyls, accelerating cycle I by generating transient Cu(I) centers (**M.1.2–3** and **M.2.1**). The latter upon interaction with NO and O_2_ are readily transformed into the corresponding nitrates (**M.3.1–2**).

Furthermore, in segregated CuO nanocrystals, apart from analogous surface nitrate pathways, direct NO oxidation into NO_2_ by surface peroxy species, produced upon oxygen dissociation, operates at higher temperatures (above 400 °C), i.e., beyond the thermodynamic stability limit of the gas phase NO_2_.

## 4 Conclusions

Three principal routes of NO_2_ production upon NO and O_2_ interaction with CuSSZ-13 include the HONO pathway, the metal-redox pathway, and the ligand-redox pathway, with the comproportionation of NO with the surface nitrates and a versatile HONO behavior playing a crucial role. Among single copper sites, the Cu^2+^–OH^–^ species are the active centers for NO_2_ formation through the HONO pathway, regardless of their location (6MR and 8MR sites). The redox-reluctant bare Cu^2+^ centers accommodated in the 6MR sites are not expected to participate in the cationic metal redox process of NO oxidation, in contrast to the Cu^2+^ cations located in the 8MR, which are readily reducible by the HONO intermediates. Dimeric copper centers with oxo and peroxy bridging moieties can produce a variety of nitrates and nitrites through ligand redox mechanisms, which trigger the evolution of NO_2_ along direct release or nitrogen comproportionation routes. Segregated nano-CuO crystals contribute to NO conversion only at high temperatures (*T* > 400 °C), leading to an intensive scrambling of ^18^O_2_/^16^O_2_ and N^16^O/N^18^O. The thermodynamic instability of NO_2_ in this temperature region substantially reduces the overall extent of conversion of NO to NO_2_.

## Supporting Information Available

Modeling of the Al distribution in the SSZ-13 zeolite; comparison of the PW91 and HSE06 functionals; structure and energetics of Cu adducts with O_2_, NO, NO_2_, and NO_3_; simulation of partial pressure of HONO on thermodynamic reaction ξ(*T*) profiles; and reactivity of CuO nanocrystals with NO (PDF)

## Dedication

This paper is dedicated to the memory of Professor Krystyna Dyrek.
