# Supplementary material for: First-Principles Thermodynamic Background of the Comprehensive Reaction Network of NO Oxidation over CuSSZ-13 Catalysts—Influence of Copper Speciation and Interpretation of TPD and TPSR Profiles
Source: ACS Catal. 2025 Jan 30;15(4):2715–34. doi: 10.1021/acscatal.4c06619 (PMC11851786; doi:10.1021/acscatal.4c06619)
Supplement: Supplementary file 1 — cs4c06619_si_001.pdf [file cs4c06619_si_001.pdf]

Electronic Supporting Information for

First Principles Thermodynamic Background of the Comprehensive Reaction Network of NO Oxidation over CuSSZ-13 Catalysts - Influence of Copper Speciation and Interpretation of TPD and TPSR Profiles

Bartosz Mozgawa,[1,2] Filip Zasada,[1] Monika Fedyna,[1] Kinga Góra-Marek,[1] Chengyang Yin,[3] Zhen Zhao,[3] Zbigniew Sojka[1], Piotr Pietrzyk[1]**

[1] Faculty of Chemistry, Jagiellonian University, ul. Gronostajowa 2, 30-387 Krakow, Poland

[2] Doctoral School of Exact and Natural Sciences, Jagiellonian University, ul. prof. S. Łojasiewicza 11, 30-348 Krakow, Poland

[3] Institute of Catalysis for Energy and Environment, College of Chemistry and Chemical Engineering, Shenyang Normal University, Shenyang, Liaoning, 110034, China

Corresponding Authors

* Faculty of Chemistry, Jagiellonian University, ul. Gronostajowa 2, 30-387 Krakow, Poland, e-mail: piotr.pietrzyk@uj.edu.pl (Piotr Pietrzyk), sojka@chemia.uj.edu.pl (Zbigniew Sojka)

Table of Contents

S1. Al distribution within the SSZ-13 zeolite framework 3S

S2. Comparison of the PW91 and HSE06 functionals and U parameter selection...........8S

S3. Slab Model of the CuO (111) Surface…………………………...…………………11S

S4. Structure and Energetics of Copper Adducts with O_2, NO, NO_2, and NO_3..............12S

S4.1. NO and NO_x interaction with the isolated Cu sites...12S

S4.2. Dual Cu centers in CuSSZ-13 under real conditions (T, p_{O2}).............................17S

S4.3. Interaction of NO and NO_x with dual Cu sites...20S

S4. Effect of HONO Pressure 26S

S5. Reactivity of CuO Nanocrystals with NO 28S

REFERENCES 30S

S1. Al distribution within the SSZ-13 zeolite framework

The catalytic properties of an SSZ-13 zeolite are influenced by the arrangement of aluminum atoms within the framework [1,2], which affects the concentration and stability of the exchanged mono- and divalent cations as well as the metal-oxo species [3,4]. Additionally, for monovalent cationic species and protons, the aluminum distribution determines the distance between active sites, influencing their potential for cooperation during catalytic action. Several catalytic studies have demonstrated that zeolites with the same chemical compositions but differing in aluminum arrangements can exhibit distinct catalytic behavior [5,6,7]. Therefore, understanding the aluminum distribution within the framework is essential for evaluating zeolites potential for specific catalytic reactions [8,9,10,11,12]. However, papers that describe reliably the experimentally determined exact positions of aluminum atoms in the chabazite framork for the particular Si/Al ratio and the applied synthesis method are rather hardly available [13].

Isolated Al Centers

As the chabazite lattice is relatively simple and symmetric, there are essentially two types of isolated T-sites, defined by the location in either 6MR or 8MR. Such negatively charged T-sites were counterbalanced by H^+ (formation of the BAS centers, **Figures S1a** and **S1b**) and Cu^+ (**Figures S1c** and **S1d**), and it was found that the energies of the two BAS locations were nearly identical (ΔE^{DFT} = –0.02 eV). On the contrary, reduced copper cations tends to be better stabilized in 6MR (by –0.58 eV), due to higher coordination number in this location.

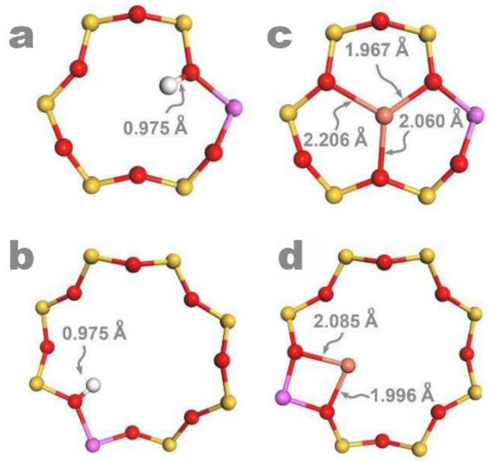

Figure S1. Optimized structures of single Al sites in CHA zeolite in 5-MR (6-membered ring, top panels) and 8-MR (8-membered ring, bottom panels), counterbalanced by H^+ (left panels) and a Cu^+ cation (right panels).

Al pairs

The situation becomes more complicated if two Al atoms are present, as their distribution within the framework may be influenced by the kinetic (synthesis protocols) and the thermodynamic constraints during the zeolite formation. Indeed, as recently reported, kinetic factors may compete (or overlap) with energetic interactions, yet the geometric- and/or electro-static-based thermodynamic properties of structure directing agents used in the synthesis can be applied to predict and design the Al distribution in zeolite frameworks [14]. Aluminum pairs can be divided into three categories, where two Al are located in 6MR, 8MR or D6R (hexagonal prism double-ring 6MR), which can be referred to as $\boldsymbol{\sigma}_{1\text{-n}}$, $\boldsymbol{\tau}_{1\text{-n}}$, and $\boldsymbol{\omega}_{1\text{-n}}$ respectively [2], where *n* represents the position of the second Al atom in the given ring (see **Figure S2a)**. Schneider *et al.* have reported notable differences in the Al−Al interactions within the SSZ-13 zeolite lattice when H^+ or Cu^{2+} are present, and this findings highlight the energetic factors that may govern Al site preferences during the one pot copper zeolite synthesis, and illustrate possibly how these preferences can affect subsequent ion exchange processes [15]. Palouci *et al.* in turn, have presented the probability calculations for dimer exchange for all unique 2Al configurations in various zeolite topologies, revealing that most Cu dimers are formed at Al−Al distances

of 5 to 9 Å [16]. As a result, 8MR and 10MR are favorable for dimer formation in different zeolite structures. In contrast, 6MR favor monomer formation, while 4MR and 5MR are generally less favorable for Cu exchange due to steric repulsion. The 1–2 Al configuration inside single rings is excluded by the Löwenstein rule (apart from prism $\boldsymbol{\omega}_{1\text{-}2}$ positions) [17]. Based on these literature reports, we selected the most probable aluminum dimer arrangements in the zeolite framework, that can accommodate the Cu^{2+} cation, calculating next the energetics of corresponding models with the PW91-DFT+U method. The relative energies of the relaxed structures are presented in **Figure S2b**.

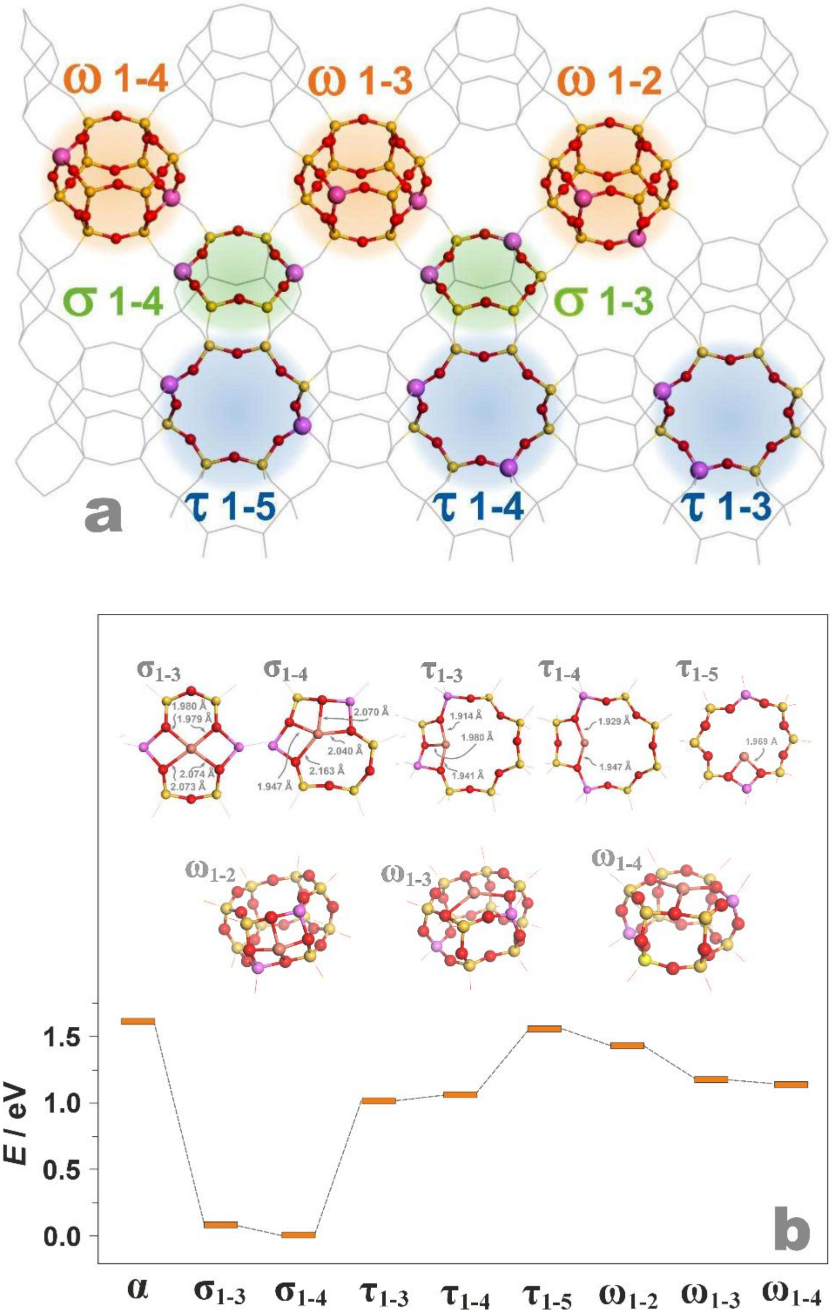

Figure S2. Nomenclature of Al pairs in CHA zeolite (**a**) together with relative energies of the relaxed zeolite unit cells containing Cu^{2+} (**b**).

The copper(II) stabilization energies depend on the site selected, and four cases can be distinguished. The lowest energy is obtained for copper accomodated in 6MR containing a sigma-type Al pair ($\boldsymbol{\sigma}_{1\text{-}3}$ and $\boldsymbol{\sigma}_{1\text{-}4}$), whereas copper in the tau and omega pairs with n = 3 and 4 ($\boldsymbol{\tau}_{1\text{-}3}$, $\boldsymbol{\tau}_{1\text{-}4}$, $\boldsymbol{\omega}_{1\text{-}3}$, and $\boldsymbol{\omega}_{1\text{-}4}$) are less stable by about 1 eV. Copper binding to the $\boldsymbol{\tau}_{1\text{-}4}$ and $\boldsymbol{\alpha}$ centers is the weakest, as in both cases the distance between the aluminum ions in the unit cell is the

greatest. It can be expected then that during ion exchange with Cu^{2+}, the dual Al sites will be occupied in the order of $\boldsymbol{\sigma} \rightarrow \boldsymbol{\tau} \rightarrow \boldsymbol{\omega}$, with the monomeric $\boldsymbol{\alpha}$ centers at the end. Based on the above considerations, for the construction of the geometric models we selected $\boldsymbol{\sigma}_{1\text{-}4}$ (6-MR), $\boldsymbol{\tau}_{1\text{-}4}$ (8-MR), and $\boldsymbol{\tau}_{1\text{-}5}$ (for comparison) as the possible accomodation centes for the copper cations.

S2. Comparison of the PW91 and HSE06 functionals and *U* parameter selection

The GGA functionals, and PW91 in particular, are typically used for periodic DFT calculations of various metal-zeolite NO-SCO and NH_3-SCR catalytic systems (see, for instance [18,19,20,21,22]), as they provide a reasonable balance between the acceptable accuracy and the cost of calculations. However, GGA has an obvious limitation, resulting mainly from the inadequate description of electron-electron self-interaction. To address this issue, two main approaches have been developed: hybrid functionals (*e.g.*, HSE06), which incorporate a portion of the exact exchange from Hartree-Fock, and DFT+U method, in which the on-site Coulomb interaction term is added to the Hamiltonian for more accurate treatment of the delocalized electrons. The latter approach is commonly applied in solid-state modeling, including zeolites [23,24,25,26,27]. This correction, while being semi-empirical, requires a rational selection of the *U* parameter value, ensuring the best agreement between theoretically calculated properties and experimental data. In many studies concerning DFT modeling of Cu-containing zeolites, the Hubbard *U* parameter is set to 6 eV, as proposed by Isseroff and Carter [28], who scaled it based on the comparisons with the experimental lattice and dielectric constants for Cu_2O. However, for modeling the energetics of adsorption, which is crucial in our work, a better choice seems to be the *U* parameter at a lower level (4 to 5 eV) as proposed by Bhola *et al.* [23], based on the binding enthalpy of H_2 on CuO. The value of $U = 4$ eV was also proposed in modeling the redox properties of transition metal oxides [29].

As a result, we performed the benchmarking calculations changing the *U* value from 4 to 6 eV while modeling NO adsorption on Cu(I), Cu–Cu dimers, and Cu(II) centers, observing rather small changes (8% at maximum) in the energy. The closest agreement with the experimental results (desorption temperatures) was obtained for $U = 4$ eV, which was used for further DFT modeling.

Following Anggara *et al.*, for modeling the NO adsorption or NO-O_2 co-adsorption on Cu-N_xO_y/SSZ-13 system, hybrid functionals are more accurate when compared with pure PW91 [30]. In this work, Hubbard correction was not included. Therefore, for selected structures (NO_x structures based on the Cu^{2+} and Cu^+ active centers), single-point energies were compared to the results of the HSE06 hybrid functional with PW91 with optimized U parameter (set to 4 eV). The calculated values of the adsorption energies and the corresponding desorption temperatures (calculated from dΘ/dT versus T obtained from Langmuir model based on E_{ads} values) are presented in **Table S1**, and compared to the experiment.

Table S1: Comparison of PW91+U and HSE06 results for adsorption energy and corresponding desorption temperatures for selected Cu^+/6MR adsorbed species.*

		**-NO**	**$-NO_2^-$**	**$-NO_3^-$**
**Adsorption energy / eV**	**PW91+U**	-1.33	-2.42	-3.51
	**HSE06**	-0.77	-1.61	-2.83
**Calculated desorption temperature for $p_{NO}/p^0 = 10^{-4}$**	**PW91+U + 0.85 corr.**	230 °C	375 °C	450 °C
	**HSE06 + 0.85 corr.**	40 °C	180 °C	330 °C
	**HSE06**	25 °C	150 °C	295 °C
**Experimental SCO onset (TPSR)**		~150 °C		
**Experimental SCO cutoff (TPSR)**			~400 °C	
**Experimental stability (IR)**		~100-150 °C	~400 °C	~450 °C

*The 6MR has been selected for this benchmark calculation as it represents the majority of the occupied Cu sites in the studied zeolite, as evidenced by the IR study of NO adsorption and EPR measurements (see Materials and Methods section in the main manuscript).

Analysis of the data collated in **Table S1** indicates that apparently, PW91+U leads to better reproduction of the experimental desorption temperature than the nominally superior HSE06 functional. Interestingly, when the difference between PW91+U and HSE06 values decreases, the accuracy of the prediction of the experimental temperatures increases. This speaks in favor of the applied method for the temperature windows determination of the examined molecular events, using the dΘ/dT vs T profiles. Notably, the HSE06 locates the NO desorption peaks at very low temperatures (25–40 °C) where experimentally no changes are observed in the TPSR profiles. Considering the limitation of GGA+U, we are conscious that the absolute values of

DFT energies might be disputable. However, the selection of PW91+U (with U = 4 eV) for modeling CuSSZ-13 zeolite was based on a better agreement between the theoretical and experimental TPD and TPSR results, and is additionally fostered by its computational efficiency in comparison to the hybrid HSE06. Another argument for employing such calculation scheme is to be compatible with the previous seminal papers on the NO oxidation mechanism over CuSSZ-13 catalyst [31,32,33,34,35].

S3. Slab Model of the CuO (111) Surface

To investigate CuO nanocrystals segregated on zeolite sample i-CuSSZ-13, a slab model of the $Cu_{80}O_{80}$ stoichiometry, exposing (111) surface and shown in **Figure S3** was used.

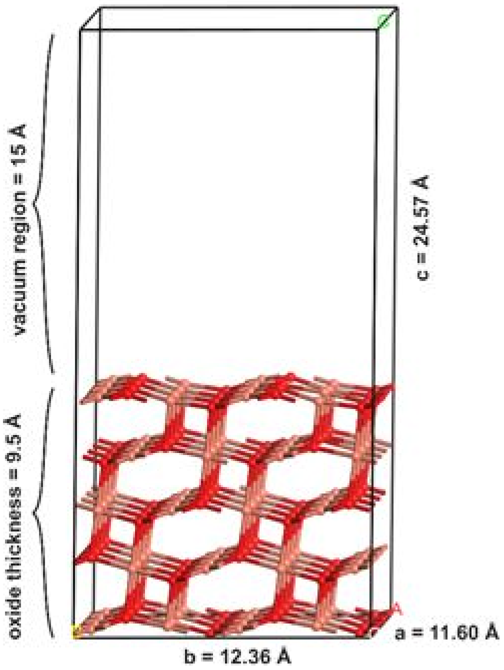

Figure S3. Slab model of the CuO crystal with the $Cu_{80}O_{80}$ stoichiometry exposing the (111) surface.

S4. Structure and Energetics of Copper Adducts with O_2, NO, NO_2, and NO_3

S4.1. NO and NO_x interaction with the isolated Cu sites

The optimized structures of copper active centers in SSZ-13 sites include single Cu^{2+} and Cu^{+} cations, hydroxo-copper species Cu^{2+}-$(OH)^{-}$, oxo-bridged di-copper species Cu^{2+}-O^{2-}-Cu^{2+}, peroxo-bridged di-copper Cu^{2+}-O_2^{2-}-Cu^{2+}, hydroxo-bridged di-copper Cu^{2+}-$(OH^{-})_2$-Cu^{2+}, copper cations in vicinal positions $Cu^{+}\cup Cu^{+}$. They are accommodated in the exchangeable 6-membered (6MR) and/or 8-membered ring (8MR) sites of the zeolite SSZ-13 (also referred to as sigma (**σ**) and tau (**τ**) sites, respectively [2]). The reference states, which are geometries of the optimized structures of bare CuSSZ-13 copper sites (without adspecies), their energies, and electronic parameters are described below.

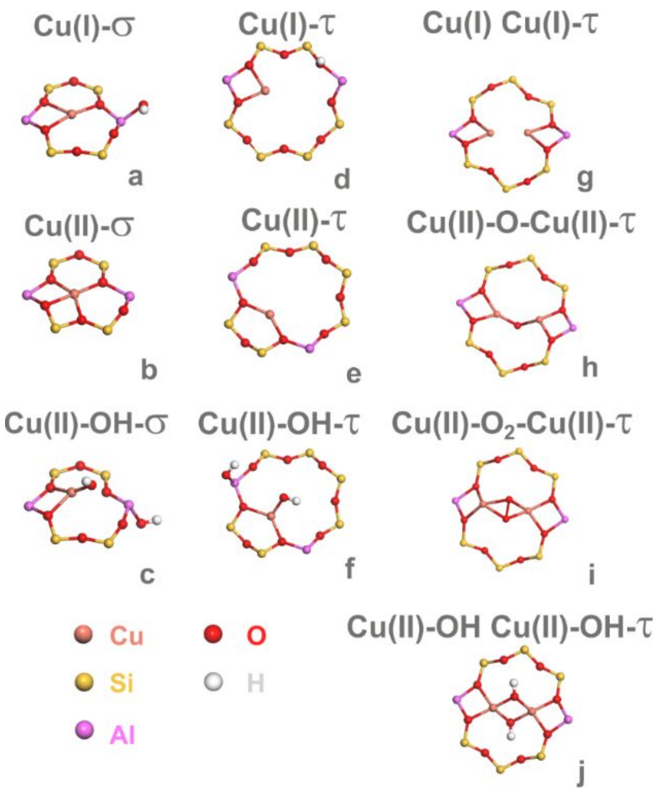

Figure S4. Models of bare copper active sites: single Cu in 6MR (**σ**) sites (**a-c**), single Cu in 8MR (**τ**) sites (**d-f**), and dimeric copper centers (**g-j**).

The optimized geometries and energetics of the considered single (Cu^{2+}, $Cu^{+}\cup H^{+}$, $Cu^{2+}OH^{-}$, Cu^{+}) and dual (μ–Cu^{2+}-O^{2-}-Cu^{2+}, μ–η:²η² Cu^{2+}-O_2^{2-}-Cu^{2+}, $Cu^{+}\cup Cu^{+}$, μ–η:²η² $Cu^{2+}(OH^{-})_2Cu^{2+}$) copper centers located in such sites are shown in **Figure S4**, and the most important bonding characteristics along with the electronic and magnetic properties are collated in **Table S2**. "∪" indicates two closely spaced copper centers with the Cu↔Cu distance of less than 0.4 nm.

Table S2. Selected electronic and magnetic properties of single Cu centers in the CuSSZ-13 zeolite.

Name	Electronic and magnetic state							
	Magnetization / μ_B				Δq_B / |e|			
	Cu_1	O_1	O_2	$O_{(OH)}$	Cu_1	O_1	O_2	$O_{(OH)}$
**Cu^{2+}/6MR**	0.63	0.07	0.05	-	0.84	-1.49	-1.50	-
**Cu^{+}/6MR**	0.00	0.00	0.00	-	0.66	-1.57	-1.55	-
**$Cu^{2+}OH^{-}$/6MR**	-0.65	-0.05	-0.08	-0.09	1.12	-1.50	-1.49	-1.26
**Cu^{2+}/8MR**	0.66	-1.51	-1.53	-	0.93	-1.49	-1.49	-
**Cu^{+}/8MR**	0.00	0.00	0.00	-	0.67	-1.53	-1.51	-
**$Cu^{2+}OH^{-}$/8MR**	0.56	0.02	0.06	0.25	1.02	-1.55	-1.49	-1.03

The interaction of NO, NO_2, and O_2 with single copper centers was investigated at first. The optimized geometries of the resultant adducts are shown in **Figure S5** and **Figure S6** while binding energies (with respect to the reference bare Cu centers) and partial charges are collated in **Tables S3–S5**.

Table S3. Adsorption energies of single Cu centers with NO_x species.

Name	n·NO	m·O_2	E^{ads} / eV	
			**6MR σ**	**8MR τ**
**$Cu^{+}NO$**	1	0	-1.33	-0.954
**$Cu^{2+}NO$**	1	0	-0.886	-1.44
**$[Cu^{2+}OH^{-}](NO)$**	1	0	-1.19	-1.05
**$Cu^{2+}NO_2^{-}$**	1	½	-2.43	-1.91
**$Cu^{2+}NO_3^{-}$**	1	1	-3.52	-2.76
**$Cu^{2+}NO_2^{\delta-}$**	1	½	-1.37	-1.79
**$Cu^{2+}NO_3^{\delta-}$**	1	1	-1.95	-2.36

Mixed hydroxyl and nitrates/nitrites were excluded from the present considerations due to their steric hindrance (with an extra OH group). For all species presented below, linkage isomerism such as η^1-N, η^1-O, η^2-(N,O), η^2-(O,O) was also considered.

NO ligand is attached in a η^1-N mode (ΔE^{ads} ranging from -0.89 to -1.44 eV), exhibiting a bent geometry (**Figure S5**). For the Cu^+NO adduct, the metal-to-ligand charge transfer is relatively low (Δq_{Cu} = 0.2-0.3), and the unpaired electron is essentially retained on the nitrosyl moiety. The spin alignment of the calculated nitrosyl and dinitrosyl species can be schematically described as:

Cu^+ + ↑NO → Cu^+–(↑)NO,

Cu^+ + 2↑NO → (↓NO)–Cu^+–(↑NO),

↑Cu^{2+} + ↑NO → Cu^{2+}(↑↓)NO,

↑Cu^{2+} + 2↑NO → NO(↑↓)Cu^{2+}–(↑NO),

[↑CuOH]$^+$ + ↑NO → [HOCu]$^+$(↑↓)NO.

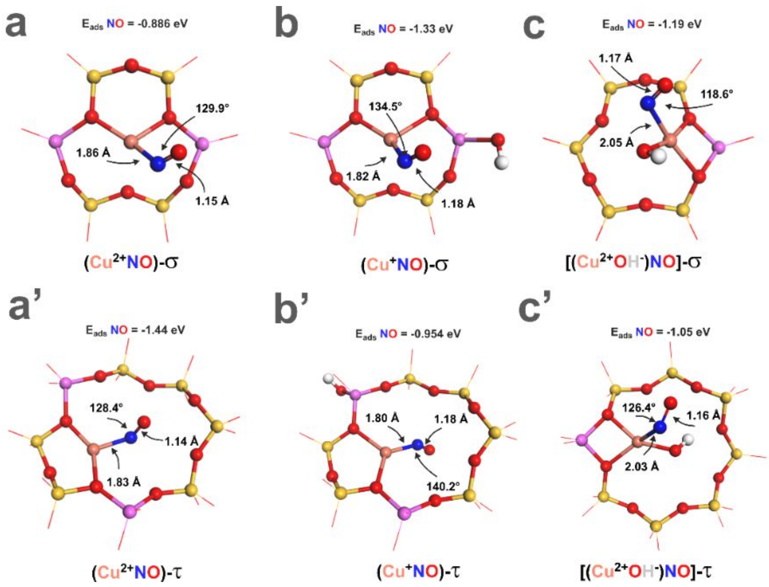

Figure S5. The most stable configurations of the NO molecule adsorbed on selected single Cu centers accommodated in the 6MR (σ) (**a**,**b**,**c**) and 8MR (τ) sites (**a'**,**b'**,**c'**).

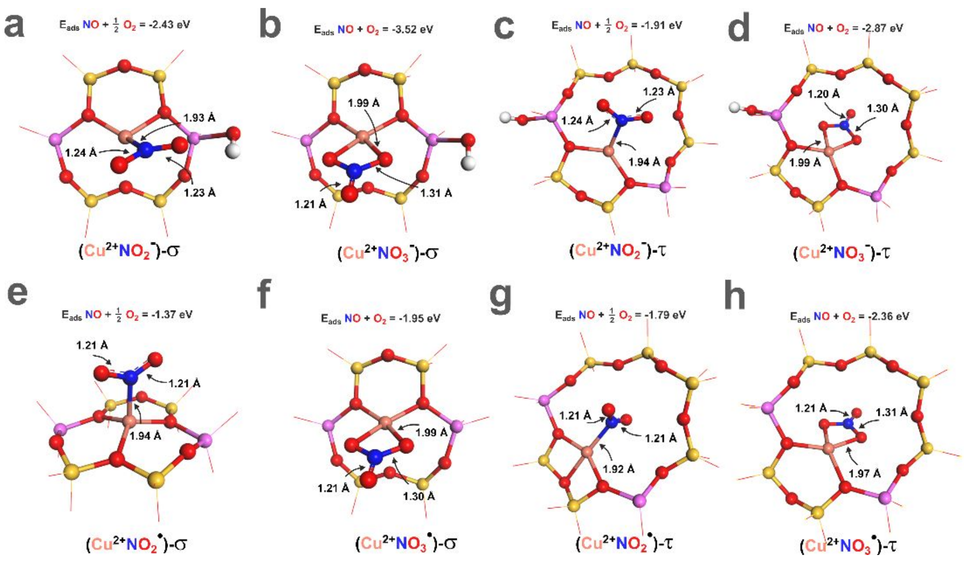

Figure S6. The most stable configurations of nitrites (NO_2^-) and nitrates (NO_3^-) for selected single Cu centers accommodated in the 6MR sites: (**a** – **d**) adducts with copper(I) centers, (**e** – **h**) adducts with copper(II) centers.

Table S4. Electronic and magnetic properties of NO_x adducts of single copper species accommodated in the 6MR rings (σ) of the CuSSZ-13 zeolite.

**6MR**	***Cu***		***Adspecies***					
			N		**O'** *(x2 for NOx)*		**O''** *(OH/ terminal)*	
	q_B	μ_B	q_B	μ_B	q_B	μ_B	q_B	μ_B
**Cu^+NO**	0.87	0.10	0.23	0.36	-0.40	0.29	-	-
**$Cu^{2+}NO$**	0.81	0.00	0.55	0.00	-0.33	0.00	-	-
**$[Cu^{2+}OH^-](NO)$**	0.93	0.00	0.52	0.00	-0.45	0.00	-0.90	0.00
**Cu^{2+}-(NO_2^-)**	0.94	0.42	0.51	0.11	-0.42	0.14	-	-
**Cu^{2+}-(NO_3^-)**	0.83	0.59	0.83	-0.09	-0.48	0.12	-0.37	0.04
**Cu^{2+}-($NO_2^\bullet$)**	0.87	0.22	0.56	0.03	-0.26	0.01	-	-
**Cu^{2+}-($NO_3^\bullet$)**	0.84	0.54	0.83	-0.05	-0.46	0.08	-0.33	0.01

In the presence of NO and O_2, nitrites and nitrates can be formed (**Figure S4)**. The optimized structures shown in **Figure S4a-d** (top row) are anionic NO_x^- adducts produced upon interaction with Cu^+, leading to the oxidation of copper cations and high stabilization energies up to 3.5 eV. The bottom row (**Figure S4e-h**) shows the structures produced upon interaction

with Cu^{2+}, which retain the radical nature of the ligands, exhibiting lower interaction energies (from 1.4 up to 2.4 eV).

The following equations schematically show reactions of NO_x formation (the charge and spin states are ascertained based on the population analysis; see **Table S4**):

$(\uparrow\downarrow)Cu^+ + \uparrow NO + \uparrow\uparrow O_2 \rightarrow \uparrow Cu^{2+}–NO_3^-$,

$(\uparrow\downarrow)Cu^+ + \uparrow NO + ½(\uparrow\uparrow O_2) \rightarrow \uparrow Cu^{2+}–NO_2^-$,

$(\uparrow\downarrow)Cu^+ + \uparrow NO_2 \rightarrow \uparrow Cu^{2+}–NO_2^-$,

$\uparrow Cu^{2+} + \uparrow NO + \uparrow\uparrow O_2 \rightarrow \uparrow Cu^{2+}–(\downarrow NO_3)$,

$\uparrow Cu^{2+} + \uparrow NO + ½(\uparrow\uparrow O_2) \rightarrow \uparrow Cu^{2+}–(\downarrow NO_2)$,

$\uparrow Cu^{2+} + \uparrow NO_2 \rightarrow \uparrow Cu^{2+}–(\downarrow NO_2)$.

Table S5. Electronic and magnetic properties of NO_x adducts of single copper centers accommodated in the 8MR rings (τ) of the CuSSZ-13 zeolite.

**8MR**	***Cu***		***Adspecies***					
			N		**O'**		**O''** *(OH/ terminal)*	
	q_B	μ_B	q_B	μ_B	q_B	μ_B	q_B	μ_B
**$Cu^+(NO)$**	0.95	0.084	0.24	0.37	-0.39	0.29	-	-
**$Cu^{2+}(NO)$**	0.94	0.00	0.58	0.00	-0.32	0.00	-	-
**$[Cu^{2+}OH^-]$-(NO)**	0.90	0.00	0.37	0.00	-0.43	0.00	-0.91	0.00
**$Cu^{2+}(NO_2^-)$**	0.84	-0.37	0.52	-0.12	-0.39	-0.15	-	-
**$Cu^{2+}(NO_3^-)$**	0.88	0.56	0.85	-0.01	-0.46	0.14	-0.35	0.04
**$Cu^{2+}(NO_2^{\delta-})$**	0.83	0.00	0.59	0.00	-0.26	0.00	-	-
**$Cu^{2+}(NO_3^{\delta-})$**	0.90	-0.56	0.82	0.01	-0.47	-0.12	-0.33	-0.03

S4.2. Dual Cu centers in CuSSZ-13 under real conditions (T, p(O_2))

In order to obtain a correct description of the active centers of the zeolite catalyst under real conditions, the behavior of the dual-Cu active centers in contact with gaseous O_2 and H_2O was simulated using atomistic thermodynamics. A zeolite unit cell with the highlighted 8MR ring containing two parent adjacent copper cations is shown in **Figure S7a,b$_1$**, whereas the geometries of the corresponding oxo and peroxo dimer centers in **Figure S7b$_2$-b$_3$** (in an epitomic form).

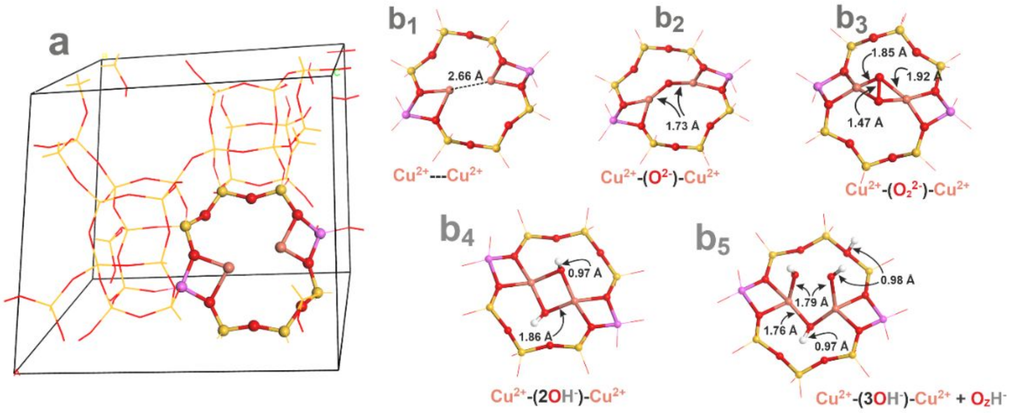

Figure S7. The CuSSZ-13 unit cell with the highlighted 8MR site containing two adjacent copper cations (**a**) and epitomes of dual-Cu centers without oxygen (**b$_1$**), containing oxo bridge (**b$_2$**) and peroxo bridge (**b$_3$**), together with the structure of the hydrated (**b$_4$**) and doubly hydrated (**b$_5$**) Cu^{2+}-O^{2-}-Cu^{2+} site.

A detailed analysis of the quantum-chemical calculation results shows that in the optimal geometry, the Cu–Cu distance for the bare dual center is 2.66 Å (see **Figure 75b$_1$**). Both atoms are in the reduced (Cu^+) state, characterized by the zero magnetic moment and Bader charge of ~0.7|e| (the electronic and magnetic properties of the models are summarized in **Table S6**). In the case of Cu^{2+}-O^{2-}-Cu^{2+} centers (produced formally by oxygen atom adsorption), the stabilization energy (calculated in relation to ½O_2) is –1.895 eV. The Cu–O bond length is ~1.73 Å. None-zero magnetic moments with an apparent increase in the partial charge on copper atoms (see **Table S6**) confirm the oxidation of copper cores to the Cu^{2+} forms. The

bridging oxygen atom adopts the O^{2-} state featured by the zero magnetic moment and the Bader charge equal to –1.345|e|, see **Table S6**). A comparison of the energy of the antiferromagnetic and ferromagnetic configurations of the Cu-O-Cu bridges indicates that the former is more stable, and the Cu cations are characterized by magnetic moments of –0.526 and 0.526 μ_B.

Adsorption of O_2 molecule on the dual copper center leads to the structure shown in **Figure S7b$_3$**, characterized by the formation energy of –2.083 eV. Analysis of the electronic structure and the geometry of Cu-(O_2)-Cu bridge (**Table S6**) indicates formation of the $Cu^{2+}O_2^{2-}Cu^{2+}$ species ($\mu_{(O2)}$ = 0.01 μ_B, $\Delta q_B(O_2)$ = –1.69|e|, $d_{O\text{-}O}$ = 1.45 Å).

Table S6. Selected properties of dual Cu centers in CuSSZ-13 zeolite. E^{ads} is calculated with respect to the number of adsorbed oxygen atoms ($n\cdot$½O_2).

**Active center**	***n*·O**	**E^{ads} / eV**	***Electronic and magnetic state***					
			Magnetization / μ_B			***Δq_B* / |e|**		
			Cu_1	*Cu_2*	*O_x*	*Cu_1*	*Cu_2*	*O_x*
Cu–Cu	0	0.00	0.000	0.000	–	0.668	0.641	–
$Cu^{2+}O^{2-}Cu^{2+}$	1	–1.81	–0.526	0.526	0.066	1.012	1.015	–1.345
$Cu^{2+}O_2^{2-}Cu^{2+}$	2	–2.08	–0.506	–0.498	0.011	1.034	1.024	–0.412

To simulate contact with water molecules, the adsorption of one and two H_2O molecules was modeled for the thermodynamically most stable $Cu^{2+}O^{2-}Cu^{2+}$ site. Associative and dissociative adsorption modes were tested with the possibility of stabilization of the detached proton on the bridging oxygen and/or zeolite lattice. The most stable geometries are shown in **Figure S7b$_4$-b$_5$** (together with the selected geometrical features), whereas the corresponding electronic properties are summarized in **Table S7**.

The structure of the η^2-O $Cu^{2+}(OH^-)_2Cu^{2+}$ species is shown in **Figure S7b$_4$**. The presence of –OH groups leads to the quenching of the antiferromagnetic spin configuration (see **Table S7**). The second water molecule dissociates upon attachment, resulting in an adduct of the stoichiometry of $Cu^{2+}(OH^-)_3Cu^{2+}$ (**Figure S7b$_5$**), with the proton stabilized by the zeolite lattice.

Table S7. Selected properties of copper hydroxo adducts obtained upon the interaction of $n\cdot½O_2$ and $n\cdot H_2O$ molecules with dual copper centers of the CuSSZ-13 zeolite.

Active center	$n\cdot½O_2$	$n\cdot H_2O$	E^{ads} / eV	***Dimer state***			
				μ_B		Δq_B	
				Cu_1	Cu_2	Cu_1	Cu_2
**$Cu^{2+}(OH^-)_2Cu^{2+}$**	1	1	–2.97	0.531	0.532	1.011	1.003
**$Cu^{2+}(OH^-)_3Cu^{2+}$**	1	2	–3.41	0.526	0.526	0.998	1.051

Based on the energies summarized in **Tables S6-S7**, *ab initio* thermodynamic modeling was used to assess the relative stability of the dual sites in dry and wet conditions (**Figure S8**). For the dry conditions (absence of water), only the oxygen-containing dual sites were taken into account (**Figure S7b$_1$-b$_3$**), and their relative thermodynamic stability was plotted against oxygen pressure and temperature (**Figure S8a**). As can be seen, the $Cu^{2+}O^{2-}Cu^{2+}$ sites are the most stable for pressures and temperatures typical of catalytic processes. They dominate between 0 and 600 °C in the assumed pressure range. Under the low-temperature conditions, the appearance of the $Cu^{2+}O_2^{2-}Cu^{2+}$ entities is more likely. At the same time, bare $Cu^+\cup Cu^+$ dual centers are expected only under highly reducing conditions of high temperature and shallow oxygen pressure. For a typical oxygen pressure (p_{O2} = 0.01 atm), the course of the free enthalpy line as a function of T (**Figure S8b**) shows that the investigated $Cu^{2+}O^{2-}Cu^{2+}$, $Cu^{2+}O_2^{2-}Cu^{2+}$, and $Cu^+\cup Cu^+$ species are relatively well separated in terms of energy. Their coexistence is limited to the region of intersection of the ΔG planes. The less stable $Cu^{2+}O_2^{2-}Cu^{2+}$ entity constitutes an intermediate of the O_2 activation, whereas $Cu^+\cup Cu^+$ may appear due to the evacuation of a CuSSZ-13 sample at high temperatures.

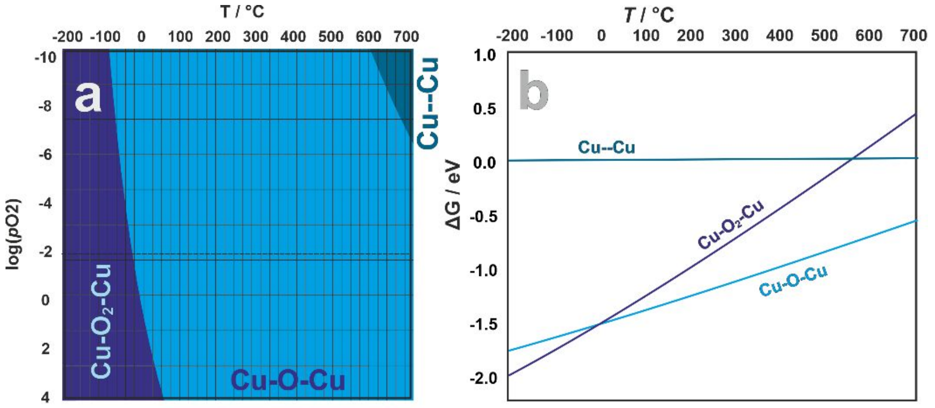

Figure S8. Thermodynamic diagram describing the relative stability of the dual Cu centers as a function of T and p_{O2} (**a**) and its intersection for p_{O2} set to 0.01 atm (**b**) (p_{O2} set to 0.03 atm).

S4.3. Interaction of NO and NO$_x$ with dual Cu sites

Interaction of NO and NO/O_2 with $Cu^{+}\cup Cu^{+}$, $Cu^{2+}O^{2-}Cu^{2+}$, $Cu^{2+}O_2^{2-}Cu^{2+}$ and $Cu^{2+}(OH^{-})_2Cu^{2+}$ was modeled, taking into account many possible configurations of the resulting adducts. We tested configurations in which O and NO remained separated and those in which ON–O bonds were formed between admolecules, as well as the attachment of NO directly to the bridging oxygen of the $Cu^{2+}O^{2-}Cu^{2+}$ and $Cu^{2+}O_2^{2-}Cu^{2+}$ centers. The preliminary results indicated that NO adsorption via nitrogen atom was much more favorable than via oxygen (the adsorption energy difference reached 0.67 eV), and only such adsorption modes were considered. The most stable configurations showing the addition of one NO molecule to the dual Cu centers are shown in **Figure S9a-d**, whereas the energetics and electronic structure parameters are summarized in **Tables S8-S9**.

The adsorption of NO molecule on the $Cu^{+}\cup Cu^{+}$ center is shown in **Figure S9a**. The NO molecule binds through nitrogen in a symmetric configuration, forming a nitrosyl bridge with two equal Cu–N bonds (1.81 Å). The calculated adsorption energy is ΔE_{ads} = -2.487 eV. The charge transfer to NO is small (Δq = 0.18), and the Cu cations retain their original oxidation state (Cu^{+}). In the case of $Cu^{2+}O^{2-}Cu^{2+}$ (**Figure S9b**), NO adsorption can be realized by binding to one of the copper cations (η^1-N) with an interaction energy of -2.67 eV (calculated with

respect to NO, ½O_2, and $Cu^+ \cup Cu^+$). A slight charge transfer from NO, slightly quenched magnetic moments of only one Cu and NO, and negligible magnetic moment on the bridge oxygen (**Table S9**), corresponding to the $NO^{\delta+}$-$Cu^{2+}O^{2-}Cu^{2+}$ Species. The properties of NO attached to the $Cu^{2+}O_2^{2-}Cu^{2+}$ center are similar (**Figure S9c**). The peroxide moiety maintains the O_2^{2-} diamagnetic state, while the copper cations remain oxidized, but the magnetic moment of the Cu cation that interacts with NO is quenched. The resultant adduct can be formulated as NO-$Cu^{2+}(O_2^{2-})Cu^{2+}$, with the interaction energy of -1.929 eV. In the case of NO adduct with the dual hydroxylated Cu centers (**Figure S9d**), the copper cores become antiferromagnetically coupled, while the magnetic moment was retained on the NO moiety. This corresponds to $NO^{\delta+}$-$Cu^{2+}(OH^-)_2Cu^{2+}$ (ΔE_{ads} = -3.536 eV) with slight electron transfer from NO to the metal.

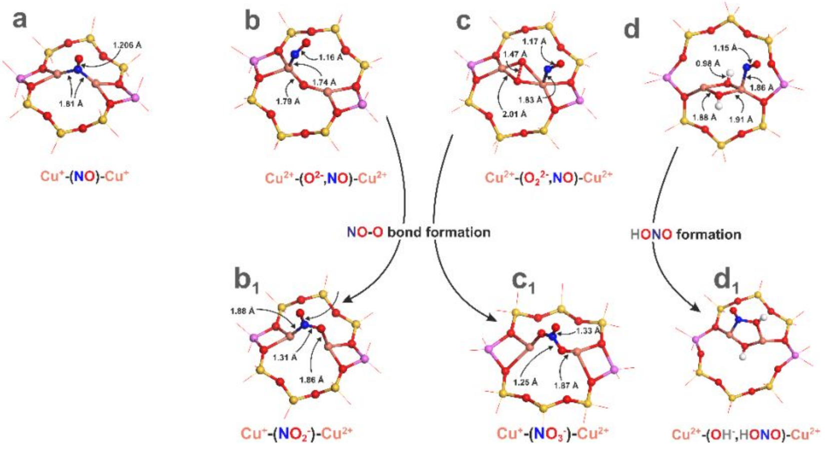

Figure S9. The most stable configurations of NO molecule adsorbed on selected dual Cu centers in the 8MR sites of SSZ-13 zeolite.

Table S8: Stabilization energy of NO_x adducts with dual Cu sites of the SSZ-13 zeolite.

Structure	n·NO	n·O	n·H_2O	E^{ads}
**$Cu^+(NO)Cu^+$**	1	0	0	–2.49
**$Cu^{2+}(O^{2-},NO)Cu^{2+}$**	1	1	0	–2.67
**$Cu^{2+}(O_2^{2-},NO)Cu^{2+}$**	1	2	0	–1.93
**$Cu^+(NO_2^-)Cu^{2+}$**	1	1	0	–3.78
**$Cu^+(NO_3^-)Cu^{2+}$**	1	2	0	–2.92
**$Cu^{2+}((OH^-)_2,NO)Cu^{2+}$**	1	1	1	–3.14

Table S9: Electronic and magnetic properties of NOx and HONO adducts with dual Cu centers of the CuSSZ-13 zeolite.

Structure	*Cu-dual center*				*Adspecies*			
	Cu_1		*Cu_2*		NO_x		O_x	
	q_B	μ_B	q_B	μ_B	q_B	μ_B	q_B	μ_B
**$Cu^+(NO)Cu^+$**	0.81	0.13	0.80	0.12	–0.18	0.53	--	--
**$Cu^{2+}(O^{2-},NO)Cu^{2+}$**	0.79	0.31	0.75	0.55	0.12	0.38	–0.77	–0.01
**$Cu^{2+}(O_2^{2-},NO)Cu^{2+}$**	0.98	0.35	0.83	0.04	0.02	–0.43	–1.01	0.01
**$Cu^{2+}((OH^-)_2,NO)Cu^{2+}$**	1.01	–0.49	0.99	0.45	0.12	0.38	--	--
**$Cu^+(NO_2^-)Cu^{2+}$**	0.93	0.57	0.71	0.13	–0.42	0.03	--	--
**$Cu^+(NO_3^-)Cu^{2+}$**	0.99	0.45	0.82	0.19	–0.55	-0.02	--	--
**$Cu^+(OH^-,HONO)Cu^{2+}$**	0.89	0.47	0.56	0.02	–0.01	0.00	--	--

In all described cases, NO binds to Cu through the nitrogen atom, and there is no pronounced molecule activation (low charge transfer, largely preserved magnetic moment, and no significant modification of the N=O bond length). However, new thermodynamically stable species are produced for adducts with the bridging oxygen (**Figure S9b,c,d**). For the $Cu^{2+}O^{2-}Cu^{2+}$ centers, NO can combine with the oxo moiety to form NO_2 (**Figure S9b$_1$**). Such a reorganization of bonds leads to the additional stabilization by 1.1 eV. The resultant NO_2^- moiety is negatively charged at the expense of the bridge oxygen, with a concomitant reduction of one of the copper cores (the asymmetric charge and magnetic moment distribution of both Cu cores are shown in **Table S9**). Such adducts can be denoted as $Cu^+(NO_2^-)Cu^{2+}$, where NO_2^- binds in μ-η^1:η^1(N,O) mode. In the case of the attachment of NO to the peroxy moiety (**Figure S9c$_1$**), a NO_3^- anion stabilized on the Cu^+/Cu^{2+} pair of copper cations is formed analogously. The resultant $Cu^+(NO_3^-)Cu^{2+}$ adduct is more stable than the initial one (by 0.95 eV), and the binding mode is μ-η^1:η^1(O,O). In the case of dual hydroxylated Cu centers, the interaction between NO and one of the OH^- groups gives rise to a bound HONO species (μ-η^1:η^1(N,O)), which is stabilized on both copper cores (**Figure S9d$_1$**). Contrary to the previously

described nitrite and nitrate adducts, this complex is less stable (by 0.70 eV), and its electronic structure may be formulated as $Cu^+(OH^-,HONO)Cu^{2+}$ (see **Table S9**).

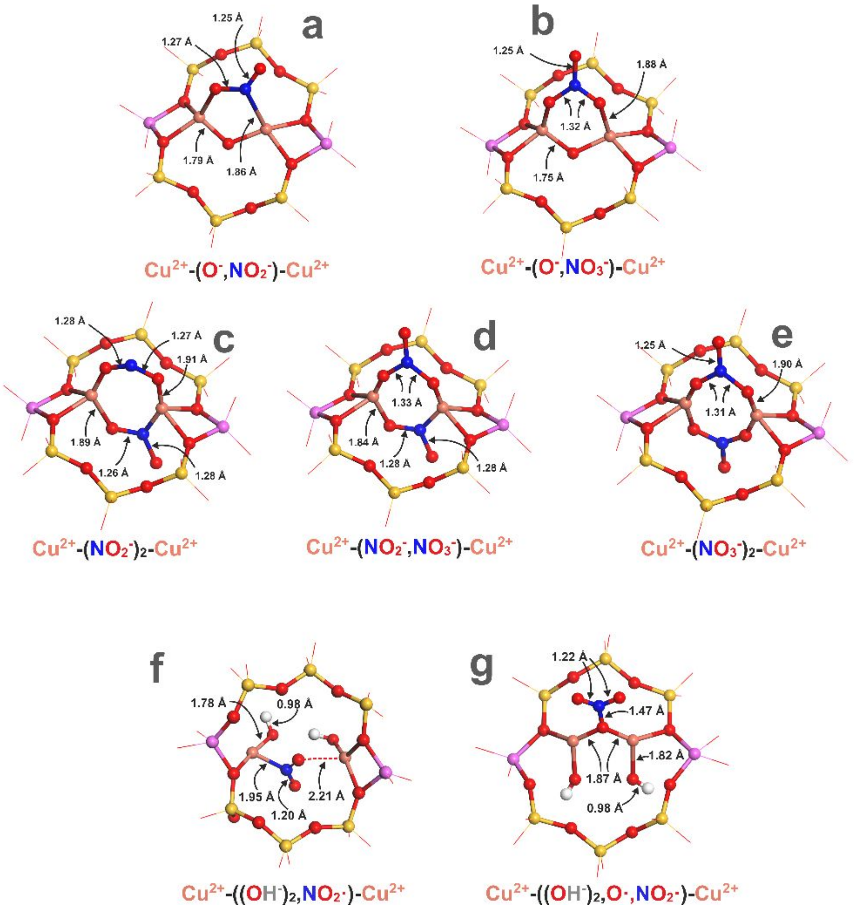

Figure S10. The most stable configurations of NO_x/O species on dual copper centers accommodated in the 8MR sites of the SSZ-13 zeolite.

When more NO and O_2 molecules are co-adsorbed, further structures involving nitrate/nitrite species can be proposed. Various combinations of NO_x and O/O_2 fragments lead to the configurations shown in **Figure S10** (with the stoichiometry and energetics summarized in **Table S10**). The co-adsorption of NO_2 and O (corresponding formally to the adsorption of NO and O on the bare $Cu^+\cup Cu^+$ center) is shown in **Figure S10a**. With the interaction energy of E^{ads} = –3.46 eV, the co-adsorbed fragments are located on the opposite sides of the copper

center, connecting with both copper cores in bridging configurations. Analysis of the electronic properties (**Table S11**) shows that this species can be formulated as $Cu^{2+}(O^{-},NO_3^{-})Cu^{2+}$.

For the co-adsorption of NO_3 and O (**Figure S10b**), the geometry of the system and the distribution of charges show significant similarities to the previous case. The bridging oxygen (O^{-}) and the nitrate ion (NO_3^{-}) are positioned on the opposite side of the Cu^{2+} cores. The next three structures show the co-adsorption of two NO_x-type fragments. The first one (**Figure S10c**) shows the simultaneous attachment of two NO_2 groups located in opposite sites.

Table S10. Stoichiometry and stabilization energy of NO_x adsorbed species on dual Cu centers in the CuSSZ-13 zeolite.

Structure	n·NO	n·O	n·H_2O	ΔE_{ads}
$Cu^{2+}(O^{-},NO_2^{-})Cu^{2+}$	1	1	0	–1.75
$Cu^{2+}(O^{-},NO_3^{-})Cu^{2+}$	1	2	0	–2.23
$Cu^{2+}(NO_2^{-})_2Cu^{2+}$	2	2	0	–4.56
$Cu^{2+}(NO_2^{-},NO_3^{-})Cu^{2+}$	2	3	0	–4.66
$Cu^{2+}(NO_3^{-})_2Cu^{2+}$	2	4	0	–6.33
$Cu^{2+}((OH^{-})_2,NO_2)Cu^{2+}$	1	2	1	–3.19
$Cu^{2+}((OH^{-})_2,O,NO_2)Cu^{2+}$	1	3	1	–3.40

Table S11. Electronic and magnetic properties of NO_x adsorbed species stabilized on dual Cu centers in CuSSZ-13 zeolite.

Structure	*Cu-dual-site*				*Adspecies*			
	Cu_1		Cu_2		NO_x		O_x	
	q_B	μ_B	q_B	μ_B	q_B	μ_B	q_B	μ_B
$Cu^{2+}(O^{-},NO_2^{-})Cu^{2+}$	0.951	–0.333	0.949	0.454	–0.497	0.00	–0.52	–0.27
$Cu^{2+}(O^{-},NO_3^{-})Cu^{2+}$	0.951	–0.333	0.949	0.454	–0.481	0.00	–0.44	–0.31
$Cu^{2+}(NO_2^{-})_2Cu^{2+}$	0.951	–0.333	0.949	0.454	–0.452	0.00	-	-
$Cu^{2+}(NO_3^{-})_2Cu^{2+}$	1.002	–0.425	0.908	0.501	–0.531	0.00	-	-
$Cu^{2+}(NO_2^{-},NO_3^{-})Cu^{2+}$	0.912	0.390	1.023	–0.505	–0.531	0.00	-	-
$Cu^{2+}((OH^{-})_2,NO_2)Cu^{2+}$	0.951	0.421	0.988	–0.498	0.123	0.39	-	-
$Cu^{2+}((OH^{-})_2,NO_3)Cu^{2+}$	0.925	0.467	1.040	–0.462	0.092	0.42	-	-

The population analysis (**Tables S10-S11)** shows that the resultant can be described as $Cu^{2+}(NO_2^-)_2Cu^{2+}$) with the stabilization energy of –4.56 eV. The structure (**Figure S10d)** represents a $Cu^{2+}(NO_2^-,NO_3^-)Cu^{2+}$) adduct and is characterized by E_{ads} of –4.66 eV, whereas (**Figure S10e**) corresponds to $Cu^{2+}(NO_3^-)_2Cu^{2+}$ with the stabilization energy of –6.33 eV. The last two states describe the adsorption of NO_2 and NO_3 (**Figure S8f** and **Figure S8g**, respectively) on the $Cu^{2+}(OH)_2Cu^{2+}$ centers. In both cases, unlike before, NO_x is added without electron transfer, and finally, the NO_2·or NO_3·retain their radical character (see **Tables S10-S11**). Therefore, this adsorption type is energetically less effective (ΔE^{ads} = –3.19 and –3.40 eV for NO_2 and NO_3, respectively), manifested by low thermodynamic stability of the respective adducts.

S5. Effect of HONO Pressure

The proposed pathways or copper-redox oxidation of NO involve several reactions between the $Cu^{2+}OH^{-}$ species generating HONO intermediate. A virtual value of the cage HONO pressure has been estimated for the FPT modeling. Its maximum level cannot exceed the input pressure of NO (500 ppm = $5 \cdot 10^{-4}$ atm), and given that hydroxyl centers do not contribute more than 5-10% of total copper sites in our samples, we assumed that the maximum amount of HONO should be lower than 10^{-5}.

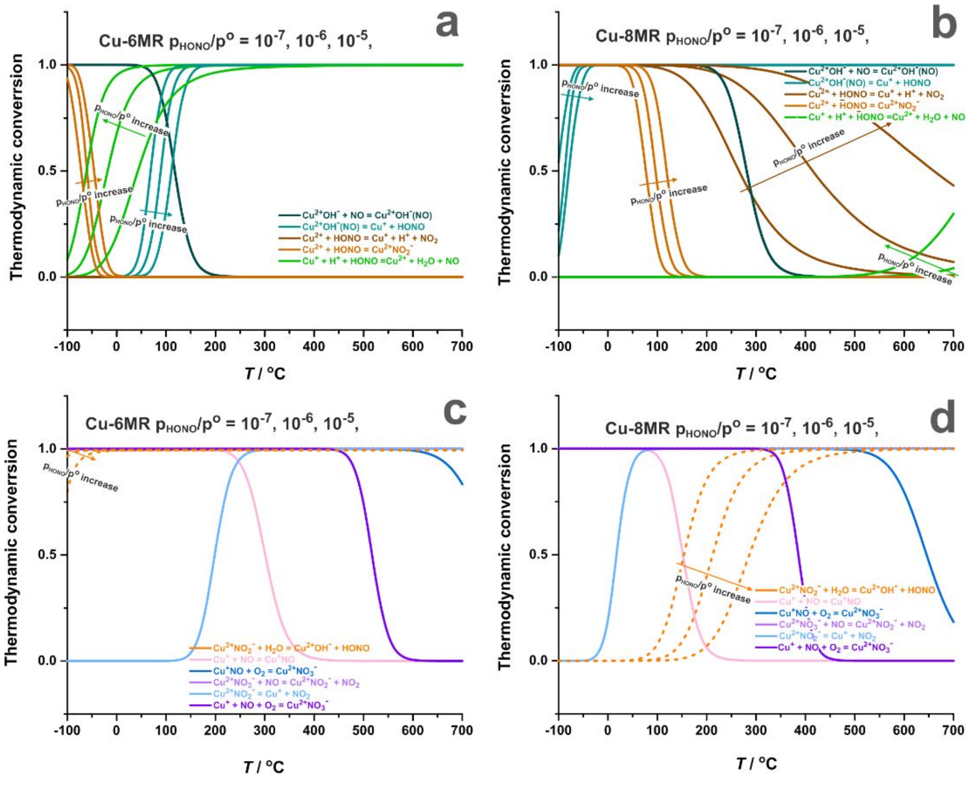

Figure S11. Influence of partial pressure of HONO on thermodynamic conversion plots for Cu located in 6MR (**a**, **c**) and 8MR (**b**, **d**), in the Cu redox cycles (**a**, **b**) and ligand redox oxidation (**c**, **d**).

A series of decreasing pressures of HONO was applied in the thermodynamic models (**Figure S11**) down to 10^{-7} atm, which approaches the limits of QMS detection. The results show that changes in the HONO pressure in this interval shift the temperature of most of the

investigated processes within 50 ^oC (**Figure S11a,b**). Stronger influence was observed only for copper oxidation reaction in 6MR ($Cu^+ + HONO + H^+ = Cu^{2+} + NO + H_2O$, **Figure S11a**) and copper reduction in 8MR ($Cu^{2+} + HONO = Cu^+ + H^+ + NO_2$, **Figure S11b**). Nitrite hydrolysis (**Figure S11c,d**) is only affected for 8MR (**Figure S11d**) by HONO pressure; the reaction threshold moves from 150 ^oC to 250 ^oC. Auxiliary catalytic tests in the presence of water show small NO to NO_2 conversion above 200 °C, matching the HONO pressure of 10^{-6}, which was then selected for all FPT modeling.

S6. Reactivity of CuO Nanocrystals with NO

Structure of the (111)CuO surface in various states

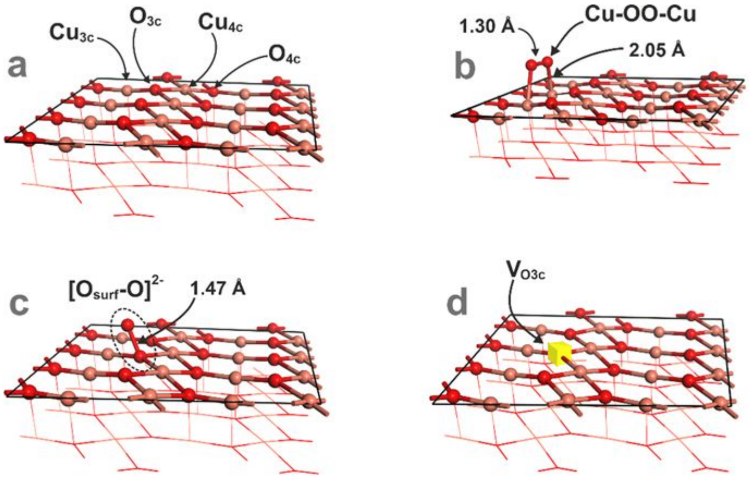

Figure S12. Stoichiometric CuO(111) surface, together with its oxidized state upon covering with diatomic (**b**) and monoatomic (**b**) oxygen adspecies, and reduced state produced by oxygen vacancy formation (**d**).

NO_x adspecies on the (111) CuO surface

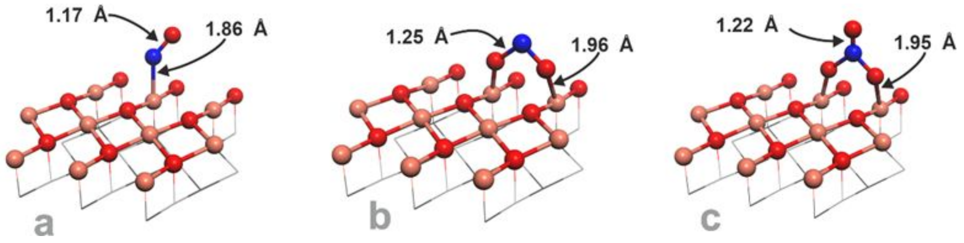

Figure S13. The most stable configurations of NO (a) $NO_2^{\delta-}$ (b) and $NO_3^{\delta-}$ (c) adspecies on the (111) CuO surface.

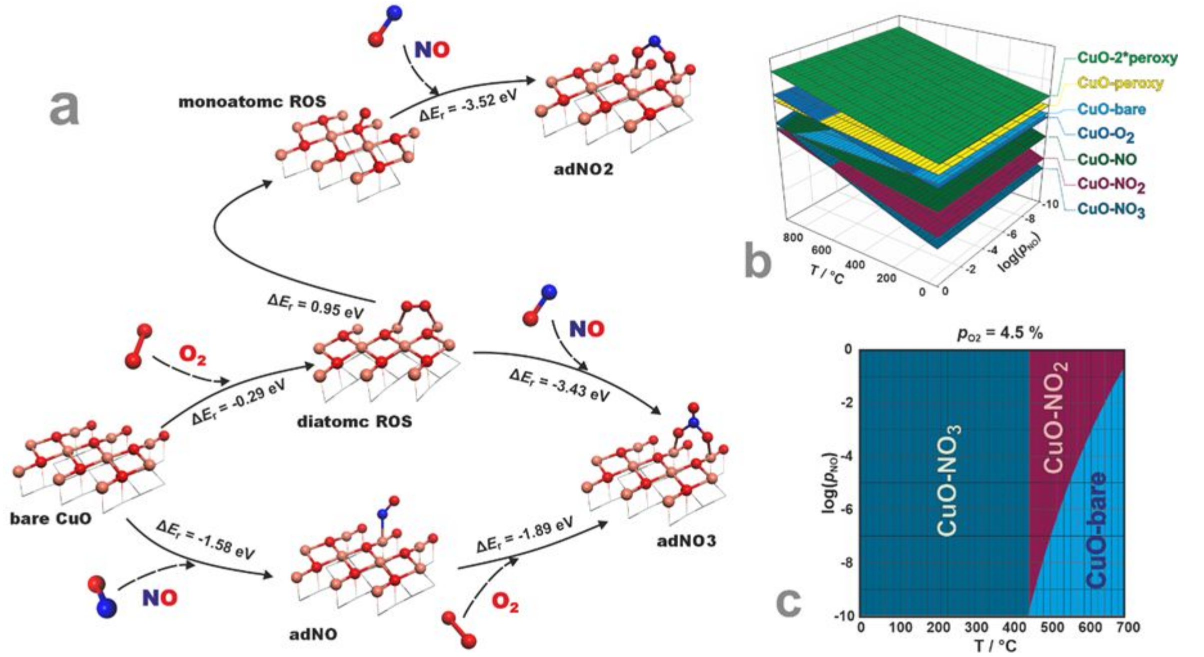

Figure S14. The molecular picture of possible pathways of NO_x formation on the (111) CuO surface (**a**) together with the corresponding FPT 3D diagram, representing the most important surface states in perspective view (**b**) and its bottom 2D projection (**c**).

The slab models representing stoichiometric, oxidized (via ROS formation), and reduced (via V_O formation) (111) surface of CuO are presented in **Figure S12**, whereas the most stable configurations of NO, $NO_2^{\delta-}$, and $NO_3^{\delta-}$adspecies are shown in **Figure S13**. These models were then used to study the molecular pathways of the NO, $NO_2^{\delta-}$ and $NO_3^{\delta-}$ adspecies development (**Figure S14**). Their calculated formation energies, equal to –1.58, –3.05, and –3.47 eV, respectively (see **Table 4**), indicate a robust thermodynamic driving force for their formation. Also, the $NO_2^{\delta-}$ and $NO_3^{\delta-}$ adduct formation on the oxidized surface (ΔE_r = –3.52 and –4.78 eV, respectively) is thermodynamically favored. The stability of the (111) CuO surface differently covered by NO/O_2 was evaluated using the calculated phase diagram, $\Delta G(T,p_{NO})$, with p_{O2} set to 4.5%. Its perspective view (**Figure S14b**) reveals that the surface covered separately with oxygen or NO adspecies is generally less stable than the surface containing the $NO_2^{\delta-}$ and $NO_3^{\delta-}$ adducts. The 2D diagram in **Figure S14c** shows that in a low-temperature regime, surface tends

to be covered with the $NO_3^{\delta-}$ adspecies. With the growing temperature, the $NO_2^{\delta-}$ adspecies become more stable than $NO_3^{\delta-}$, and the borderline of this process appears at ~430 °C. The thermal stability of $NO_2^{\delta-}$ depends on p_{NO}, at the high-temperature side, and for the highest considered pressures of NO (1 atm), the $NO_2^{\delta-}$ adducts are stable up to 700 °C, whereas at the lowest p_{NO} they decompose back to NO and O_2 above ~450 °C.

REFERENCES

[1] Dedecek, J.; Sobalik, Z.; Wichterlova, B. Siting and Distribution of Framework Aluminium Atoms in Silicon-Rich Zeolites and Impact on Catalysis. *Catal. Rev.: Sci. Eng.* **2012**, *54*, 135−223.

[2] K. Mlekodaj, J. Dedecek, V. Pashkova, E. Tabor, P. Klein, M. Urbanova, R. Karcz, P. Sazama, S. R. Whittleton, H. M. Thomas, A. V. Fishchuk, S. Sklenak, Al Organization in the SSZ-13 Zeolite. Al Distribution and Extraframework Sites of Divalent Cations. *J. Phys. Chem. C* **2019**, *123*, 7968–7987.

[3] Sazama, P.; Tabor, E.; Klein, P.; Wichterlova, B.; Sklenak, S.; Mokrzycki, L.; Pashkkova, V.; Ogura, M.; Dedecek, J. Al-Rich Beta Zeolites. Distribution of Al Atoms in the Framework and Related Protonic and Metal-Ion Species. *J. Catal.* **2016**, *333*, 102−114.

[4] Sklenak, S.; Andrikopoulos, P. C.; Whittleton, S. R.; Jirglova, H.; Sazama, P.; Benco, L.; Bucko, T.; Hafner, J.; Sobalik, Z. Effect of the Al Siting on the Structure of Co(II) and Cu(II)

Cationic Sites in Ferrierite. A Periodic DFT Molecular Dynamics and FTIR Study. *J. Phys. Chem. C* 2013, *117*, 3958−3968.

[5] Sklenak, S.; Andrikopoulos, P. C.; Boekfa, B.; Jansang, B.; Novakova, J.; Benco, L.; Bucko, T.; Hafner, J.; Dedecek, J.; Sobalik, Z. N_2O Decomposition over Fe-Zeolites: Structure of the Active Sites and the Origin of the Distinct Reactivity of Fe-Ferrierite, Fe-ZSM-5, and Fe-Beta. A Combined Periodic DFT and Multispectral Study. *J. Catal.* 2010, *272*, 262−274.

[6] Sobalik, Z.; Sazama, P.; Dedecek, J.; Wichterlova, B. Critical Evaluation of the Role of the Distribution of Al Atoms in the Framework for the Activity of Metallo-Zeolites in Redox N_2O/NO_x Reactions. *Appl. Catal., A* 2014, *474*, 178−185.

[7] Sazama, P.; Wichterlova, B.; Sklenak, S.; Parvulescu, V. I.; Candu, N.; Sadovska, G.; Dedecek, J.; Klein, P.; Pashkova, V.; Stastny, P. Acid and Redox Activity of Template-Free Al-Rich H-BEA and Fe-BEA Zeolites. *J. Catal.* 2014, *318*, 22−33.

[8] Meeprasert, J.; Kungwan, N.; Jungsuttiwong, S.; Namuangruk, S. Location and reactivity of extra-framework cation in the alkali exchanged LTL zeolites: A periodic density functional study. *Microporous Mesoporous Mater.* 2014, *195*, 227–239.

[9] Jajko, G., Kozyra, P., Strzempek, M., Indyka, P., Zając, M., Witkowski, S., Piskorz, W. Structural studies of aluminated form of zeolites – EXAFS and XRD experiment, STEM micrography, and DFT modelling. *Molecules*, 2021, *26*, 3566.

[10] Pérez, E.; Dubbeldam, D.; Liu, B.; Smit, B.; Calero, S. A computational method to characterize framework aluminum in aluminosilicates. *Angew. Chem. Int. Ed.* **2007**, *46*, 276–278.

[11] Di Iorio, J.R.; Gounder, R. Controlling the Isolation and Pairing of Aluminum in Chabazite Zeolites Using Mixtures of Organic and Inorganic Structure-Directing Agents. *Chem. Mater.* **2016**, *28*, 2236–2247.

[12] Majda, D.; Paz, F.A.; Friedrichs, D.; Foster, M.D.; Simperler, A.; Bell, R.G.; Klinowski, J. Hypothetical zeolitic frameworks: In search of potential heterogeneous catalysts. *J. Phys. Chem. C* **2008**, *112*, 1040–1047.

[13] Bickel, E. E.; Nimlos, C. T.; Gounder, R. Developing quantitative synthesis-structure-function relations for framework aluminum arrangement effects in zeolite acid catalysis. *J. Catal.* **2021**, *399*, 75–85.

[14] Bae, J.; Dusselier, M. Synthesis strategies to control the Al distribution in zeolites: thermodynamic and kinetic aspects. *Chem. Commun.* **2023**, *59*, 852–867.

[15] Li, S.; Li, H.; Gounder, R.; Debellis, A.; Müller, I. B.; Prasad, S.; Moini, A.; Schneider, W. F. First-Principles Comparison of Proton and Divalent Copper Cation Exchange Energy Landscapes in SSZ-13 Zeolite. *J. Phys. Chem. C* **2018**, *122*, 23564−23573.

[16] Wijerathne, A.; Sawyer, A.; Daya, R.; Paolucci, C. Competition between Mononuclear and Binuclear Copper Sites across Different Zeolite Topologies. *JACS Au* **2024**, *4*, 197–215.

[17] Löwenstein, W. The Distribution of Aluminum in the Tetrahedra of Silicates and Aluminates. *American Mineralogist* **1954**, *39*, 92396.

[18] Moreno-González, M.; Millán, R.; Concepción, P.; Blasco, T.; Boronat, M. Spectroscopic Evidence and Density Functional Theory (DFT) Analysis of Low-Temperature Oxidation of Cu^{+} to $Cu^{2+}NO_x$ in Cu-CHA Catalysts: Implications for the SCR-NO_x Reaction Mechanism. *ACS Catal.* **2019**, *9*, 2725−2738.

[19] Zhang, R.; Anderst. E.; Groden, K.; McEwen, J.-S. Modeling the Adsorption of NO and NH3 on Fe-SSZ-13 from First-Principles: A DFT Study. *Ind. Eng. Chem. Res.* **2018**, *57*, 40, 13396–13405.

[20] Zhang, R.; McEwen, J.-S.; Kollár, M.; Gao, F.; Wang, Y.; Szanyi, J.; Peden. C. H. F. NO Chemisorption on Cu/SSZ-13: A Comparative Study from Infrared Spectroscopy and DFT Calculations. *ACS Catal.* **2014**, *4*, 4093–4105.

[21] Uzunova, E. L.; Mikosch, H.; Hafner, J. Adsorption of NO on Cu-SAPO-34 and Co-SAPO-34: A Periodic DFT Study. *J. Phys. Chem. C* **2008**, *112*, 2632–2639.

[22] Concepción, P.; Boronat, M.; Millán, R.; Moliner, M.; Corma, A. Identification of Distinct Copper Species in Cu-CHA Samples Using NO as Probe Molecule. A Combined IR Spectroscopic and DFT Study. *Top. Catal.* **2017**, *60*, 1653–1663.

[23] Bhola, K.; Varghese, J. J.; Dapeng, L.; Liu, Y.; Mushrif S. H. Influence of Hubbard U Parameter in Simulating Adsorption and Reactivity on CuO: Combined Theoretical and Experimental Study. *J. Phys. Chem. C* **2017**, *121*, 21343−21353.

[24] Liu, C.; Kubota, H.; Toyao, T.; Maeno, Z.; Shimizu, K. Mechanistic insights into the oxidation of copper(i) species during NH_3-SCR over Cu-CHA zeolites: a DFT study. *Catal. Sci. Technol.* **2020**, *10*, 3586–3593.

[25] Chen, L.; Janssens T. W. V.; Grönbeck, H. A comparative test of different density functionals for calculations of NH_3-SCR over Cu-Chabazite. *Phys. Chem. Chem. Phys.* **2019**, *21*, 10923–10930.

[26] Liu, C.; Kubota, H.; Amada, T.; Toyao, T.; Maeno, Z.; Ogura, M.; Nakazawa, N.; Inagaki, S.; Kubota, Y.; Shimizu, K. Selective catalytic reduction of NO over Cu-AFX zeolites: mechanistic insights from in situ/operando spectroscopic and DFT studies. *Catal. Sci. Technol.* **2021**, *11*, 4459–4470.

[28] Isseroff, L.; Carter, E. Importance of reference Hamiltonians containing exact exchange for accurate one-shot GW calculations of Cu_2O. *Phys. Rev. B: Condens. Matter Mater. Phys.*, **2012**, *85*, 235142.

[29] Wang, L.; Maxisch, T.; Ceder, G. Oxidation energies of transition metal oxides within the GGA+U framework. Phys. Rev. B **2006**, *73*, 195107.

[30] Anggara, T.; Paolucci, C.; Schneider, W. F. Periodic DFT Characterization of NOx Adsorption in Cu-Exchanged SSZ-13 Zeolite Catalysts. J. Phys. Chem. C 2016, 120, 27934−27943

[31] Chen, L.; Falsig, H.; Janssens, T. V. W.; Grönbeck, H. Activation of Oxygen on (NH_3-Cu-NH_3)$^+$ in NH_3-SCR over Cu-CHA. *J. Catal.* **2018**, *358*, 179–186.

[32] Chen, L.; Janssens, T. V. W.; Skoglundh, M.; Grönbeck, H. Interpretation of NH_3-TPD Profiles from Cu-CHA Using First-Principles Calculations. Top. Catal. 2019, 62, 93–99.

[33] Moreno-González, M.; Millán, R.; Concepción, P.; Blasco, T.; Boronat, M. Spectroscopic Evidence and Density Functional Theory (DFT) Analysis of Low-Temperature Oxidation of Cu^+ to $Cu^{2+}NO_x$ in Cu-CHA Catalysts: Implications for the SCR-NOx Reaction Mechanism. *ACS Catal.* **2019**, *9*, 2725–2738.

[34] Janssens, T. V. W.; Falsig, H.; Lundegaard, L. F.; Vennestrom, P. N. R.; Rasmussen, S. B.; Moses, P. G.; Giordanino, F.; Borfecchia, E.; Lomachenko, K. A.; Lamberti, C.; Bordiga, S.; Godiksen, A.; Mossin, S.; Beato, P. A Consistent Reaction Scheme for the Selective Catalytic Reduction of Nitrogen Oxides with Ammonia. *ACS Catal.* **2015**, *5*, 2832–2845.

[35] Fernández, E.; Moreno-González, M.; Moliner, M.; Blasco, T.; Boronat, M.; Corma, A. Modeling of EPR Parameters for Cu(II): Application to the Selective Reduction of NO_x Catalyzed by Cu-Zeolites. *Top. Catal.* **2018**, *61*, 810–832.
